# MGIDI: a powerful tool to analyze plant multivariate data

**DOI:** 10.1186/s13007-022-00952-5

**Published:** 2022-11-12

**Authors:** Tiago Olivoto, Maria I. Diel, Denise Schmidt, Alessandro D. Lúcio

**Affiliations:** 1 Department of Plant Science, Federal University of Santa Catarina, Florianópolis, SC 88034-000 Brazil; 2 Departament of Plant Science, Federal University of Pampa, Itaqui, RS 97650-000 Brazil; 3 Departament of Agronomic and Environmental Sciences, Federal University of Santa Maria, Frederico Westphalen, RS 98400-000 Brazil; 4 Departament of Plant Science, Federal University of Santa Maria, Santa Maria, RS 97105-900 Brazil

**Keywords:** Fragaria $\times$ ananassa Dusch, Organic substrates, Multivariate selection, Substrate cultivation, Fruit quality, MGIDI

## Abstract

**Background:**

Commonly, several traits are assessed in agronomic experiments to better understand the factors under study. However, it is also common to see that even when several traits are available, researchers opt to follow the easiest way by applying univariate analyses and *post-hoc* tests for mean comparison for each trait, which arouses the hypothesis that the benefits of a multi-trait framework analysis may have not been fully exploited in this area.

**Results:**

In this paper, we extended the theoretical foundations of the multi-trait genotype-ideotype distance index (MGIDI) to analyze multivariate data either in simple experiments (e.g., one-way layout with few treatments and traits) or complex experiments (e.g., with a factorial treatment structure). We proposed an optional weighting process that makes the ranking of treatments that stands out in traits with higher weights more likely. Its application is illustrated using (1) simulated data and (2) real data from a strawberry experiment that aims to select better factor combinations (namely, cultivar, transplant origin, and substrate mixture) based on the desired performance of 22 phenological, productive, physiological, and qualitative traits. Our results show that most of the strawberry traits are influenced by the cultivar, transplant origin, cultivation substrates, as well as by the interaction between cultivar and transplant origin. The MGIDI ranked the Albion cultivar originated from Imported transplants and the Camarosa cultivar originated from National transplants as the better factor combinations. The substrates with burned rice husk as the main component (70%) showed satisfactory physical proprieties, providing higher water use efficiency. The *strengths and weakness view* provided by the MGIDI revealed that looking for an ideal treatment should direct the efforts on increasing fruit production of Albion transplants from Imported origin. On the other hand, this treatment has strengths related to productive precocity, total soluble solids, and flesh firmness.

**Conclusions:**

Overall, this study opens the door to the use of MGIDI beyond the plant breeding context, providing a unique, practical, robust, and easy-to-handle multi-trait-based framework to analyze multivariate data. There is an exciting possibility for this to open up new avenues of research, mainly because using the MGIDI in future studies will dramatically reduce the number of tables/figures needed, serving as a powerful tool to guide researchers toward better treatment recommendations.

**Supplementary Information:**

The online version contains supplementary material available at 10.1186/s13007-022-00952-5.

## Background

Agronomic experiments aim to test the effects of levels or combinations of factor levels on plant traits to understand the phenomena under study [1–3]. At the end of an experiment, the researchers often have a spreadsheet with dozens of columns (one for each trait), that need to be analyzed to make inferences on the treatment (rows) performance. In its vast majority, the analysis of such data sets involves first performing an analysis of variance (ANOVA) for each trait to test the null hypothesis that the effects of treatments [($\hat{t}_i$] are null [i.e., $H_0:\hat{t}_i = 0 \forall _i$], and if $H_0$ is rejected, implementing a *post-hoc* test to identify which treatment is significantly different from which other treatment [4–6]. The ambiguity presented in some tests such as Tukey’s Honest Significant Difference (HDS) test, however, can lead to limited inferences. For example, a study using HDS to compare phenolic compounds among 32 strawberry cultivar [7] have observed means followed by “cdefghij”. Another similar study which aimed to determine the carbohydrates (sugars), organic acids, total phenol content, individual phenolic compounds, total antioxidant capacity, and volatile aroma substances on ten strawberry cultivars [8] used Fisher’s Least Significant Difference (LSD) to compare the 20 compounds among cultivars. In both cases, the main goal would identify superior cultivars, but it was extremely difficult to rank the treatments based on their performance on multiple traits.

Experienced researchers often keep in mind a set of plant traits that an “ideal” treatment should provide. For strawberries, for example, it is sought for treatments that present productive precocity and a high rate of fruit production [9]. Sensory characteristics such as total soluble solids content, total titratable acidity, and their relationship, are essential to winning over consumers, as well as physical coloring characteristics [10–12]. In addition, flesh firmness or resistance to penetration is essential to increase the shelf-life of fruits [11, 13]. For strawberries, treatments that provide optimal chemical characteristics, such as high flavor and aroma, might be poorly productive, which is not desired by producers. The reciprocal is also true. A study showed that the Camarosa cultivar showed higher production, but lower sugar content and higher acidity [11], which may not be attractive for fresh consumption, being more suitable for the processing industry. In this context, the use of multivariate techniques would be strongly suggested to take into account the correlation structure between traits.

Multivariate exploratory techniques such as Principal Component Analysis (PCA) and Linear Discriminant Analysis (LDA) have been extensively used for dimensionality reduction and visual approximation of a two-way table involving treatments and plant traits [14–16]. Although these approaches easily provide an overview of the relationships between traits, ranking the treatments based on trait values remains a challenge. Therefore, innovative multivariate approaches are needed to provide a better strategic treatment ranking based on multiple traits.

In the context of multiple selection, linear indexes can be used [17]. One fragility of linear selection indexes is the collinearity often observed in the set of assessed traits, which can bias the coefficients of multiple regression, and thus erode selection gains [18]. To overcome this fragility, the multi-trait genotype-ideotype distance index (MGIDI) has been proposed [19]. The MGIDI was originally designed for selecting genotypes in plant breeding based on information on multiple traits and has been successfully used to select superior genotypes [19–24].

The application of the MGIDI in the context of treatments recommendation (e.g., fungicides, fertilizers, management strategies) in plant experiments is promising but not yet explored in recent literature. Therefore, our main aim here is to extend the theoretical foundations of the MGIDI to analyze plant experiments with information on multiple traits, identifying treatments that provide favorable performance for most of the traits under study. An adaptation of the index is proposed by including optional weights for traits that are assumed to be more important for the treatment ranking. A simple example using simulated data is used to show how the index can be used even with a few traits/treatments. A real strawberry experiment data that aims to select better factor combinations (namely, cultivar, transplant origin, and substrate mixture) based on the desired performance of 22 phenological, productive, physiological, and qualitative traits are used to show the potentialities of the index. Our results open up new horizons about how the MGIDI can be used beyond the plant breeding context, serving as a powerful tool to analyze plant multivariate data.

## Methods

### Simulated data

A dataset with five treatments and three accessed traits was simulated using the function g_simula() from the R [25] package metan [26]. This data was analyzed using two strategies. In the first, the three traits were analyzed using an ANOVA model considering a randomized complete block design. Then, pairwise multiple comparisons based on the Tukey test were performed using the emmeans R package [27]. In the second, the MGIDI was used to rank the treatments according to three selection strategies, namely, (1) when higher values are desired for all the traits; (2) when lower values are desired for the two first traits, and higher values for the last trait; and (3) when lower values are desired for all the traits.

### Real data: location and cultivation environment

Data on a strawberry experiment was used to demonstrate the use of the MGIDI for ranking treatments based on multiple traits. The experiment was conducted at the Federal University of Santa Maria, Frederico Westphalen campus (27 $^{\circ }$23’S, 53 $^{\circ }$25’O, 493 masl). The climate is *Cfa* according to Köppen’s classification, where the three coldest months of the year have temperatures of $-3$ to 18 $^{\circ }$C, with an air average temperature in the warmest month greater than or equal to 22 $^{\circ }$C, and precipitation uniformly distributed during the year [28].

An open, substrate-based cultivation system was carried out inside an experimental greenhouse (20-m length, 10-m width, and 3.5-m height). The strawberry transplants were transplanted into white, 150-$\mu$m thickness tubular plastic bags, kept on wooden benches 0.8 meters above the ground. Drip irrigation and fertigation were performed. The frequency of irrigation and the formulation of fertigation was carried out according to [29]. The nutrient dose for fertigation is presented in Additional file 1/InternalRef>: Table 1.1. The electrical conductivity (EC) of the nutrient solution was 1.8 mS cm$^-1$. The irrigation frequency, as well as the time of each irrigation pulse, were adjusted based on the solution drained from the substrate, monitoring the EC of the nutrient solution.

### Plant material and experimental design

The experiment was conducted in a randomized complete block design with four replications and 8 strawberry plants per replication. The treatment layout was a three-way factorial treatment structure with two cultivars [Albion (ALB)—neutral days, and Camarosa (CAM) —short days], two transplants origins [National (NAT), and Imported (IMP)], and four organic substrates mixes (S1: Sugarcane bagasse + organic compost; S2: Sugarcane bagasse + commercial substrate - CarolinaCarolina^®^; S3: Rice husk + organic compost; and S4: Rice husk + commercial substrate - CarolinaCarolina^®^). See details of the substrate mixes in Additional file 1: Table 2.1.

The transplants of National origin came from Agudo, RS, Brazil (29 $^{\circ }$62’S, 53 $^{\circ }$22’O, 83 masl). The Imported transplants were produced in the Patagônia Agrícola SA nursery, located in the municipality of El Maitén, Argentina (42 $^{\circ }$3’S, 71 $^{\circ }$10’O, 720 masl). The transplants of cultivars Albion (National) and Camarosa (National and Imported) were transplanted on May 26, 2015. The cultivar Albion imported was transplanted on June 8, 2015.

### Assessed traits

The harvests began in the maturation stage and were carried out twice a week. Through the production cycle, a total of 22 phenological, productive, physiological, and qualitative traits were assessed.

#### Phenological traits

We evaluated the following phenological traits: phyllochron (PHYL, $^{\circ }$C day leave$^{-1}$ estimated as the inverse of the slope of the linear regression between the number of leaves in the crown against the accumulated thermal sum. A leaf was counted when the leaflets did not touch each other; number of days for the beginning of flowering (NDBF, days), computed when the first flower of the block was open; number of days for full flowering (NDFF, days), computed when all plants in the block had opened flowers; number of days for the beginning of harvest (NDBH, days).

#### Productive traits

The fruits from each plot were classified as commercial and non-commercial and evaluated separately. We considered as non-commercial, fruits deformed or with a weight of less than 6 grams. In this stage, the following productive traits were assessed: number of commercial fruits (NCF, fruits plant$^{-1}$); number of non-commercial fruits (NCF, fruits plant$^{-1}$); total number of fruits (TNF, fruits plant$^{-1}$); weight of commercial fruits (WCF, g plant$^{-1}$); weight of non-commercial fruits (WNCF, g plant$^{-1}$); total weight of fruits (TWF, g plant$^{-1}$); average weight of commercial fruits (AWCF, g fruit$^{-1}$); average weight of non-commercial fruits (AWNCF, g fruit$^{-1}$); overall average weight of fruits (OAWF, g fruits$^{-1}$); fruit yield (FY, Kg ha$^{-1}$).

#### Physiological traits

The water use efficiency (WUE, l$^{-1}$) was calculated as the ratio between the amount of water used in the entire duration of the experiment and the total fruit production for each plant.

#### Qualitative traits

Quality-related traits were assessed in three moments throughout the production cycle and averaged to smooth possible punctual season effects. The traits assessed were: total titratable acidity (TA, mg citric acid 100 g$^{-1}$) performed by titration with a standardized NaOH solution (0.1 mol L$^{-1}$), total soluble solids (TSS, $^\circ$Brix) using a manual refractometer (± 2% accuracy), the ration between TSS and TA (TSS/AT) calculated using the quotient between the total soluble solids content and the titratable acidity; flesh firmness (FIRM), determined using a bench penetrometer with a 6 mm plunger.

Pulp coloration was evaluated by chroma, hue angle, and lightness in CIELCh color space (cylindrical coordinates) after conversion from the CIEL$^*$a$^*$b$^*$ color space (cartesian coordinates). First, fruit color was expressed as three values [L$^*$, for the lightness from black (0) to white (100), a$^*$ from green (−) to red (+), and b$^*$ from blue (−) to yellow (+)]. L$^*$, a$^*$, and b$^*$ were determined with a colorimeter calibrated with a standard white ceramic plate. The conversion of a$^*$ and b$^*$ to C$^*$ (CHROMA, relative saturation) and h$^{\circ }$ (H, angle of the hue in the CIELab color wheel) was done using the following formulas: $C^* = \sqrt{a^{*2} + b^{*2}}$; and $h^{\circ } = \arctan {(b^*/a^*)}$. The CIEL$^*$a$^*$b$^*$ lightness (L$^*$) remained unchanged.

### Defining an *ideal* treatment

In this crucial step, we define an *ideal* treatment, i.e., the one that would provide desired values for all studied traits. Based on previous knowledge, and assuming that a grower focuses on the early production of strawberries, an ideal treatment should provide: (i) plants with high water use efficiency that presents a short period between planting and beginning of flowering/harvesting, with low phyllochron values, i.e., a high number of leaves per accumulated thermal sum. Thus, lower values for WUE, NDBF, NDFF, NDBH, and PHYL are desired); (ii) high fruit yield with a higher number of commercial fruits with a high average weight of fruits, which are defined by higher values for NCF, TNF, WCF, TWF, AWCF, OAWF, and FY); (iii) lower number of non-commercial fruits of low average weight, which are defined by lower values for NNCF, WNCF, and AWNCF); and (iv) sweet, firm fruits with lower acidity and ideal external and internal red color, which are defined by lower values for TA and higher values for TSS, TSS/TA, FIRM, L, CHROMA, and H).

### Statistical analysis

#### Estimated means

To avoid pseudo-replication, plants within replicates were averaged. The overall effects of cultivar (CUL), origin (ORI), and substrate (SUB) on the accessed traits were analyzed using a 3-way multivariate analysis of variance (MANOVA) using the function manova() from the R software, according to the following model.

1 $$\begin{aligned} \begin{aligned} {{\textbf {Y}}}={{\textbf {Xb}}}+{{\textbf {e}}} \end{aligned} \end{aligned}$$

where **Y** is an $n \times p$ matrix of response variables where $n$ is the number of plots, i.e., the combination of levels for substract ($s$ = 1, 2, ..., 4), cultivar ($c$ = 1, 2), origin ($o$ = 1, 2), and block ($b$ = 1, 2, ..., 4)), and $p$ the number of accessed traits; **X** is an $n \times m$ model matrix, beig $m = s\times c \times o$; **b** is an $m \times p$ matrix of model coefficients; and **e** is an $n \times p$ matrix of model residuals.

Pillai’s trace was used as the test of significance as suggested by Hand and Taylor [30]. The predicted values from significant terms were further used to create a two-way table (${{\textbf {X}}}_{ij}$) containing the estimated means for each treatment in rows and traits in columns.

#### Reescaled means

Based on the knowledge of an *ideal* treatment, we reescaled ${{\textbf {X}}}_{ij}$ to obtain $r{{\textbf {X}}}_{ij}$ as proposed by [31].

2 $$\begin{aligned} r{{{\textbf {X}}}_{ij}} = \frac{{{\eta _{nj}} - {\varphi _{nj}}}}{{{\eta _{oj}} - {\varphi _{oj}}}} \times ({\theta _{ij}} - {\eta _{oj}}) + {\eta _{nj}} \end{aligned}$$

where ${\eta _{nj}}$ and ${\varphi _{nj}}$ are the new maximum and minimum values for the trait *j* after rescaling, respectively; ${\eta _{oj}}$ and ${\varphi _{oj}}$ are the original maximum and minimum values for the trait *j*, respectively, and ${\theta _{ij}}$ is the original value for the *j*th trait of the *i*th treatment. For NNCF, WNCF, AWNCF, WUE, NDBF, NDFF, NDBH, PHYL, and TA in which lower values are desired, ${\eta _{nj}} = 0$ and ${\varphi _{nj}} = 100$ were used. For all the other traits in which higher values are desired, ${\eta _{nj}} = 100$ and ${\varphi _{nj}} = 0$ were used. In the rescaled two-way table ($r{{{\textbf {X}}}_{ij}}$), all columns have a 0-100 range in which 100 is the most desired value. Thus, the ideal treatment would be the one with 100 for all traits after rescaling.

#### Factor analysis

The MGIDI [19] was used to rank the treatments based on the desired values of the studied trait. First, factor analysis was computed with ($r{{{\textbf {X}}}_{ij}}$) to account for the correlation structure and dimensionality reduction of the data, as follows

3 $$\begin{aligned} {{\textbf {X }}}= \mu + {{\textbf {Lf}}} + \varepsilon \end{aligned}$$

where **X** is a $p\times 1$ vector of rescaled observations; ${{{\varvec{\mu }}}}$ is a $p\times 1$ vector of standardized means; **L** is a $p\times f$ matrix of factorial loadings; **f** is a $p\times 1$ vector of common factors; and ${{{\varvec{\varepsilon }} }}$ is a $p\times 1$ vector of residuals, being *p* and *f* the number of traits and common factors retained, respectively. The eigenvalues and eigenvectors are obtained from the correlation matrix of $r{{{\textbf {X}}}_{ij}}$. The initial loadings are obtained considering only factors with eigenvalues higher than one. Then, the *varimax* [32] rotation criteria is used for the analytic rotation and estimation of final loadings. Finally, the scores are computed as follows:

4 $$\begin{aligned} {{\textbf {F}}} = {{\textbf {Z}}}({{\textbf {A}}}^{{\textbf { T}}}{{\textbf {R}}}^{{\textbf {-1}}})^{{\textbf {T}}} \end{aligned}$$

where ${{\textbf {F}}}$ is a $g\times f$ matrix with the factorial scores; ${{\textbf {Z}}}$ is a $g\times p$ matrix with the (rescaled) standardized means; ${{\textbf {A}}}$ is a $p\times f$ matrix of canonical loadings, and ${{\textbf {R}}}$ is a $p\times p$ correlation matrix between the traits. *g*, *f*, and *p* represents the number of treatments, factors retained, and analyzed traits, respectively. The number of factors retained was based on the Guttman-Kaiser criterion [33] following the eigenvalues-greater-than-one rule.

#### Multi-trait Genotype-Ideotype Distance Index

After the factor analysis, the MGIDI is computed as the Euclidean distance between the scores of treatments and the ideal treatment was computed as follows [19]:

5 $$\begin{aligned} MGID{I_i} = {\left[ {\sum \limits _{j = 1}^f {{{\left( {{\gamma _{ij}} - {\gamma _j}} \right) }^2}} } \right] ^{0.5}} \end{aligned}$$

where $MGIDI_i$ is the multi-trait genotype-ideotype distance index for the *i*th treatment; $\gamma _{ij}$ is the score of the *i*th treatment in the *j*th factor ($i = 1, 2, ..., t$; $j = 1, 2, ..., f$), being *t* and *f* the number of treatments and factors, respectively; and $\gamma _j$ is the *j*th score of the ideal treatment. The treatment with the lowest MGIDI is then closer to the ideal treatment and therefore presents desired values for all the *p* traits.

By definition [Eq. (2)], the “ideal” treatment would have 100 for all analyzed traits after the rescaling process. Therefore, $\gamma _j$ is defined as the scores [Eq. (4)] of a $1\times p$ vector ${{\textbf {I}}}$ such that ${{{\textbf {I}}}} = [100, 100, ..., 100]$.

In experiments accessing several plant traits, it is common that some traits have more importance than others. For example, for wheat experiments, grain yield and grain mass per spike are two common analyzed traits and for both of them, higher values are desired. Given the original MGIDI index, both grain yield and the number of grains per spike have the same weight in the MGIDI computation. It would be desired, however, to give a higher weight to yield, prioritizing treatments with a higher grain yield, even if they have a lesser number of grains per spike. Another example would be the selection of strawberry cultivars based on multiple traits [34]. Total fruit production is a relevant trait, but the production of commercial fruits (fruits not deformed and with more than 6 g) becomes more important since only commercial fruits are marketable.

Here, we propose an optional weighting procedure that will allow giving higher weights for traits that are assumed to have more importance in the treatment ranking. The weighting process is done by simply multiplying each element in $\gamma _j$ by the corresponding element in the weight vector $\theta _j$, with the same length of $\gamma _j$. By default (original MGIDI), a vector of 1’s is considered, meaning all traits have the same weight. If a higher value is used (e.g., 5), then, the factor that includes the trait with higher weight will have a proportionally higher value, shrinking the Euclidean distance [Eq. (5)] of the treatment that has a higher value for that factor (See an example in Additional file 1: Supplementary code 3). Here, the selection of the better treatments was performed considering a higher weight (4) for the number and weight of commercial fruits.

#### Strengths and weaknesses

The proportion of the MGIDI of the *i*th treatment explained by the *j*th factor ($\omega _{ij}$) was used to show the strengths and weaknesses of the treatments and was computed as:

6 $$\begin{aligned} \omega _{ij} = \frac{\sqrt{D_{ij}^2}}{\sum \limits _{j = 1}^f\sqrt{D_{ij}^2}} \end{aligned}$$

where $D_{ij}$ is the distance between the *i*th treatment and ideal treatment for the *j*th factor. Low contributions of a factor suggest that the traits within such a factor are close to the ideal treatment.

#### Principal component analysis

To visually understand the relationships between trait and their association with the treatments, we conducted a Principal Component Analysis (PCA) with ${{{\textbf {X}}}}_{ij}$ containing the treatments in rows and traits in columns. A biplot was produced with the function fviz_pca_biplot() from the R package factoextra.

Data manipulation and the index computation were performed in the R Software version 4.1.0 [25] using the package metan v1.17.0 [26] and the ecosystem of packages Tidyverse [35].

## Results

### Simulated data

**Fig. 1:**
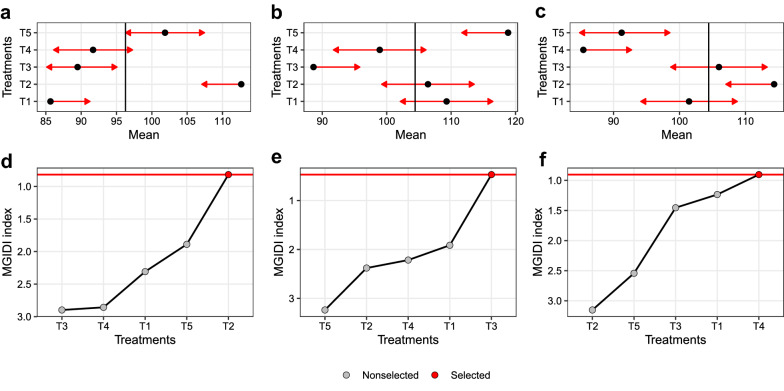
Pairwise comparisons for V1 (**a**), V2 (**b**), V3 (**c**), and the MGIDI index for three selection strategies, namely, desired higher values for all traits (**d**), lower values for V1 and V2 and higher values for V3 (**e**), and lower values for all traits (**f**)

Figure 1 shows the pairwise mean comparison for the three simulated traits and the treatment ranking based on the MGIDI. For the first scenario, where higher values are desired for all the traits, T2 was the first ranked by the MGIDI (Fig. 1d). Looking at the mean comparisons, it can be seen that T2 would have “a” for all the traits (Fig 1a–c). In the second scenario, T3 was ranked as the better treatment (Fig. 1e), since it presented lower values for the first two traits (Fig. 1a, b) and higher values for the last trait (Fig. 1c). In the last scenario, T4 was considered the better treatment (Fig. 1f) and would have “c” for all three traits. Considering a more complex example with 10 traits accessed in 75 treatments (Additional file 1: Supplementary code 2.2) a pairwise mean comparison performed for each trait becomes worthless since there is no way to quickly rank the treatments based on the desired value of each trait. When the MGIDI is used with the estimated marginal means of a MANOVA (Additional file 1: Supplementary code S2.2.2), the treatment ranking becomes easier and takes into account the desired sense of selection (i.e., if higher or lower values are better).

### Multivariate test statistic

The multivariate analysis of variance (Table 1) indicated a significant $p \le 0.05$ main effect for substrate (SUB), origin (ORI), and cultivar (CUL). For the two-way interaction terms, only the interaction ORI x CULT was significant $p \le 0.05$, indicating that the response of the cultivar is dependent on its origin. The three-way interaction term (CUL x ORI x SUB) was not significant.

**Table 1:** Multivariate analysis of variance of the effects of substrate, origin, and cultivar on the analyzed traits

Effect	Value	F (Pilai’s trace)	Hypothesis DF	Error DF	P-value
Intercept	1.000	10522.936	22	27	1.20E-47
Substrate (SUB)	1.809	2.004	66	87	0.0012
Origin (ORI)	0.855	7.249	22	27	1.66E-06
Cultivar (CUL)	0.923	14.700	22	27	6.70E-10
SUB × ORI	1.226	0.911	66	87	0.6525
SUB × CUL	1.523	1.359	66	87	0.0898
ORI × CUL	0.692	2.752	22	27	0.0067
SUB × ORI x CUL	1.339	1.063	66	87	0.3923

Based on the significance of factors, the MGIDI was computed for (i) a two-way table containing in the rows the combination of cultivars and origin and in columns the traits, and (ii) a two-way table with substrates in the rows and traits in columns. For both cultivar x origin interaction and substrate, three factors (FA) were retained, explaining 100% of the total variance (Table 2). Different traits were grouped into each factor depending on the factor studied.

### Cultivar × origin interaction

The 22 traits were grouped into the factors (FA) as follows (Table 2): In FA1, most of the productive-related traits AWNCF, FY, NCF, TA, TNF, TSS_TA, TWF, WCF, WUE with positive loadings, and NNCF with negative loadings; In FA2, the traits CHROMA and PHYL (with positive loadings) and AWCF, H, L, OAWF, and WNCF (with negative loadings); In FA3, the phenological-related traits NDBF, NDBH, NDFF, and the quality-related traits TSS and FIRM. Loadings resulting from an orthogonal rotation range from − to + 1 and are the correlation coefficients between each trait and the factor.

**Fig. 2:**
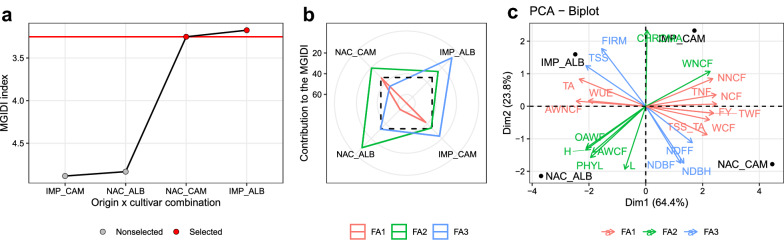
Treatment ranking based on the MGIDI (**a**), the strengths and weaknesses view of the treatments (**b**), and the biplot of the principal component analysis performed with the studied traits for the cultivar × origin interaction (**c**). Strengths and weaknesses are shown as the proportion of each factor on the computed multi-trait genotype-ideotype distance index. The smallest the proportion explained by a factor (closer to the external edge), the closer the traits within that factor are to the “ideal” treatment. The selected factor combinations were used to compute the selection differentials

Figure 2a shows the treatment ranking according to the MGIDI. The better factor combinations were the Albion cultivar with transplants from the imported origin and the Camarosa cultivar with transplants from the National origin. The contribution of each factor to the MGIDI (Fig. 2b) is ranked from the most contributing factor (close to the plot center) to the less contributing factor (close to the plot edge). This suggests that Albion of imported origin has strengths related to FA3, i.e., higher values of TSS and FIRM and earlier harvest start, indicated by lower values of NDDFF, NDBF, and NDBH (Fig. 2c; Additional file 1: Figures 1.2-1.4).

**Table 2:** Eigenvalues, explained variance, and factorial loadings after varimax rotation obtained in the factor analysis

Cultivar × origin				Substrate			
Trait	FA1	FA2	FA3	Trait	FA1	FA2	FA3
AWNCF	**0.63**	0.49	0.60	AWCF	**0.63**	− 0.48	− 0.62
FY	**0.93**	0.23	0.29	FIRM	**1.00**	− 0.06	0.05
NCF	**0.84**	0.42	0.35	FY	**0.96**	− 0.23	− 0.15
NNCF	**− 0.72**	− 0.67	− 0.17	NCF	**0.96**	− 0.21	− 0.18
TA	**0.84**	0.10	0.54	NDBF	**0.74**	0.14	− 0.66
TNF	**0.80**	0.52	0.29	NDBH	**0.85**	− 0.46	− 0.27
TSS_TA	**0.97**	0.09	0.24	NDFF	**0.91**	− 0.33	− 0.25
TWF	**0.93**	0.23	0.29	NNCF	**− 0.99**	0.03	− 0.17
WCF	**0.93**	− 0.05	0.37	OAWF	**0.66**	− 0.63	− 0.39
WUE	**1.00**	0.04	0.03	PHYL	**0.88**	− 0.01	− 0.47
AWCF	− 0.31	**− 0.93**	− 0.19	TNF	**0.98**	− 0.18	− 0.13
CHROMA	− 0.29	**0.89**	− 0.36	TWF	**0.96**	− 0.23	− 0.15
H	− 0.63	**− 0.78**	− 0.01	WCF	**0.88**	-0.34	− 0.33
L	0.19	**− 0.98**	0.01	WNCF	**− 0.83**	0.21	− 0.52
OAWF	− 0.47	**− 0.86**	− 0.17	WUE	**0.88**	− 0.33	− 0.35
PHYL	0.60	**0.79**	− 0.12	CHROMA	− 0.26	**0.97**	− 0.03
WNCF	− 0.65	**− 0.75**	− 0.14	H	− 0.06	**− 0.98**	0.21
FIRM	− 0.32	0.10	**− 0.94**	L	0.43	**− 0.87**	− 0.23
NDBF	− 0.11	0.07	**− 0.99**	TA	0.17	**− 0.71**	− 0.69
NDBH	− 0.17	0.07	**− 0.98**	TSS	− 0.26	**0.95**	0.17
NDFF	− 0.16	− 0.24	**− 0.96**	AWNCF	0.26	0.19	**− 0.95**
TSS	− 0.56	− 0.10	**− 0.82**	TSS_TA	− 0.09	− 0.37	**− 0.93**
Eigenvalues	14.17	5.24	2.58	Eigenvalues	14.93	3.95	3.12
Variance (%)	64.43	23.82	11.75	Variance (%)	67.85	17.97	14.18
Cummulative (%)	64.43	88.25	100	Cummulative (%)	67.85	85.82	100

**Fig. 3:**
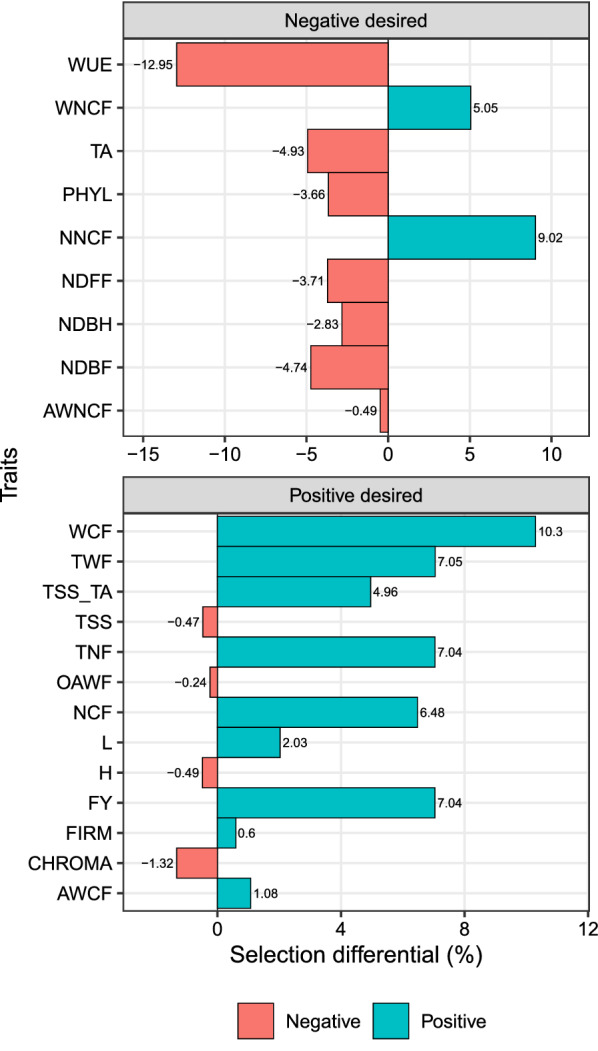
Selection differentials (%) for productive, qualitative, and physiological strawberry traits obtained with the selection of Imported Albion and National Camarosa cultivars. Facets groups the traits based on the desired selection differentials

The MGIDI provided desired selection differentials (SD) for 16 out of 22 studied traits (Fig. 3). For traits in which lower values are desired, the selection differentials ranged from − 12.95% (WUE) to 9.02% (NNCF). For the traits in which higher values are desired, the SD ranged from − 1.32% (CHROMA) to 10.3% (WCF). The positive SD observed for the productive-related traits were mainly due to the Camarosa cultivar of National origin (Additional file 1: Figures 2.4, 2.7, 2.9, 2.12, and 2.13), which is confirmed by the strengths related to FA1 of this treatment (Fig. 2b).

### Substrate main factor

Analyzing the substrate main factor, the 22 traits were grouped into the factors (FA) as follows (Table 2): In FA1, the traits AWCF, FIRM, FY, NCF, NDBF, NDBH, NDFF, OAWF, PHYL, TNF, TWF, WCF, and WUE (with positive loadings); NNCF and WNCF with negative loadings. The FA2 grouped the traits CHROMA and TSS (positive loading), and H, L, and TA (negative loadings). Finally, the FA3 grouped the traits AWNCF and TSS_TA (Fig. 3).

**Fig. 4:**
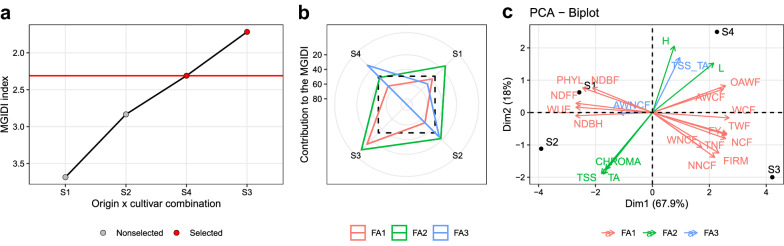
Treatment ranking based on the MGIDI (**a**), the strengths and weaknesses view of the treatments (**b**), and the biplot of the principal component analysis performed with the studied traits for the substrate factor (**c**). Strengths and weaknesses are shown as the proportion of each factor on the computed multi-trait genotype-ideotype distance index. The smallest the proportion explained by a factor (closer to the external edge), the closer the traits within that factor are to the ’ideal’ treatment. The selected substrates were used to compute the selection differentials

**Fig. 5:**
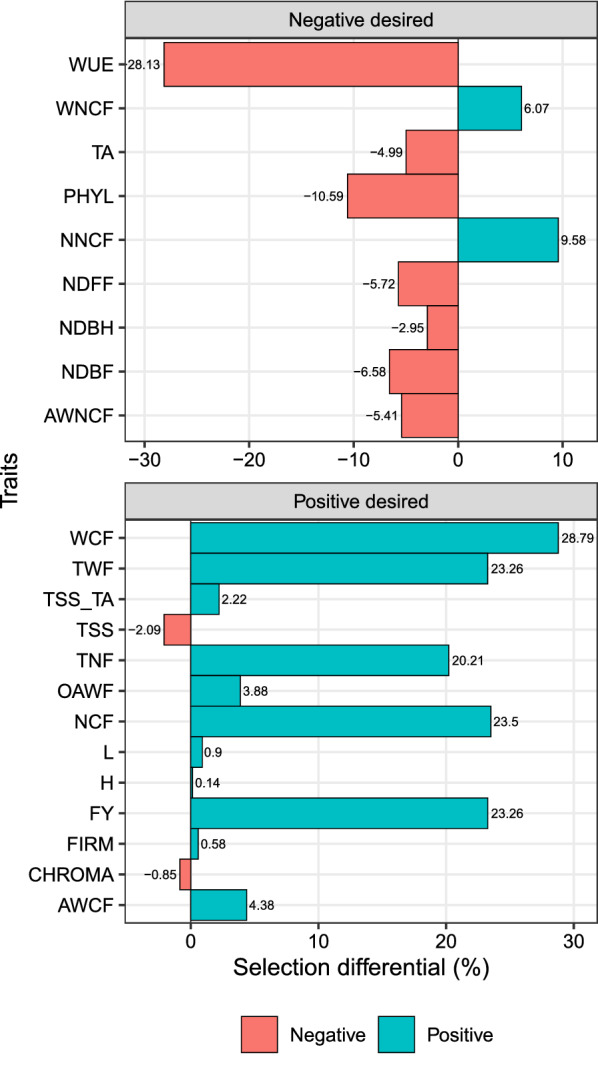
Selection differentials (%) for productive, qualitative, and physiological strawberry traits obtained with the selection of Imported Albion and National Camarosa cultivars. Facets groups the traits based on the desired selection differentials

The better-ranked substrates were S3 and S4. These substrates have as a common ingredient burnt rice husk (70%). The strengths and weaknesses view (Fig. 4b) shows that S4 has strengths related to FA3, i.e., lower values for AWNCF (Fig. 4c; Additional file 1: Figure 1.1) and higher TSS/TA ratio (Fig. 4c; Additional file 1: Figure 2.11). Both substrates provided lower values for WUE, indicating that compared to the other substrates, a smaller amount of water is necessary to produce one Kg of fruit (Fig. 5, Additional file 1: Figure 1.9).

The SDs computed with these substrates were in desired sense for 18 out of 22 analyzed traits. For traits in which lower values are desired, the SD ranged from − 28.13% (WUE) to 9.59% (NNCF). For traits in which higher values are desired, the SD ranged from − 2.09% (TSS) to 28.79% (WCF).

**Table 3:** Comparison between MGIDI, PCA, and Linear indexes regarding several aspects. Strengths are indicated by ’+’ and weaknesses by ’−’

Aspect	MGIDI	PCA	Linear Index
Graphical Interpretation	++	+++	- - -
Implementation in Free and Open Source softwares	+++	+++	+++
Computational power	++	+++	+++
Processing time	+	+++	+++
Collinearity issues	+++	+++	- - -
Easy treatment ranking	+++	-	++
Strengths and weaknesses	+++	-	- - -
Variable’s weighting in treatment ranking	+++	- - -	- - -

## Discussion

### Why a multi-trait framework analysis?

Our experimental results suggest that most of the productive, phenological, and qualitative traits in strawberries are influenced by the cultivar used, transplant origin, cultivation substrates, and the interaction between cultivar and origin of transplants. Previous studies also reported a significant effect of cultivars for physicochemical traits of fruits [36], phyllochron and phenology [37] and external morphanatomy [38]. This is explained because the growth and development of strawberries are regulated by the complex interaction of factors, such as temperature [39], light-temperature interactions [40], daylength conditions [41], and cultivation substrate [42].

As new studies evaluate a growing amount of traits aiming at better explaining the phenomena [42–44], the challenge of summarizing the complex results into an easy-to-handle and practical recommendation arises. Differently from an approach in which a *post-hoc* test—e.g., Tukey’s honest significance test—is used, we have shown here how to rank treatments in agronomic experiments based on the values of several traits (Figs. 1, 2a, and 4). In our example, this approach indicated that imported transplants of Albion strawberry cultivar growing in substrates where the main component (70%) is burnt rice husk provides desired values for most of the 22 productive, qualitative, physiological, and phenological traits assessed in the experiment. Therefore, both for simple (i.e., a small number of treatments and traits) or complex (i.e., a large number of treatments and traits) experiments, the use of the MGIDI is an interesting alternative to pairwise multiple comparisons tests (See a numerical example in Additional file 1: Supplementary codes 2.2).

Based on our results, a grower could cultivate part of their area with Imported transplants of Albion cultivar to take advantage of earlier fruit production (approximately 64 days after transplant) and supply to the consumer market fruits with higher flesh firms and total soluble solids (Fig. 2b). Our results corroborate with [36], which studying 13 strawberry cultivars observed that Albion presents high values for fresh firmness and $^\circ$Brix. Flesh firmness is very important since it allows identifying treatments that produce fruits with longer shelf life and more resistance to transport damages and quality loss [45]. The content of total soluble solids defines the sweetness in fruits [46], which combined with components such as strawberry furanone defines the fruity flavors that characterize a fresh strawberry [47]. Therefore, the production of this treatment could be focused on the market for *in natura* consumption.

In another part of the area, the grower could use National transplants of Camarosa cultivar to take advantage of higher fruit production. The higher number and weight of non-commercial fruits observed in this treatment (Additional file 1: Figs. 1.5 and 1.8, respectively) may result in difficulties in fruit marketing since small fruits are not desired by consumers [48]. Another weakness of this treatment is related to FA3 (Fig. 2b), where fruits with less FIRM and TSS may not be well-accepted for fresh consumption.

The treatment ranking based on multiple traits (Fig. 2a) combined with the strengths and weaknesses view (Fig. 2b) is a powerful tool that can be used to guide researchers towards better treatment recommendations.

### Univariate selection is not efficient

Considering a univariate recommendation based on FY solely, the Camarosa cultivar would be recommended since, on average, it is more productive (Additional file 1: Fig. 2.4). The origin of transplants, however, must be taken into consideration. The recommendation of Camarosa with transplants of National origin based on FY would result in higher fruit production. Previous studies also reported a greater total fresh fruit mass of Camarosa compared to Albion [49]. This is explained mainly because Camarosa can increase the emission of crowns compared to Albion, resulting in a higher number of leaves. For this reason, at the end of the cycle, the Camarosa cultivar would probably have a higher total number of leaves per plant compared to Albion [37, 38].

The univariate selection, however, would result in desired values for only 11 out of the 22 analyzed traits (Additional file 1: Table 3.1); some examples are related to productive traits such as the higher number and weight of non-commercial fruits (Additional file 1: Figs. 1.5 and 1.8, respectively), qualitative traits such as lower flesh firmness and total soluble solids (Additional file 1: Figs. 2.3 and 2.10, respectively), and phenological traits such as the late beginning of the harvest (Additional file 1: Figs. 1.4). Previous studies have also shown that the beginning of flowering is similar for Camarosa and Albion, but full flowering is achieved later in the Albion cultivar [49]; this characteristic may be undesired by growers.

### Alternative substrates in strawberry cultivation

The water use efficiency was highly influenced by the cultivation substrate and less by the cultivar used (Additional file 1: Fig. 1.9). The high water use efficiency in substrates with burnt rice husks seems to be related to the higher water retention capacity at lower tensions (Additional file 1: Fig. 3), which can be explained by its favorable physical proprieties such as lower total porosity and aeration space, resulting in higher amounts of readily available water (Additional file 1: Table 2.2). These physical characteristics are crucial for root growth and plant development due to its promotion of an adequate air/water balance during plant cultivation [50].

Strawberry requires a high amount of water and although cultivars with more leaves can produce a higher amount of fruits, a higher total leaf area per plant can lead to higher transpiration rates, increasing the water consumption per fruit produced [51]. It should be pointed out that the water use efficiency in this study was computed considering the total fruit production; so the water use efficiency of Camarosa considering the production of marketable fruits would possibly be smaller due to its higher weight of noncommercial fruits produced compared to Albion (Additional file 1: Figs. 1.3).

### Future perspectives

In our experiment with strawberries where 22 phenological, physiological, productive, and qualitative traits were assessed in 16 treatments, we were able to rank and identify the strengths and weaknesses of the treatments (Figs. 2 and 4). It was noticed that the Albion cultivar presents interesting quality features, but efforts should be focused on increasing its fruit production as well as its water use efficiency in burnt rice husk-based substrates. This would be achieved by (i) increasing the uptake efficiency of available water through the plant system, e.g., by improving root volume and surface area [38]; (ii) improving crop transpiration efficiency by acquiring more carbon per water transpired, e.g., by improving the control of stomatal closure aiming at a positive impact on daily vapor pressure deficit [52]; and (iii) increasing the partitioning of the acquired biomass into the harvested product, e.g., by a better understanding of patterns of plant biomass partitioning depending on nitrogen source [53].

The MGIDI can be applied to every situation where rows (e.g., treatments) need to be ranked based on desired values of multiple columns (traits). An application example to a published paper [8] shows how strawberries cultivars would be easily ranked based on Polyphenol, sugar, organic acid, volatile compounds, and antioxidant capacity (for all traits it was assumed that higher values are better). The computed MGIDI ranked the cultivar “Rondo” as the better cultivar based on the 27 studied traits (Additional file 1: Figure S4), which matches the results that the authors presented in the discussion of their three, 6-columns tables. In this context, the MGIDI will dramatically reduce the number of tables/figures needed, making easier the practical recommendation of superior treatments. Thus, the frontier of the MGIDI in the evaluation of several types of plant experiments is expected to rapidly expand. The implementation of the method in further studies is facilitated by its implementation in free and open-source software [26]. In Additional file 1: Supplementary Code 4 (http://bit.ly/site-mgidi-pm), we provide the data and necessary scripts to compute the index, which can be easily adapted.

### Challenges, advantages, and limitations

The key point underlying MGIDI use is choosing an *ideal* treatment. Researchers should then identify a set of key traits to be used, which will certainly vary depending on the goal of the study [19]. Compared to other multivariate techniques such as the PCA and linear indexes, MGIDI has the advantage of an easy process of treatment ranking, the strengths and weaknesses view (Fig. 2b), and the option to include weights for each trait in treatment ranking (Table 3). Compared to PCA, MGIDI has a limited graphical interface to relate the traits to treatments. Therefore, combining MGIDI and PCA would be an interesting alternative for future studies. Regarding processing time, MGIDI takes a longer time compared to PCA, but the elapsed time will be not a limitation to implementing the index. For example, for computing the index of a dataset with 150 treatments and 50 traits, less than four seconds were needed (Additional file 1: Supplementary code 5). Unfortunately, a key limitation of the method comes from one of its greatest advantages, i.e., taking into account the correlation structure of the data. Since factor analysis tends to group both positively and negatively tightly correlated variables into the same factor [54], it will be difficult to select two traits in opposite selection gains (i.e., to reduce one and increase the other) when they are positively correlated. Then, a further investigation focused on addressing this limitation would benefit and make the method more consistent and useful.

## Conclusions

Using the multi-trait genotype-ideotype distance index (MGIDI), we have shown how to select superior treatments in plant experiments where multiple traits have been assessed. In our example with strawberry data, the treatments selected by the MGIDI were characterized by the Albion cultivar originating from imported transplants grown in substrates where the main component (70%) is burnt rice husk. The selected treatments provided desired values for most out of the 22 qualitative, physiological, and phenological traits. The *strengths and weakness view* provided by the MGIDI revealed that researchers should direct efforts on increasing fruit production, improving the water use efficiency, and reducing the total acidy of fruits from the Albion Cultivar originated from Imported transplants. The strengths of this treatment are mainly related to productive precocity, total soluble solids, and flesh firmness. Overall, this study opens the door to the use of MGIDI to analyze plant multivariate data, standing out as a powerful tool to develop better recommendation strategies. The option of including weights for each trait in the selection process will make the index more applicable and provide better treatment recommendations based on several accessed traits. Improving the graphical pipeline and divergent selection for positively correlated traits are key steps to make the method more applicable.

## Supplementary Information

**Additional file 1.** A website with the data, script, and results is available at https://tiagoolivoto.github.io/paper_mgidi_pm/. The source code used to produce the static website and the results in this manuscript have been archived at 10.5281/zenodo.7155173 as manuscript v2.

## Data Availability

The raw dataset used in this study can be found in the Mendeley repository available at https://doi.org/10.17632/mcy2b3jbf5.1.

## References

[1] Meira D, Meier C, Olivoto T, Follmann DN, Rigatti A, Lunkes A, Marchioro VS, Souza VQ (2019). Multivariate analysis revealed genetic divergence and promising traits for indirect selection in black oat. Revista Brasileira de Ciências Agrárias Braz J Agric Sci.

[2] Meier C, Meira D, Marchioro VS, Olivoto T, Klein LA, Souza VQD (2019). Selection gain and interrelations between agronomic traits in wheat F5 genotypes. Revista Ceres.

[3] Follmann DN, Filho AC, Santos MSD, Costa VO, Plautz EN, Scopel JVF, Bamberg DM, Engel GH, Olivoto T, Wartha CA, Nardino M (2019). Correlations and path analysis in sunflower grown at lower elevations. J Agric Sci.

[4] Zuo Q, Wang L, Zheng J, You J, Yang G, Leng S, Liu J (2020). Effects of uniconazole rate on agronomic traits and physiological indexes of rapeseed blanket seedling. Oil Crop Sci.

[5] Dorairaj D, Ismail MR, Sinniah UR, Tan KB (2020). Silicon mediated improvement in agronomic traits, physiological parameters and fiber content in Oryza sativa. Acta Physiol Plant.

[6] Tucak M, Čupić T, Horvat D, Popović S, Krizmanić G, Ravlić M (2020). Variation of phytoestrogen content and major agronomic traits in Alfalfa (Medicago sativa L.) populations. Agronomy.

[7] Aaby K, Mazur S, Nes A, Skrede G (2012). Phenolic compounds in strawberry (Fragaria x ananassa Duch.) fruits: composition in 27 cultivars and changes during ripening. Food Chem.

[8] Urün I, Attar SH, Sönmez DA, Gündeşli MA, Ercişli S, Kafkas NE, Bandić LM, Duralija B (2021). Comparison of polyphenol, sugar, organic acid, volatile compounds, and antioxidant capacity of commercially grown strawberry cultivars in Turkey. Plants.

[9] Diel MI, Lúcio AD, Sari BG, Olivoto T, Pinheiro MVM, Krysczum DK, Melo PJD, Schmidt D (2020). Behavior of strawberry production with growth models: a multivariate approach. Acta Scientiarum Agronomy.

[10] Adak N, Heybeli N, Ertekin C (2017). Infrared drying of strawberry. Food Chem.

[11] Diel MI, Pinheiro MVM, Thiesen LA, Altíssimo BS, Holz E, Schmidt D (2018). Cultivation of strawberry in substrate: productivity and fruit quality are affected by the cultivar origin and substrates. Ciência e Agrotecnologia.

[12] Gaston A, Osorio S, Denoyes B, Rothan C (2020). Applying the Solanaceae strategies to strawberry crop improvement. Trends Plant Sci.

[13] Rahman MM, Moniruzzaman M, Ahmad MR, Sarker BC, Khurshid Alam M (2016). Maturity stages affect the postharvest quality and shelf-life of fruits of strawberry genotypes growing in subtropical regions. J Saudi Soc Agric Sci.

[14] Aamer M, Anwar MR, Mustafa G, Sohail M. Principal Component Analysis (PCA) of some morphological and quality traits in sugarcane (Saccharum officinarum L.). 2018;5.

[15] Ekka A, Tirkey A, Kujur N (2021). Cluster and principal component analysis (PCA) in Ashwagandha [Withania somnifera (L.) Dunal] for root traits. Int J Chem Stud..

[16] Urün I, Attar SH, Sönmez DA, Gündeşli MA, Ercişli S, Kafkas NE, Bandić LM, Duralija B (2021). Comparison of polyphenol, sugar, organic acid, volatile compounds, and antioxidant capacity of commercially grown strawberry cultivars in Turkey. Plants.

[17] Cerón-Rojas JJ, Crossa J (2022). The statistical theory of linear selection indices from phenotypic to genomic selection. Crop Sci.

[18] Silva LA, Peixoto MA, Peixoto LDA, Romero JV, Bhering LL (2021). Multi-trait genomic selection indexes applied to identification of superior genotypes. Bragantia.

[19] Olivoto T, Nardino M (2021). MGIDI: toward an effective multivariate selection in biological experiments. Bioinformatics.

[20] Uddin MS, Billah M, Afroz R, Rahman S, Jahan N, Hossain MG, Bagum SA, Uddin MS, Khaldun ABM, Azam MG, Hossain N, Akanda MAL, Alhomrani M, Gaber A, Hossain A (2021). Evaluation of 130 Eggplant (*Solanum melongena L.*) genotypes for future breeding program based on qualitative and quantitative traits, and various genetic parameters. Horticulturae.

[21] León R, Rosero A, García JL, Morelo J, Orozco A, Silva G, De la Ossa V, Correa E, Cordero C, Villalba L, Belalcazar J, Ceballos H (2021). Multi-trait selection indices for identifying new cassava varieties adapted to the Caribbean region of Colombia. Agronomy.

[22] Pour-Aboughadareh A, Sanjani S, Nikkhah-Chamanabad H, Mehrvar MR, Asadi A, Amini A (2021). Identification of salt-tolerant barley genotypes using multiple-traits index and yield performance at the early growth and maturity stages. Bull Natl Res Centre.

[23] Farhad M, Tripathi SB, Singh RP, Joshi AK, Bhati PK, Vishwakarma MK, Mondal S, Malik AA, Kumar U (2022). Multi-trait selection of bread wheat ideotypes for adaptation to early sown condition. Crop Sci..

[24] Osuna-Caballero S, Rispail N, Barilli E, Rubiales D (2022). Identification and characterization of novel sources of resistance to rust caused by Uromyces pisi in Pisum spp. Plants.

[25] R Core Team: R: a language and environment for statistical computing. R Foundation for Statistical Computing, Vienna, Austria. 2022. https://www.r-project.org/.

[26] Olivoto T, Lúcio AD (2020). metan: an R package for multi-environment trial analysis. Methods Ecol Evol.

[27] Russell VL. emmeans: Estimated Marginal Means, aka Least-Squares Means (2021). https://cran.r-project.org/package=emmeans.

[28] Alvares CA, Stape JL, Sentelhas PC, de Moraes Gonçalves JL, Sparovek G (2013). Köppen’s climate classification map for Brazil. Meteorol Z.

[29] Gonçalves MA, Vignolo GK, Antunes LEC, Junior CR. Produção de Morango Fora do Solo. Embrapa Clima Temperado. Pages: 34 Place: Pelotas, RS. 2016.

[30] Hand DJ, Taylor CC. Multivariate analysis of variance and repeated measures. 1987. 10.1007/978-94-009-3143-5.

[31] Olivoto T, Lúcio ADC, Silva JAG, Sari BG, Diel MI (2019). Mean performance and stability in multi-environment trials II: selection based on multiple traits. Agron J.

[32] Kaiser HF (1958). The varimax criterion for analytic rotation in factor analysis. Psychometrika.

[33] Yeomans KA, Golder PA (1982). The Guttman-Kaiser criterion as a predictor of the number of common factors. The Statistician.

[34] Zanin DS, Fagherazzi AF, Santos AMD, Martins R, Kretzschmar AA, Rufato L (2019). Agronomic performance of cultivars and advanced selections of strawberry in the South Plateau of Santa Catarina State. Rev Ceres.

[35] Wickham H, Averick M, Bryan J, Chang W, McGowan L, François R, Grolemund G, Hayes A, Henry L, Hester J, Kuhn M, Pedersen T, Miller E, Bache S, Müller K, Ooms J, Robinson D, Seidel D, Spinu V, Takahashi K, Vaughan D, Wilke C, Woo K, Yutani H (2019). Welcome to the Tidyverse. J Open Source Softw.

[36] Gabriel A, de Resende JTV, Zeist AR, Resende LV, Resende NCV, Zeist RA (2019). Phenotypic stability of strawberry cultivars based on physicochemical traits of fruits. Hortic Bras.

[37] Diel MI, Pinheiro MVM, Cocco C, Fontana DC, Caron BO, de Paula GM, Pretto MM, Thiesen LA, Schmidt D (2017). Phyllochron and phenology of strawberry cultivars from different origins cultivated in organic substracts. Sci Hortic.

[38] Costa RC, Oliveira Calvete E, dos Santos Trentin N, Trevizan Chiomento JL, Stockmans De Nardi F (2019). Characterization of external morphanatomy of the strawberry identifies new structure. Sci Hortic.

[39] Ledesma NA, Kawabata S (2016). Responses of two strawberry cultivars to severe high temperature stress at different flower development stages. Sci Hortic.

[40] Fu X, Cheng S, Zhang Y, Du B, Feng C, Zhou Y, Mei X, Jiang Y, Duan X, Yang Z (2017). Differential responses of four biosynthetic pathways of aroma compounds in postharvest strawberry (Fragaria$$\times $$ananassa Duch.) under interaction of light and temperature. Food Chem.

[41] Sønsteby A, Solhaug KA, Heide OM (2016). Functional growth analysis of ‘Sonata’ strawberry plants grown under controlled temperature and daylength conditions. Sci Hortic.

[42] Martínez F, Oliveira JA, Calvete EO, Palencia P (2017). Influence of growth medium on yield, quality indexes and SPAD values in strawberry plants. Sci Hortic.

[43] Habibzadeh F, Hazrati S, Gholamhoseini M, Khodaei D, Habashi D (2019). Evaluation of quantitative and qualitative characteristics of strawberry in response to bio- and chemical fertilizers. Gesunde Pflanzen.

[44] Sadowska A, Świderski F, Hallmann E (2020). Bioactive, physicochemical and sensory properties as well as microstructure of organic strawberry powders obtained by various drying methods. Appl Sci.

[45] Bieniasz M, Małodobry M, Dziedzic E (2012). The effect of foliar fertilization with calcium on quality of strawberry cultivars ‘Luna’ and ‘Zanta’. Acta Hortic.

[46] Silva MS, Dias MSC, Pacheco DD (2015). Desempenho produtivo e qualidade de frutos de morangueiros produzidos no norte de Minas Gerais. Hortic Bras.

[47] Forney CF, Kalt W, Jordan MA (2000). The composition of strawberry aroma is influenced by cultivar, maturity, and storage. HortScience.

[48] Wang J, Yue C, Gallardo K, McCracken V, Luby J, McFerson J (2017). What consumers are looking for in strawberries: implications from market segmentation analysis. Agribusiness.

[49] Castoldi da Costa R, Calvete EO, Spengler NCL, Chiomento JLT, Trentin NDS, De Paula JEC (2020). Morpho-phenological and agronomic performance of strawberry cultivars with different photoperiodic flowering responses. Acta Scientiarum Agronomy.

[50] Terra SB, Ferreira AAF, Peil RMN, Stumpf ERT, Beckmann-Cavalcante MZ, Cavalcante ÍHL (2011). Alternative substrates for growth and production of potted chrysanthemum (cv. Funny). Acta Scientiarum Agronomy.

[51] Martínez-Ferri E, Soria C, Ariza MT, Medina JJ, Miranda L, Domíguez P, Muriel JL (2016). Water relations, growth and physiological response of seven strawberry cultivars (Fragaria$$\times $$ananassa Duch.) to different water availability. Agric Water Manag.

[52] Sinclair TR  (2018). Effective water use required for improving crop growth rather than transpiration efficiency. Front Plant Sci.

[53] Cambui CA, Svennerstam H, Gruffman L, Nordin A, Ganeteg U, Näsholm T (2011). Patterns of plant biomass partitioning depend on Nitrogen source. PLoS ONE.

[54] Joliffe I, Morgan B (1992). Principal component analysis and exploratory factor analysis. Stat Methods Med Res.

