# Supplementary material for: MGIDI: a powerful tool to analyze plant multivariate data
Source: Plant Methods. 2022 Nov 12;18:121. doi: 10.1186/s13007-022-00952-5 (PMC9652799; doi:10.1186/s13007-022-00952-5)
Supplement: Supplementary file 1 — Additional file 1. A website with the data, script, and results is available at https://tiagoolivoto.github.io/paper_mgidi_pm/. The source code used to produce the static website and the results in this manuscript have been archived at 10.5281/zenodo.7155173 as manuscript v2. [file 13007_2022_952_MOESM1_ESM.zip › TiagoOlivoto-paper_mgidi_pm-11ef6c1/docs/code.html]

Analysis


MGIDI Plant Methods

- About
- Sup. Codes
- Sup. Figures
- Sup. Tables
- Code and data
- Doi

- metan

# Analysis

## Analysis

- 1 Libraries
- 2 Simulated data
  - 2.1 A simple example
    - 2.1.1 Pairwise multiple comparisons
    - 2.1.2 The MGIDI index
  - 2.2 A complex example
    - 2.2.1 Pairwise multiple comparisons
    - 2.2.2 The MGIDI index
- 3 Weigths
- 4 Real data example (Strawberry)
  - 4.1 Multivariate Analysis of Variance
  - 4.2 Origin x cultivar interaction
    - 4.2.1 Plot the MGIDI index
    - 4.2.2 The strengths and weaknesses view
    - 4.2.3 PCA
    - 4.2.4 Selection differentials
  - 4.3 Substrate main factor
    - 4.3.1 Plot the MGIDI index
    - 4.3.2 The strengths and weaknesses view
    - 4.3.3 PCA
    - 4.3.4 Selection differentials
- 5 Benchmark

# 1 Libraries

To reproduce the examples of this material, the R packages `metan`, `rio`, `broom`, and `purrr` are needed.

```
library(metan)      # MGIDI index
library(rio)        # data importation
library(broom)      # convert statistical objects into tidy tibbles
library(purrr)      # Manipulate lists
library(tidyverse)  # plots, data manipulation
library(kableExtra) # html tables
library(emmeans)    # pairwise multiple comparison
library(AgroR)      # pairwise multiple comparison
library(factoextra)

# set a theme plot
my_theme <- 
  theme_bw() +
  theme(legend.title = element_blank(),
        axis.title = element_text(color = "black"),
        axis.text = element_text(color = "black"),
        panel.grid.minor = element_blank())
```

# 2 Simulated data

## 2.1 A simple example

Here, we use a simple example with only 5 treatments and 3 traits to show how the MGIDI index can be used even with few traits/treatments.

```
df <- 
  g_simula(ngen = 5,
           nrep = 3,
           nvars = 3,
           seed = c(3, 20, 40)) %>% 
  replace_string(GEN, pattern =  "H", replacement = "T")
## Warning: 'gen_eff = 20' recycled for all the 3 traits.
## Warning: 'rep_eff = 5' recycled for all the 3 traits.
## Warning: 'res_eff = 5' recycled for all the 3 traits.
## Warning: 'intercept = 100' recycled for all the 3 traits.
```

### 2.1.1 Pairwise multiple comparisons

```
v1_mod <- aov(V1 ~ REP + GEN, data = df)
v2_mod <- aov(V2 ~ REP + GEN, data = df)
v3_mod <- aov(V3 ~ REP + GEN, data = df)

anovas <- 
  list(v1 = tidy(v1_mod),
       v2 = tidy(v2_mod),
       v3 = tidy(v3_mod)) %>% 
  rbind_fill_id(.id = "Traits")
anovas
```

```
v1_means <- emmeans(v1_mod, ~ GEN)
v2_means <- emmeans(v2_mod, ~ GEN)
v3_means <- emmeans(v3_mod, ~ GEN)

# plots
p1 <-
  plot(v1_means,
       comparisons = TRUE,
       CIs = FALSE,
       xlab = "Mean",
       ylab = "Treatments") + 
  geom_vline(xintercept = mean(df$V1)) +
  my_theme

p2 <- 
  plot(v2_means,
       comparisons = TRUE,
       CIs = FALSE,
       xlab = "Mean",
       ylab = "Treatments") +
  geom_vline(xintercept = mean(df$V2)) +
  my_theme

p3 <- 
  plot(v3_means,
       comparisons = TRUE,
       CIs = FALSE,
       xlab = "Mean",
       ylab = "Treatments") +
  geom_vline(xintercept = mean(df$V2)) +
  my_theme
```

### 2.1.2 The MGIDI index

The MGIDI index is computed with the function `mgidi()`. The function has the following arguments.

- `.data`: An object fitted with the function `gafem()`, `gamem()` or a two-way table with BLUPs/means for treatments in each trait (treatments in rows and traits in columns). In the last case, row names must contain the treatments’ names.
- `use_data`: Define which data to use if `.data` is an object of class `gamem`. Defaults to `"blup"` (the BLUPs for genotypes). Use `"pheno"` to use phenotypic means instead BLUPs for computing the index.
- `SI`: An integer (0-100). The selection intensity in percentage of the total number of genotypes/treatments.
- `mineval`: The minimum value so that an eigenvector is retained in the factor analysis. Defaults to 1.
- `ideotype`: A vector of length nvar where nvar is the number of variables used to plan the ideotype. Use `'h'` to indicate the traits in which higher values are desired or `'l'` to indicate the variables in which lower values are desired. For example, `ideotype = c("h, h, h, h, l")` will consider that the ideotype has higher values for the first four traits (following the order of columns in `.data`) and lower values for the last trait. If `.data` is a model fitted with the functions `gafem()` or `gamem()`, the order of the traits will be the declared in the argument `resp` in those functions.
- `weights` Optional weights to assign for each trait in the selection process. It must be a numeric vector of length equal to the number of traits in `.data`. By default (NULL) a numeric vector of weights equal to 1 is used, i.e., all traits have the same weight in the selection process. It is suggested weights ranging from 0 to 1. The weights will then shrink the ideotype vector toward 0. This is useful, for example, to prioritize grain yield rather than a plant-related trait in the selection process.
- `use`: The method for computing covariances in the presence of missing values. Defaults to `complete.obs`, i.e., missing values are handled by casewise deletion.
- `verbose`: If `verbose = TRUE` (Default) then some results are shown in the console.

In the following example, the MGIDI index is computed with the predicted values from a multivariate analysis of variance model. Three scenarios are shown: (i) for all traits higher values are better; (ii) lower values better for the first two traits and higher values better for the last trait; and (iii) lower values better for all the traits.

```
man <- manova(cbind(V1, V2, V3) ~ REP + GEN, data = df)
summary(man)
##           Df Pillai approx F num Df den Df    Pr(>F)    
## REP        2 1.3052   4.3832      6     14   0.01068 *  
## GEN        4 2.6178  13.6999     12     24 6.053e-08 ***
## Residuals  8                                            
## ---
## Signif. codes:  0 '***' 0.001 '**' 0.01 '*' 0.05 '.' 0.1 ' ' 1

# predict the mean values
# compute a two-way table with genotypes in rows and traits in columns

pred_mat <- 
  predict(man) %>% 
  as.data.frame() %>% 
  mutate(TRAT = df$GEN) %>% 
  mean_by(TRAT) %>% 
  column_to_rownames("TRAT")


# higher values for all traits are better
ind <- mgidi(pred_mat,
             ideotype = c("h, h, h"), # default
             verbose = FALSE,
             mineval = 0.5) # retains two factors
p4 <-
  plot(ind,
       x.lab = "Treatments",
       y.lab = "MGIDI index",
       radar = FALSE) +
  my_theme
ind$sel_dif
```

```
# lower values for the first two traits
# higher values for the last 
ind2 <- mgidi(pred_mat,
              ideotype = c("l, l, h"),
              verbose = FALSE,
              mineval = 0.5) # retains two factors
p5 <- 
  plot(ind2,
       x.lab = "Treatments",
       y.lab = "MGIDI index",
       radar = FALSE) +
  my_theme
ind2$sel_dif
```

```
# lower values for all the traits
ind3 <- mgidi(pred_mat,
              ideotype =  c("l, l, l"),
              verbose = FALSE,
              mineval = 0.5) # retains two factors
p6 <-
  plot(ind3,
       x.lab = "Treatments",
       y.lab = "MGIDI index",
       radar = FALSE) +
  my_theme
ind3$sel_dif
```

```
arrange_ggplot((p1 + p2 + p3),
               (p4 + p5 + p6),
               nrow = 2,
               heights = c(0.6, 1),
               tag_levels = "a",
               guides = "collect")
```

Figure 2.1: Pairwise comparisons for V1 (a), V2 (b), V3 (c), and the MGIDI index for three selection strategies, namely, desired higher values for all traits (c), lower values for V1 and V2 and higher values for V3 (d), and lower values for all traits (e).

```
ggsave("figs/fig1.pdf", width = 10, height = 5)
```

## 2.2 A complex example

### 2.2.1 Pairwise multiple comparisons

In the following example, we will simulate data on 10 traits accessed in 75 treatments using the function `g_simula()` from `metan` package.

```
# simulate data
df_g <-
  g_simula(ngen = 75,
           nrep = 3,
           nvars = 10,
           seed = 1:10)
## Warning: 'gen_eff = 20' recycled for all the 10 traits.
## Warning: 'rep_eff = 5' recycled for all the 10 traits.
## Warning: 'res_eff = 5' recycled for all the 10 traits.
## Warning: 'intercept = 100' recycled for all the 10 traits.

# data in a 'long' format
df_ge_long <-
  pivot_longer(df_g,
               cols = -c(GEN:REP),
               names_to = "trait")
# compute an ANOVA for each trait
models_ge <-
  df_ge_long %>%
  group_by(trait) %>%
  doo(~aov(value ~ REP + GEN, data = .))

# obtain an anova table
anovas_ge <- models_ge$data %>% map_dfr(~.x %>% tidy(), .id = "TRAIT")
anovas_ge %>% filter(term == "GEN")
```

All traits with significant treatment effect. In this case, It would be reasonable to include a pairwise comparasion. We could obtain all the P-values using `pwpm()`, and look at each one of the 2775 (\(75!/ (2! \times (75 - 2)!\)) comparisions individually.

```
# example for V1
model_v1 <- models_ge$data[[1]]
means_v1 <- emmeans(model_v1, ~ GEN)
pairs <- pwpm(means_v1)
```

Definetively, this is not the better option. Another way to depict comparisons graphically via the comparisons argument in `plot.emm()`:

```
p7 <- 
  plot(means_v1, comparisons = TRUE, CIs = FALSE) +
  my_theme
p7
```

Figure 2.2: Pairwise comparisons based on Tukey adjustment

The red arrows are for the comparisons among genotype’s means. If an arrow from one mean overlaps an arrow from another group, the difference is not “significant,” based on the adjust setting (which defaults to “tukey”).

An alternative is the Pairwise P-value plot displaying all the P-values in pairwise comparisons. Each comparison is associated with a vertical line segment that joins the scale positions of the two means being compared, and whose horizontal position is determined by the P-value of that comparison.

```
# shows only 'significant' (P-value < 0.05)
p8 <- pwpp(means_v1) + scale_x_continuous(limits = c(0, 0.05))
## Scale for 'x' is already present. Adding another scale for 'x', which will
## replace the existing scale.
p8
## Warning: Removed 4350 rows containing missing values (geom_point).
## Warning: Removed 4350 rows containing missing values (geom_segment).
## Warning: Removed 5550 rows containing missing values (geom_point).
## Warning: Removed 75 rows containing missing values (geom_label).
## Warning: Removed 5550 rows containing missing values (geom_point).
```

Figure 2.3: Pairwise P-value plot

Another way to depict comparisons is by compact letter displays, whereby two means sharing one or more grouping symbols are not “significantly” different. Here, I use the package `ExpDes` to generate compact letter displays based on Tukey test.

```
plot <- 
  with(df_g,
       DBC(GEN, REP, V1))
## 
## -----------------------------------------------------------------
## Normality of errors
## -----------------------------------------------------------------
##                          Method Statistic   p.value
##  Shapiro-Wilk normality test(W) 0.9952492 0.7118349
## As the calculated p-value is greater than the 5% significance level, hypothesis H0 is not rejected. Therefore, errors can be considered normal
## 
## -----------------------------------------------------------------
## Homogeneity of Variances
## -----------------------------------------------------------------
##                               Method Statistic   p.value
##  Bartlett test(Bartlett's K-squared)  80.00464 0.2962211
## As the calculated p-value is greater than the 5% significance level, hypothesis H0 is not rejected. Therefore, the variances can be considered homogeneous
## 
## -----------------------------------------------------------------
## Independence from errors
## -----------------------------------------------------------------
##                  Method Statistic   p.value
##  Durbin-Watson test(DW)  2.580335 0.2531596
## As the calculated p-value is greater than the 5% significance level, hypothesis H0 is not rejected. Therefore, errors can be considered independent
## 
## -----------------------------------------------------------------
## Additional Information
## -----------------------------------------------------------------
## 
## CV (%) =  4.84
## MStrat/MST =  0.28
## Mean =  102.6011
## Median =  102.4724
## Possible outliers =  No discrepant point
## 
## -----------------------------------------------------------------
## Analysis of Variance
## -----------------------------------------------------------------
##            Df    Sum Sq   Mean.Sq  F value        Pr(F)
## trat       74 25647.458 346.58726 14.04936 1.358273e-40
## bloco       2  1702.585 851.29272 34.50824 4.999272e-13
## Residuals 148  3651.051  24.66926
## As the calculated p-value, it is less than the 5% significance level. The hypothesis H0 of equality of means is rejected. Therefore, at least two treatments differ
```

```
## 
## -----------------------------------------------------------------
## Multiple Comparison Test
## -----------------------------------------------------------------
##          resp              groups
## H18 124.87425                   a
## H6  123.16768                  ab
## H4  120.42818                 abc
## H7  118.92632                abcd
## H41 118.41918               abcde
## H21 117.79055               abcde
## H29 117.66426               abcde
## H72 116.36309              abcdef
## H68 116.08235              abcdef
## H61 115.71047             abcdefg
## H70 115.55697            abcdefgh
## H52 114.75437           abcdefghi
## H46 114.42753           abcdefghi
## H43 114.35004          abcdefghij
## H23 113.87899         abcdefghijk
## H8  113.67386         abcdefghijk
## H35 113.66349         abcdefghijk
## H15 112.14765        abcdefghijkl
## H17 111.41472       abcdefghijklm
## H59 110.53092      abcdefghijklmn
## H50 110.31151     abcdefghijklmno
## H42 110.24694     abcdefghijklmno
## H49 109.60600    abcdefghijklmnop
## H13 109.38477    abcdefghijklmnop
## H36 109.26114    abcdefghijklmnop
## H37 109.07550    abcdefghijklmnop
## H44 108.22841    abcdefghijklmnop
## H20 108.12878    abcdefghijklmnop
## H9  106.44219    bcdefghijklmnopq
## H3  105.77859     cdefghijklmnopq
## H16 105.37972    cdefghijklmnopqr
## H39 105.28350    cdefghijklmnopqr
## H32 105.00382   cdefghijklmnopqrs
## H45 104.15329  cdefghijklmnopqrst
## H65 103.25842 cdefghijklmnopqrstu
## H58 102.66058  defghijklmnopqrstu
## H19 102.09905 defghijklmnopqrstuv
## H75 101.51554 efghijklmnopqrstuvw
## H48 101.19846 efghijklmnopqrstuvw
## H67 101.13853 efghijklmnopqrstuvw
## H63 100.29521  fghijklmnopqrstuvw
## H51 100.09121 fghijklmnopqrstuvwx
## H31 100.01662 fghijklmnopqrstuvwx
## H53  98.64119 ghijklmnopqrstuvwxy
## H28  98.48811 ghijklmnopqrstuvwxy
## H71  98.32443  hijklmnopqrstuvwxy
## H2   97.98150   ijklmnopqrstuvwxy
## H33  97.95390   ijklmnopqrstuvwxy
## H74  97.00687    jklmnopqrstuvwxy
## H40  96.92996     klmnopqrstuvwxy
## H60  96.89532     klmnopqrstuvwxy
## H57  96.53602     klmnopqrstuvwxy
## H26  96.06602      lmnopqrstuvwxy
## H14  95.71283      lmnopqrstuvwxy
## H73  94.88222      lmnopqrstuvwxy
## H64  94.57512       mnopqrstuvwxy
## H30  94.44762       mnopqrstuvwxy
## H11  93.62437        nopqrstuvwxy
## H25  93.37715        nopqrstuvwxy
## H1   93.35092        nopqrstuvwxy
## H66  93.14729         opqrstuvwxy
## H54  92.68082          pqrstuvwxy
## H62  90.35282           qrstuvwxy
## H47  90.27258           qrstuvwxy
## H55  89.88984           qrstuvwxy
## H24  89.56167           qrstuvwxy
## H22  88.29500            rstuvwxy
## H5   87.88859             stuvwxy
## H56  87.59956              tuvwxy
## H38  86.66210               uvwxy
## H12  86.58902               uvwxy
## H34  85.23154                vwxy
## H10  84.31629                 wxy
## H27  82.75361                  xy
## H69  82.66512                   y
```

Now, we simply choose one of the above options and apply it to the other nine traits.

### 2.2.2 The MGIDI index

In the following example, the MGIDI index is computed with the predicted values from a multivariate analysis of variance model. Three scenarios are shown: (i) for all traits higher values are better; (ii) higher values better for the first seven traits and lower values better for the last three traits; and (iii) lower values better for all the traits.

```
man_complex <- 
  manova(cbind(V1,V2,V3,V4,V5,V6,V7,V8,V9,V10) ~ REP + GEN,
         data = df_g)
summary(man_complex)
##            Df Pillai approx F num Df den Df    Pr(>F)    
## REP         2 1.3483   28.967     20    280 < 2.2e-16 ***
## GEN        74 8.8813   15.878    740   1480 < 2.2e-16 ***
## Residuals 148                                            
## ---
## Signif. codes:  0 '***' 0.001 '**' 0.01 '*' 0.05 '.' 0.1 ' ' 1


# generate a two-way table with predicted values in rows and traits in columns
pred_complex <- cbind(df_g[, 1:2], predict(man_complex))
mat_g <-
  pred_complex %>%
  mean_by(GEN) %>%
  column_to_rownames("GEN")

# higher values for all traits are better
ind4 <- mgidi(mat_g,
              ideotype = rep("h", 10), # default
              verbose = FALSE)
p9 <- plot(ind4)
p9
```

```
ind4$sel_dif
```

```
# higher values desired for the first 7 traits
# lower values desired for the last 3 traits
ind5 <- mgidi(mat_g,
              ideotype = c(rep("h", 7), rep("l", 3)),
              verbose = FALSE)
p10 <- plot(ind5)
p10
```

```
ind5$sel_dif
```

```
# lower values for all the traits
ind6 <- mgidi(mat_g,
              ideotype =  rep("l", 10), # default
              verbose = FALSE)
p11 <- plot(ind6)
p11
```

```
ind6$sel_dif
```

# 3 Weigths

```
df <- 
  g_simula(ngen = 5,
           nrep = 3,
           nvars = 3,
           seed = c(5, 2, 32)) %>% 
  mean_by(GEN) |> 
  column_to_rownames("GEN")
## Warning: 'gen_eff = 20' recycled for all the 3 traits.
## Warning: 'rep_eff = 5' recycled for all the 3 traits.
## Warning: 'res_eff = 5' recycled for all the 3 traits.
## Warning: 'intercept = 100' recycled for all the 3 traits.

# equal weights
equal_weigths <- mgidi(df, verbose = FALSE)
df
```

```
equal_weigths$scores_ide
```

```
equal_weigths$scores_gen
```

```
equal_weigths$MGIDI
```

```
# higuer weight for V2 
# see how H5 becomes the first ranked
wv2 <- mgidi(df,
             weights = c(1, 15, 1),
             verbose = FALSE)
df
```

```
wv2$scores_ide
```

```
wv2$scores_gen
```

```
wv2$MGIDI
```

# 4 Real data example (Strawberry)

```
rm(list = ls())
# set a theme plot
my_theme <- 
  theme_bw() +
  theme(legend.title = element_blank(),
        axis.title = element_text(color = "black"),
        axis.text = element_text(color = "black"),
        panel.grid.minor = element_blank())

df <-
  import("https://bit.ly/strawberry_data", setclass = "tbl") %>% 
  metan::as_factor(1:4)
str(df)
## tibble [64 × 26] (S3: tbl_df/tbl/data.frame)
##  $ REP   : Factor w/ 4 levels "1","2","3","4": 1 1 1 1 1 1 1 1 1 1 ...
##  $ SUB   : Factor w/ 4 levels "S1","S2","S3",..: 1 1 1 1 2 2 2 2 3 3 ...
##  $ CUL   : Factor w/ 2 levels "ALB","CAM": 2 2 1 1 2 2 1 1 2 2 ...
##  $ ORI   : Factor w/ 2 levels "IMP","NAC": 2 1 2 1 2 1 2 1 2 1 ...
##  $ NNCF  : num [1:64] 3.88 6.13 1.2 2.86 4.67 ...
##  $ WNCF  : num [1:64] 21.4 36.2 10.3 50.1 41.1 ...
##  $ AWNCF : num [1:64] 5.51 5.92 8.62 17.55 8.81 ...
##  $ WUE   : num [1:64] 93.4 125.5 136.3 94.2 190.5 ...
##  $ NDBF  : int [1:64] 82 61 69 44 86 61 71 44 54 55 ...
##  $ NDFF  : int [1:64] 92 83 82 70 92 89 82 70 69 70 ...
##  $ NDBH  : int [1:64] 81 82 78 69 108 85 88 72 74 79 ...
##  $ PHYL  : num [1:64] 162 185 189 180 124 ...
##  $ TA    : num [1:64] 1.38 1.72 1.77 1.4 1.51 1.34 1.47 1.49 1.33 1.6 ...
##  $ NCF   : num [1:64] 28.1 24 19.6 24.9 12.3 ...
##  $ TNF   : num [1:64] 32 30.1 20.8 27.7 17 ...
##  $ WCF   : num [1:64] 425 307 330 372 148 ...
##  $ TWF   : num [1:64] 446 343 340 423 189 ...
##  $ AWCF  : num [1:64] 15.1 12.8 16.8 15 12 ...
##  $ OAWF  : num [1:64] 13.9 11.4 16.4 15.2 11.1 ...
##  $ FY    : num [1:64] 33469 25713 25509 31698 14170 ...
##  $ TSS   : num [1:64] 6.77 8.27 7.25 8.35 7.2 7.13 7.45 8.4 6.8 7.88 ...
##  $ TSS_TA: num [1:64] 4.89 4.79 4.1 5.98 4.76 5.34 5.08 5.64 5.13 4.92 ...
##  $ FIRM  : num [1:64] 1.53 1.95 2.07 1.93 1.73 1.92 1.71 1.99 0.95 1.69 ...
##  $ L     : num [1:64] 57 48.7 55.2 53.2 53.4 ...
##  $ CHROMA: num [1:64] 40.5 47.1 46 47 43.7 ...
##  $ H     : num [1:64] 42.4 40.7 43.2 42.7 42.2 ...
```

## 4.1 Multivariate Analysis of Variance

The function `manova()` computes a multivariate analysis of variance `df`.

```
man_straw <- 
  manova(
    cbind(NNCF,WNCF,AWNCF,WUE,NDBF,NDFF,NDBH,PHYL,TA,NCF,TNF
          ,WCF, TWF,AWCF,OAWF,FY,TSS,TSS_TA,FIRM,L,CHROMA,H) ~ SUB*ORI*CUL,
    data = df)
tidy(man_straw, intercept =  TRUE)
```

Since the only significant interaction was ORI:CUL, we will use the MGIDI index to analyze the interaction term and the main effect of substrat. First, the predicted values are obtained with `predict()`.

```
# get the predicted values
pred_vals <- cbind(df[,1:4], predict(man_straw))
```

## 4.2 Origin x cultivar interaction

The MGIDI index can be computed either using a model of class `gamem` or `gafem`, or by using a two-way table. In our example, we create a two-way table with the predicted values for each treatment (factor combinations) in rows and traits in columns.

```
# compute a two-wat table with means by origin and cultivar
df_ori_cul <-
  pred_vals %>%
  mean_by(ORI, CUL) %>% 
  concatenate(ORI, CUL, new_var = TRAT, .after = CUL) %>% 
  remove_cols(ORI, CUL) %>% 
  round_cols() %>% 
  column_to_rownames("TRAT")

df_ori_cul
```

```
(ideotype_vector <- c(rep("l", 9), rep("h", 13)))
##  [1] "l" "l" "l" "l" "l" "l" "l" "l" "l" "h" "h" "h" "h" "h" "h" "h" "h" "h" "h"
## [20] "h" "h" "h"
(weight <-  c(rep(1, 9), 4, 1, 4, rep(1, 10)))
##  [1] 1 1 1 1 1 1 1 1 1 4 1 4 1 1 1 1 1 1 1 1 1 1

mgidi_cul_ori <- 
  mgidi(df_ori_cul,                 # a two-way table
        ideotype = ideotype_vector, # ideotype vector
        weights = weight,           # weigth vector
        SI = 50)                    # select two treatments
## 
## -------------------------------------------------------------------------------
## Principal Component Analysis
## -------------------------------------------------------------------------------
## # A tibble: 22 × 4
##    PC    Eigenvalues `Variance (%)` `Cum. variance (%)`
##    <chr>       <dbl>          <dbl>               <dbl>
##  1 PC1         14.2            64.4                64.4
##  2 PC2          5.24           23.8                88.2
##  3 PC3          2.58           11.8               100  
##  4 PC4          0               0                 100  
##  5 PC5          0               0                 100  
##  6 PC6          0               0                 100  
##  7 PC7          0               0                 100  
##  8 PC8          0               0                 100  
##  9 PC9          0               0                 100  
## 10 PC10         0               0                 100  
## # … with 12 more rows
## -------------------------------------------------------------------------------
## Factor Analysis - factorial loadings after rotation-
## -------------------------------------------------------------------------------
## # A tibble: 22 × 6
##    VAR     FA1   FA2   FA3 Communality Uniquenesses
##    <chr> <dbl> <dbl> <dbl>       <dbl>        <dbl>
##  1 NNCF  -0.72 -0.67 -0.17           1            0
##  2 WNCF  -0.65 -0.75 -0.14           1            0
##  3 AWNCF  0.63  0.49  0.6            1            0
##  4 WUE    1     0.04  0.03           1            0
##  5 NDBF  -0.11  0.07 -0.99           1            0
##  6 NDFF  -0.16 -0.24 -0.96           1            0
##  7 NDBH  -0.17  0.07 -0.98           1            0
##  8 PHYL   0.6   0.79 -0.12           1            0
##  9 TA     0.84  0.1   0.54           1            0
## 10 NCF    0.84  0.42  0.35           1            0
## # … with 12 more rows
## -------------------------------------------------------------------------------
## Comunalit Mean: 1 
## -------------------------------------------------------------------------------
## Selection differential 
## -------------------------------------------------------------------------------
## # A tibble: 22 × 8
##    VAR    Factor       Xo       Xs        SD  SDperc sense     goal
##    <chr>  <chr>     <dbl>    <dbl>     <dbl>   <dbl> <chr>    <dbl>
##  1 NNCF   FA1        7.20     7.86    0.650    9.02  decrease     0
##  2 AWNCF  FA1        8.74     8.70   -0.0425  -0.486 decrease   100
##  3 WUE    FA1      116.     101.    -15.0    -12.9   decrease   100
##  4 TA     FA1        1.42     1.35   -0.0700  -4.93  decrease   100
##  5 NCF    FA1       23.5     25.0     1.52     6.48  increase   100
##  6 TNF    FA1       30.7     32.8     2.16     7.04  increase   100
##  7 WCF    FA1      359.     395.     36.9     10.3   increase   100
##  8 TWF    FA1      413.     442.     29.1      7.05  increase   100
##  9 FY     FA1    30957.   33137.   2180.       7.04  increase   100
## 10 TSS_TA FA1        5.39     5.66    0.268    4.96  increase   100
## # … with 12 more rows
## ------------------------------------------------------------------------------
## Selected genotypes
## -------------------------------------------------------------------------------
## IMP_ALB NAC_CAM
## -------------------------------------------------------------------------------
```

### 4.2.1 Plot the MGIDI index

The radar plot with the treatment ranking based on the MGIDI index is created with the S3 method `plot`. The argument `type` controls which plot is created.

```
p_ind_cul_ori <- 
  plot(mgidi_cul_ori,
       SI = 50,
       radar = FALSE,
       x.lab = "Origin x cultivar combination",
       y.lab = "MGIDI index") +
  my_theme +
  theme(legend.title = element_blank(),
        legend.position = "bottom")
```

### 4.2.2 The strengths and weaknesses view

In the following code we obtain the contribution of each factor on the MGIDI value of all treatments. To do that, we use the argument `type = "contribution"`, and `genotypes = "all"` to show the contribution of all treatments in our case. By default, contribution plot shows only the selected treatments. in this case,

```
p_cont_cul_ori <- 
  plot(mgidi_cul_ori,
       type = "contribution", # Get the proportion plot
       genotypes = "all", # All treatments (selected treatments are plotted by default)
       title = "") +
  my_theme +
  theme(legend.position = "bottom")
```

### 4.2.3 PCA

```
pca_cul_ori <- prcomp(df_ori_cul, scale. = TRUE)
factors <- 
  mgidi_cul_ori$sel_dif |> 
  select(VAR, Factor)
factors <- 
  data.frame(VAR = names(df_ori_cul)) |> 
  left_join(factors)
## Joining, by = "VAR"

pca <- 
  fviz_pca_biplot(pca_cul_ori, 
                  repel = TRUE,
                  col.var = factors$Factor) +
  labs(color = "") +
  my_theme +
  theme(legend.position = "bottom")

# save the plot
arrange_ggplot(p_ind_cul_ori,
               p_cont_cul_ori,
               pca,
               ncol = 3,
               tag_levels = "a")
```

```
ggsave("figs/fig2.pdf", width = 12, height = 4)
```

### 4.2.4 Selection differentials

```
dif_cul_ori <- 
  gmd(mgidi_cul_ori) %>% 
  mutate(negative = ifelse(SDperc <= 0 , "Negative", "Positive")) %>% 
  mutate(sense = ifelse(sense == "decrease", "Negative desired", "Positive desired"))
## Class of the model: mgidi
## Variable extracted: sel_dif

ggplot(dif_cul_ori, aes(SDperc, VAR)) +
  geom_vline(xintercept = 0, size = 0.2) + 
  geom_col(aes(fill = negative),
           width = 1,
           col = "black",
           size = 0.2) +
  scale_x_continuous(expand = expansion(mult = 0.15)) +
  facet_wrap(~ sense, scales = "free", ncol = 1) + 
  geom_text(aes(label = round(SDperc, 2),
                hjust = ifelse(SDperc > 0, -0.1, 1.1)),
            size = 2) + 
  labs(x = "Selection differential (%)",
       y = "Traits") + 
  theme(legend.position = "bottom",
        legend.title = element_blank(),
        panel.grid.minor = element_blank()) +
  my_theme +
  theme(legend.position = "bottom")
```

```
ggsave("figs/fig3.pdf", width = 4, height = 7)
```

## 4.3 Substrate main factor

```
df_sub <- 
  pred_vals %>% 
  mean_by(SUB) %>% 
  column_to_rownames("SUB")

(weight <-  c(rep(1, 9), 4, 1, 4, rep(1, 10)))
##  [1] 1 1 1 1 1 1 1 1 1 4 1 4 1 1 1 1 1 1 1 1 1 1
mgidi_sub <-
  mgidi(df_sub,  # a two-way table
        ideotype = ideotype_vector, # ideotype vector
        weights = weight,           # weigth vector
        SI = 50) # select the top two substrates
## 
## -------------------------------------------------------------------------------
## Principal Component Analysis
## -------------------------------------------------------------------------------
## # A tibble: 22 × 4
##    PC    Eigenvalues `Variance (%)` `Cum. variance (%)`
##    <chr>       <dbl>          <dbl>               <dbl>
##  1 PC1         14.9            67.8                67.8
##  2 PC2          3.95           18.0                85.8
##  3 PC3          3.12           14.2               100  
##  4 PC4          0               0                 100  
##  5 PC5          0               0                 100  
##  6 PC6          0               0                 100  
##  7 PC7          0               0                 100  
##  8 PC8          0               0                 100  
##  9 PC9          0               0                 100  
## 10 PC10         0               0                 100  
## # … with 12 more rows
## -------------------------------------------------------------------------------
## Factor Analysis - factorial loadings after rotation-
## -------------------------------------------------------------------------------
## # A tibble: 22 × 6
##    VAR     FA1   FA2   FA3 Communality Uniquenesses
##    <chr> <dbl> <dbl> <dbl>       <dbl>        <dbl>
##  1 NNCF  -0.99  0.03 -0.17           1            0
##  2 WNCF  -0.83  0.21 -0.52           1            0
##  3 AWNCF  0.26  0.19 -0.95           1            0
##  4 WUE    0.88 -0.33 -0.35           1            0
##  5 NDBF   0.74  0.14 -0.66           1            0
##  6 NDFF   0.91 -0.33 -0.25           1            0
##  7 NDBH   0.85 -0.46 -0.27           1            0
##  8 PHYL   0.88 -0.01 -0.47           1            0
##  9 TA     0.17 -0.71 -0.69           1            0
## 10 NCF    0.96 -0.21 -0.18           1            0
## # … with 12 more rows
## -------------------------------------------------------------------------------
## Comunalit Mean: 1 
## -------------------------------------------------------------------------------
## Selection differential 
## -------------------------------------------------------------------------------
## # A tibble: 22 × 8
##    VAR   Factor     Xo     Xs      SD SDperc sense     goal
##    <chr> <chr>   <dbl>  <dbl>   <dbl>  <dbl> <chr>    <dbl>
##  1 NNCF  FA1      7.20   7.89   0.690   9.58 decrease     0
##  2 WNCF  FA1     65.4   69.3    3.97    6.07 decrease     0
##  3 WUE   FA1    116.    83.2  -32.6   -28.1  decrease   100
##  4 NDBF  FA1     57.7   53.9   -3.80   -6.58 decrease   100
##  5 NDFF  FA1     76.3   71.9   -4.36   -5.72 decrease   100
##  6 NDBH  FA1     77.8   75.5   -2.30   -2.95 decrease   100
##  7 PHYL  FA1    164.   147.   -17.4   -10.6  decrease   100
##  8 NCF   FA1     23.5   29.0    5.52   23.5  increase   100
##  9 TNF   FA1     30.7   36.9    6.20   20.2  increase   100
## 10 WCF   FA1    359.   462.   103.     28.8  increase   100
## # … with 12 more rows
## ------------------------------------------------------------------------------
## Selected genotypes
## -------------------------------------------------------------------------------
## S3 S4
## -------------------------------------------------------------------------------
```

### 4.3.1 Plot the MGIDI index

The radar plot with the treatment ranking based on the MGIDI index is created with the S3 method `plot`. The argument `type` controls which plot is created.

```
p_ind_sub <- 
  plot(mgidi_sub,
       SI = 50,
       radar = FALSE,
       x.lab = "Origin x cultivar combination",
       y.lab = "MGIDI index") +
  my_theme +
  theme(legend.position = "bottom")
```

### 4.3.2 The strengths and weaknesses view

```
p_cont_sub <- 
  plot(mgidi_sub,
       type = "contribution", # Get the proportion plot
       genotypes = "all", # All treatments (selected treatments are plotted by default)
       title = "") +
  my_theme +
  theme(legend.position = "bottom")
```

### 4.3.3 PCA

```
pca_sub <- prcomp(df_sub, scale. = TRUE)
factors <- 
  mgidi_sub$sel_dif |> 
  select(VAR, Factor)
factors <- 
  data.frame(VAR = names(df_sub)) |> 
  left_join(factors)
## Joining, by = "VAR"

pca_sub <- 
  fviz_pca_biplot(pca_sub, 
                  repel = TRUE,
                  col.var = factors$Factor) +
  labs(color = "") +
  my_theme +
  theme(legend.position = "bottom")

# save the plot
arrange_ggplot(p_ind_sub,
               p_cont_sub,
               pca_sub,
               ncol = 3,
               tag_levels = "a")
```

```
ggsave("figs/fig4.pdf", width = 12, height = 4)
```

### 4.3.4 Selection differentials

```
dif_sub <- 
  gmd(mgidi_sub) %>% 
  mutate(negative = ifelse(SDperc <= 0 , "Negative", "Positive")) %>% 
  mutate(sense = ifelse(sense == "decrease", "Negative desired", "Positive desired"))
## Class of the model: mgidi
## Variable extracted: sel_dif

ggplot(dif_sub, aes(SDperc, VAR)) +
  geom_vline(xintercept = 0, size = 0.2) + 
  geom_col(aes(fill = negative),
           width = 1,
           col = "black",
           size = 0.2) +
  scale_x_continuous(expand = expansion(mult = 0.1)) +
  facet_wrap(~ sense, scales = "free", ncol = 1) + 
  geom_text(aes(label = round(SDperc, 2),
                hjust = ifelse(SDperc > 0, -0.1, 1.1)),
            size = 2) + 
  labs(x = "Selection differential (%)",
       y = "Traits")  +
  my_theme +
  theme(legend.position = "bottom")
```

```
ggsave("figs/fig5.pdf", width = 4, height = 7)
```

# 5 Benchmark

```
df <- 
  g_simula(150, 2, 50) |> 
  mean_by(GEN) |> 
  column_to_rownames("GEN")
## Warning: 'gen_eff = 20' recycled for all the 50 traits.
## Warning: 'rep_eff = 5' recycled for all the 50 traits.
## Warning: 'res_eff = 5' recycled for all the 50 traits.
## Warning: 'intercept = 100' recycled for all the 50 traits.

system.time(
  mgidi(df)
)
## 
## -------------------------------------------------------------------------------
## Principal Component Analysis
## -------------------------------------------------------------------------------
## # A tibble: 50 × 4
##    PC    Eigenvalues `Variance (%)` `Cum. variance (%)`
##    <chr>       <dbl>          <dbl>               <dbl>
##  1 PC1          2.3            4.59                4.59
##  2 PC2          2.18           4.37                8.96
##  3 PC3          2.09           4.18               13.1 
##  4 PC4          1.99           3.99               17.1 
##  5 PC5          1.96           3.93               21.1 
##  6 PC6          1.88           3.76               24.8 
##  7 PC7          1.79           3.58               28.4 
##  8 PC8          1.77           3.54               31.9 
##  9 PC9          1.6            3.19               35.1 
## 10 PC10         1.59           3.18               38.3 
## # … with 40 more rows
## -------------------------------------------------------------------------------
## Factor Analysis - factorial loadings after rotation-
## -------------------------------------------------------------------------------
## # A tibble: 50 × 25
##    VAR     FA1   FA2   FA3   FA4   FA5   FA6   FA7   FA8   FA9  FA10  FA11  FA12
##    <chr> <dbl> <dbl> <dbl> <dbl> <dbl> <dbl> <dbl> <dbl> <dbl> <dbl> <dbl> <dbl>
##  1 V1     0.06 -0.22  0.01  0.12 -0.05 -0.03  0.45 -0.02  0     0.09  0.24  0   
##  2 V2     0.05  0.31  0.05 -0.19 -0.04  0.2  -0.07  0.1   0.04  0.02 -0.05  0.18
##  3 V3     0.03 -0.18  0.09  0.07  0.17 -0.13  0.07 -0.03 -0.18  0.04 -0.11 -0.51
##  4 V4    -0.07  0.07  0     0.06  0.01 -0.02 -0.01  0.05 -0.01  0.82  0.05  0   
##  5 V5    -0.06  0.12 -0.01 -0.09 -0.02 -0.01  0.11 -0.08 -0.03 -0.04  0.07 -0.04
##  6 V6    -0.02 -0.05  0.07  0.01 -0.03  0.01 -0.73  0.04  0.05  0.01  0.01  0.01
##  7 V7    -0.08 -0.05  0    -0.01 -0.07  0.01 -0.04  0.07 -0.03 -0.02 -0.12  0.1 
##  8 V8     0.07  0.01  0.03  0.07  0.14  0    -0.01 -0.01  0.03  0     0.1  -0.01
##  9 V9     0.04 -0.13  0.28  0.14 -0.15  0.06 -0.11  0.01 -0.16 -0.14 -0.13  0.19
## 10 V10   -0.06 -0.05  0.03  0.09  0.02  0    -0.01 -0.07 -0.11  0.02 -0.09  0.76
## # … with 40 more rows, and 12 more variables: FA13 <dbl>, FA14 <dbl>,
## #   FA15 <dbl>, FA16 <dbl>, FA17 <dbl>, FA18 <dbl>, FA19 <dbl>, FA20 <dbl>,
## #   FA21 <dbl>, FA22 <dbl>, Communality <dbl>, Uniquenesses <dbl>
## -------------------------------------------------------------------------------
## Comunalit Mean: 0.6866126 
## -------------------------------------------------------------------------------
## Selection differential 
## -------------------------------------------------------------------------------
## # A tibble: 50 × 8
##    VAR   Factor    Xo    Xs      SD  SDperc sense     goal
##    <chr> <chr>  <dbl> <dbl>   <dbl>   <dbl> <chr>    <dbl>
##  1 V19   FA1     98.7  98.9  0.253   0.256  increase   100
##  2 V20   FA1     99.9 100.   0.405   0.405  increase   100
##  3 V21   FA2    102.  107.   5.06    4.98   increase   100
##  4 V28   FA2    101.  103.   1.60    1.58   increase   100
##  5 V31   FA2     97.2  98.4  1.20    1.24   increase   100
##  6 V33   FA3    104.  111.   6.66    6.41   increase   100
##  7 V38   FA4     99.6 107.   7.09    7.12   increase   100
##  8 V46   FA4     98.6  98.6  0.0577  0.0585 increase   100
##  9 V17   FA5     98.9  96.5 -2.35   -2.38   increase     0
## 10 V44   FA5     98.4 102.   3.58    3.64   increase   100
## # … with 40 more rows
## ------------------------------------------------------------------------------
## Selected genotypes
## -------------------------------------------------------------------------------
## H14 H122 H67 H37 H34 H111 H41 H113 H36 H60 H55 H56 H32 H128 H12 H130 H87 H10 H147 H44 H51 H109
## -------------------------------------------------------------------------------
##   usuário   sistema decorrido 
##      4.11      0.05      4.15

system.time(
  prcomp(df)
)
##   usuário   sistema decorrido 
##         0         0         0
```
