# Supplementary material for: MGIDI: a powerful tool to analyze plant multivariate data
Source: Plant Methods. 2022 Nov 12;18:121. doi: 10.1186/s13007-022-00952-5 (PMC9652799; doi:10.1186/s13007-022-00952-5)
Supplement: Supplementary file 1 — Additional file 1. A website with the data, script, and results is available at https://tiagoolivoto.github.io/paper_mgidi_pm/. The source code used to produce the static website and the results in this manuscript have been archived at 10.5281/zenodo.7155173 as manuscript v2. [file 13007_2022_952_MOESM1_ESM.zip › TiagoOlivoto-paper_mgidi_pm-11ef6c1/docs/index.html]

About


MGIDI Plant Methods

- About
- Sup. Codes
- Sup. Figures
- Sup. Tables
- Code and data
- Doi

- metan

# About

## About

- About
- How to reproduce?

# About

In this website are available the data and scripts used in the manuscript *MGIDI: a powerful tool to analyze plant multivariate data*

# How to reproduce?

- Download data
- Open the `*.Rmd` file in RStudio.
- Optionally change functions and arguments.
- Knit the document to see the results.
