# Supplementary material for: MGIDI: a powerful tool to analyze plant multivariate data
Source: Plant Methods. 2022 Nov 12;18:121. doi: 10.1186/s13007-022-00952-5 (PMC9652799; doi:10.1186/s13007-022-00952-5)
Supplement: Supplementary file 1 — Additional file 1. A website with the data, script, and results is available at https://tiagoolivoto.github.io/paper_mgidi_pm/. The source code used to produce the static website and the results in this manuscript have been archived at 10.5281/zenodo.7155173 as manuscript v2. [file 13007_2022_952_MOESM1_ESM.zip › TiagoOlivoto-paper_mgidi_pm-11ef6c1/docs/sup_figures.html]

Supplementary figures


MGIDI Plant Methods

- About
- Sup. Codes
- Sup. Figures
- Sup. Tables
- Code and data
- Doi

- metan

# Supplementary figures

## Supplementary figures

- 1 Traits with negative desired gains
  - 1.1 AWNCF
  - 1.2 NDBF
  - 1.3 NDFF
  - 1.4 NDBH
  - 1.5 NNCF
  - 1.6 PHYL
  - 1.7 TA
  - 1.8 WNCF
  - 1.9 WUE
- 2 Traits with positive desired gains
  - 2.1 AWCF
  - 2.2 CHROMA
  - 2.3 FIRM
  - 2.4 FY
  - 2.5 H
  - 2.6 L
  - 2.7 NCF
  - 2.8 OAWF
  - 2.9 TNF
  - 2.10 TSS
  - 2.11 TSS\_TA
  - 2.12 TWF
  - 2.13 WCF
- 3 Water retention curve
- 4 Example (Aaby 2012)

# 1 Traits with negative desired gains

## 1.1 AWNCF

Figure 1.1: Average weight of non-commercial fruits. a: Cultivar (ALB: Albion; CAM: Camarosa) x origin (NAC: National; IMP: Imported) interaction; b: substrate main effect (S1: Sugarcane bagasse + organic compost; S2: Sugarcane bagasse + commercial substrate; S3: Rice husk + organic compost; and S4: Rice husk + commercial substrate). The horizonal dashed line shows the overall mean. Bars shows the mean +- standard error. N = 16.

## 1.2 NDBF

Figure 1.2: Begin of floration (days). a: Cultivar (ALB: Albion; CAM: Camarosa) x origin (NAC: National; IMP: Imported) interaction; b: substrate main effect (S1: Sugarcane bagasse + organic compost; S2: Sugarcane bagasse + commercial substrate; S3: Rice husk + organic compost; and S4: Rice husk + commercial substrate). The horizonal dashed line shows the overall mean. Bars shows the mean +- standard error. N = 16.

## 1.3 NDFF

Figure 1.3: Plene floration (days). a: Cultivar (ALB: Albion; CAM: Camarosa) x origin (NAC: National; IMP: Imported) interaction; b: substrate main effect (S1: Sugarcane bagasse + organic compost; S2: Sugarcane bagasse + commercial substrate; S3: Rice husk + organic compost; and S4: Rice husk + commercial substrate). The horizonal dashed line shows the overall mean. Bars shows the mean +- standard error. N = 16.

## 1.4 NDBH

Figure 1.4: Begin of harvest (days). a: Cultivar (ALB: Albion; CAM: Camarosa) x origin (NAC: National; IMP: Imported) interaction; b: substrate main effect (S1: Sugarcane bagasse + organic compost; S2: Sugarcane bagasse + commercial substrate; S3: Rice husk + organic compost; and S4: Rice husk + commercial substrate). The horizonal dashed line shows the overall mean. Bars shows the mean +- standard error. N = 16.

## 1.5 NNCF

Figure 1.5: Number of non-commercial fruits. a: Cultivar (ALB: Albion; CAM: Camarosa) x origin (NAC: National; IMP: Imported) interaction; b: substrate main effect (S1: Sugarcane bagasse + organic compost; S2: Sugarcane bagasse + commercial substrate; S3: Rice husk + organic compost; and S4: Rice husk + commercial substrate). The horizonal dashed line shows the overall mean. Bars shows the mean +- standard error. N = 16.

## 1.6 PHYL

Figure 1.6: Phyllochron. a: Cultivar (ALB: Albion; CAM: Camarosa) x origin (NAC: National; IMP: Imported) interaction; b: substrate main effect (S1: Sugarcane bagasse + organic compost; S2: Sugarcane bagasse + commercial substrate; S3: Rice husk + organic compost; and S4: Rice husk + commercial substrate). The horizonal dashed line shows the overall mean. Bars shows the mean +- standard error. N = 16.

## 1.7 TA

Figure 1.7: Total acid. a: Cultivar (ALB: Albion; CAM: Camarosa) x origin (NAC: National; IMP: Imported) interaction; b: substrate main effect (S1: Sugarcane bagasse + organic compost; S2: Sugarcane bagasse + commercial substrate; S3: Rice husk + organic compost; and S4: Rice husk + commercial substrate). The horizonal dashed line shows the overall mean. Bars shows the mean +- standard error. N = 16.

## 1.8 WNCF

Figure 1.8: Weight of non-commercial fruits. a: Cultivar (ALB: Albion; CAM: Camarosa) x origin (NAC: National; IMP: Imported) interaction; b: substrate main effect (S1: Sugarcane bagasse + organic compost; S2: Sugarcane bagasse + commercial substrate; S3: Rice husk + organic compost; and S4: Rice husk + commercial substrate). The horizonal dashed line shows the overall mean. Bars shows the mean +- standard error. N = 16.

## 1.9 WUE

Figure 1.9: Water use efficiency. a: Cultivar (ALB: Albion; CAM: Camarosa) x origin (NAC: National; IMP: Imported) interaction; b: substrate main effect (S1: Sugarcane bagasse + organic compost; S2: Sugarcane bagasse + commercial substrate; S3: Rice husk + organic compost; and S4: Rice husk + commercial substrate). The horizonal dashed line shows the overall mean. Bars shows the mean +- standard error. N = 16.

# 2 Traits with positive desired gains

## 2.1 AWCF

Figure 2.1: Average weigth of comercial fruits. a: Cultivar (ALB: Albion; CAM: Camarosa) x origin (NAC: National; IMP: Imported) interaction; b: substrate main effect (S1: Sugarcane bagasse + organic compost; S2: Sugarcane bagasse + commercial substrate; S3: Rice husk + organic compost; and S4: Rice husk + commercial substrate). The horizonal dashed line shows the overall mean. Bars shows the mean +- standard error. N = 16.

## 2.2 CHROMA

Figure 2.2: CHROMA. a: Cultivar (ALB: Albion; CAM: Camarosa) x origin (NAC: National; IMP: Imported) interaction; b: substrate main effect (S1: Sugarcane bagasse + organic compost; S2: Sugarcane bagasse + commercial substrate; S3: Rice husk + organic compost; and S4: Rice husk + commercial substrate). The horizonal dashed line shows the overall mean. Bars shows the mean +- standard error. N = 16.

## 2.3 FIRM

Figure 2.3: Flesh firmness. a: Cultivar (ALB: Albion; CAM: Camarosa) x origin (NAC: National; IMP: Imported) interaction; b: substrate main effect (S1: Sugarcane bagasse + organic compost; S2: Sugarcane bagasse + commercial substrate; S3: Rice husk + organic compost; and S4: Rice husk + commercial substrate). The horizonal dashed line shows the overall mean. Bars shows the mean +- standard error. N = 16.

## 2.4 FY

Figure 2.4: Fruit yield. a: Cultivar (ALB: Albion; CAM: Camarosa) x origin (NAC: National; IMP: Imported) interaction; b: substrate main effect (S1: Sugarcane bagasse + organic compost; S2: Sugarcane bagasse + commercial substrate; S3: Rice husk + organic compost; and S4: Rice husk + commercial substrate). The horizonal dashed line shows the overall mean. Bars shows the mean +- standard error. N = 16.

## 2.5 H

Figure 2.5: Hue angle. a: Cultivar (ALB: Albion; CAM: Camarosa) x origin (NAC: National; IMP: Imported) interaction; b: substrate main effect (S1: Sugarcane bagasse + organic compost; S2: Sugarcane bagasse + commercial substrate; S3: Rice husk + organic compost; and S4: Rice husk + commercial substrate). The horizonal dashed line shows the overall mean. Bars shows the mean +- standard error. N = 16.

## 2.6 L

Figure 2.6: L. a: Cultivar (ALB: Albion; CAM: Camarosa) x origin (NAC: National; IMP: Imported) interaction; b: substrate main effect (S1: Sugarcane bagasse + organic compost; S2: Sugarcane bagasse + commercial substrate; S3: Rice husk + organic compost; and S4: Rice husk + commercial substrate). The horizonal dashed line shows the overall mean. Bars shows the mean +- standard error. N = 16.

## 2.7 NCF

Figure 2.7: Number of commercial fruits. a: Cultivar (ALB: Albion; CAM: Camarosa) x origin (NAC: National; IMP: Imported) interaction; b: substrate main effect (S1: Sugarcane bagasse + organic compost; S2: Sugarcane bagasse + commercial substrate; S3: Rice husk + organic compost; and S4: Rice husk + commercial substrate). The horizonal dashed line shows the overall mean. Bars shows the mean +- standard error. N = 16.

## 2.8 OAWF

Figure 2.8: Overall average of weigth of fruits. a: Cultivar (ALB: Albion; CAM: Camarosa) x origin (NAC: National; IMP: Imported) interaction; b: substrate main effect (S1: Sugarcane bagasse + organic compost; S2: Sugarcane bagasse + commercial substrate; S3: Rice husk + organic compost; and S4: Rice husk + commercial substrate). The horizonal dashed line shows the overall mean. Bars shows the mean +- standard error. N = 16.

## 2.9 TNF

Figure 2.9: Total number of fruits. a: Cultivar (ALB: Albion; CAM: Camarosa) x origin (NAC: National; IMP: Imported) interaction; b: substrate main effect (S1: Sugarcane bagasse + organic compost; S2: Sugarcane bagasse + commercial substrate; S3: Rice husk + organic compost; and S4: Rice husk + commercial substrate). The horizonal dashed line shows the overall mean. Bars shows the mean +- standard error. N = 16.

## 2.10 TSS

Figure 2.10: Total soluble solids. a: Cultivar (ALB: Albion; CAM: Camarosa) x origin (NAC: National; IMP: Imported) interaction; b: substrate main effect (S1: Sugarcane bagasse + organic compost; S2: Sugarcane bagasse + commercial substrate; S3: Rice husk + organic compost; and S4: Rice husk + commercial substrate). The horizonal dashed line shows the overall mean. Bars shows the mean +- standard error. N = 16.

## 2.11 TSS\_TA

Figure 2.11: TSS/TA ratio. a: Cultivar (ALB: Albion; CAM: Camarosa) x origin (NAC: National; IMP: Imported) interaction; b: substrate main effect (S1: Sugarcane bagasse + organic compost; S2: Sugarcane bagasse + commercial substrate; S3: Rice husk + organic compost; and S4: Rice husk + commercial substrate). The horizonal dashed line shows the overall mean. Bars shows the mean +- standard error. N = 16.

## 2.12 TWF

Figure 2.12: Total weight of fruits. a: Cultivar (ALB: Albion; CAM: Camarosa) x origin (NAC: National; IMP: Imported) interaction; b: substrate main effect (S1: Sugarcane bagasse + organic compost; S2: Sugarcane bagasse + commercial substrate; S3: Rice husk + organic compost; and S4: Rice husk + commercial substrate). The horizonal dashed line shows the overall mean. Bars shows the mean +- standard error. N = 16.

## 2.13 WCF

Figure 2.13: Weight of commercial fruits. a: Cultivar (ALB: Albion; CAM: Camarosa) x origin (NAC: National; IMP: Imported) interaction; b: substrate main effect (S1: Sugarcane bagasse + organic compost; S2: Sugarcane bagasse + commercial substrate; S3: Rice husk + organic compost; and S4: Rice husk + commercial substrate). The horizonal dashed line shows the overall mean. Bars shows the mean +- standard error. N = 16.

# 3 Water retention curve

Figure 3.1: Water retention curve for different substrates used in the experiment.

# 4 Example (Aaby 2012)

In this example, we compute the MGIDI index for ranking 32 cultivars based on 18 traits. The original article is found at http://link.springer.com/10.1007/BF02289233

```
mgidi_aaby <- 
  import("data/df_aaby.xlsx") |> 
  tidy_colnames() |> 
  column_to_rownames("CULTIVAR") |> 
  mgidi(SI = 2)
## 
## -------------------------------------------------------------------------------
## Principal Component Analysis
## -------------------------------------------------------------------------------
## # A tibble: 18 × 4
##    PC    Eigenvalues `Variance (%)` `Cum. variance (%)`
##    <chr>       <dbl>          <dbl>               <dbl>
##  1 PC1          6.12          34.0                 34.0
##  2 PC2          3.4           18.9                 52.9
##  3 PC3          1.92          10.7                 63.6
##  4 PC4          1.46           8.11                71.7
##  5 PC5          1.06           5.86                77.5
##  6 PC6          0.87           4.85                82.4
##  7 PC7          0.7            3.9                 86.3
##  8 PC8          0.6            3.36                89.6
##  9 PC9          0.44           2.46                92.1
## 10 PC10         0.36           1.99                94.1
## 11 PC11         0.29           1.64                95.7
## 12 PC12         0.26           1.44                97.2
## 13 PC13         0.19           1.08                98.2
## 14 PC14         0.12           0.68                98.9
## 15 PC15         0.1            0.58                99.5
## 16 PC16         0.06           0.35                99.8
## 17 PC17         0.03           0.17               100  
## 18 PC18         0              0                  100  
## -------------------------------------------------------------------------------
## Factor Analysis - factorial loadings after rotation-
## -------------------------------------------------------------------------------
## # A tibble: 18 × 8
##    VAR                          FA1   FA2   FA3   FA4   FA5 Communality Unique…¹
##    <chr>                      <dbl> <dbl> <dbl> <dbl> <dbl>       <dbl>    <dbl>
##  1 CYD_3_GLU                  -0.04  0.02  0.21  0.05 -0.92        0.89     0.11
##  2 PG_3_GLU                   -0.17 -0.21  0.61  0.3  -0.59        0.88     0.12
##  3 PG_3_RUT                   -0.8  -0.19  0.13  0.19 -0.22        0.77     0.23
##  4 CYD_3_MALGLU               -0.57  0.05  0.08 -0.11 -0.76        0.93     0.07
##  5 PG_3_MALGLU                -0.81 -0.09  0.31 -0.14 -0.3         0.86     0.14
##  6 PG_3_ACETGLU                0.68  0.18  0.48 -0.07 -0.26        0.8      0.2 
##  7 TOTAL_ANTHOCY_             -0.47 -0.19  0.55  0.17 -0.6         0.95     0.05
##  8 QUERCETIN_GLYCOSIDESC_D     0.05 -0.28  0.21  0.69 -0.47        0.82     0.18
##  9 KAEMPFEROL_GLYCOSIDESD_E   -0.54 -0.23  0.14  0.49 -0.12        0.62     0.38
## 10 _CATECHINF_G               -0.08 -0.69 -0.55  0.09 -0.08        0.8      0.2 
## 11 PROCYANIDIN_DIMERSG_H      -0.44 -0.59  0.05  0.48 -0.01        0.77     0.23
## 12 PROCYANIDIN_TRIMERG        -0.25 -0.79 -0.08  0.18 -0.18        0.76     0.24
## 13 AGRIMONIINC                -0.06 -0.79  0.06 -0.21 -0.04        0.67     0.33
## 14 OTHER_ETC_CD               -0.17 -0.79  0.1   0.16  0.13        0.7      0.3 
## 15 ELLAGIC_ACIDE              -0.08 -0.53 -0.29  0.38 -0.39        0.67     0.33
## 16 ELLAGIC_ACID_GLYCOSIDESE_F  0.21 -0.55  0.12  0.53  0.04        0.64     0.36
## 17 COUMAROYL_HEXOSESG         -0.15  0.09 -0.14  0.86  0.06        0.79     0.21
## 18 CINNAMOYL_GLUCOSEH          0.17 -0.04 -0.76  0.04  0.2         0.64     0.36
## # … with abbreviated variable name ¹​Uniquenesses
## -------------------------------------------------------------------------------
## Comunalit Mean: 0.7752721 
## -------------------------------------------------------------------------------
## Selection differential 
## -------------------------------------------------------------------------------
## # A tibble: 18 × 8
##    VAR                        Factor      Xo    Xs      SD  SDperc sense    goal
##    <chr>                      <chr>    <dbl> <dbl>   <dbl>   <dbl> <chr>   <dbl>
##  1 PG_3_RUT                   FA1     1.13     2.2  1.07     94.5  increa…   100
##  2 PG_3_MALGLU                FA1     5.99    12.3  6.31    105.   increa…   100
##  3 PG_3_ACETGLU               FA1     0.0812   0   -0.0812 -100    increa…     0
##  4 KAEMPFEROL_GLYCOSIDESD_E   FA1     0.844    1.5  0.656    77.8  increa…   100
##  5 _CATECHINF_G               FA2     4.47     6.1  1.63     36.4  increa…   100
##  6 PROCYANIDIN_DIMERSG_H      FA2     9.06    13.3  4.24     46.8  increa…   100
##  7 PROCYANIDIN_TRIMERG        FA2     7.90    12.4  4.50     57.0  increa…   100
##  8 AGRIMONIINC                FA2     8.83    12.6  3.77     42.7  increa…   100
##  9 OTHER_ETC_CD               FA2     2.78     2.7 -0.0781   -2.81 increa…     0
## 10 ELLAGIC_ACIDE              FA2     0.519    0.7  0.181    34.9  increa…   100
## 11 ELLAGIC_ACID_GLYCOSIDESE_F FA2     0.581    0.8  0.219    37.6  increa…   100
## 12 PG_3_GLU                   FA3    25.3     47.7 22.4      88.8  increa…   100
## 13 CINNAMOYL_GLUCOSEH         FA3     4.98     3.5 -1.48    -29.8  increa…     0
## 14 QUERCETIN_GLYCOSIDESC_D    FA4     1.8      5.2  3.4     189.   increa…   100
## 15 COUMAROYL_HEXOSESG         FA4     5.44     7    1.56     28.6  increa…   100
## 16 CYD_3_GLU                  FA5     1.09     2.9  1.81    167.   increa…   100
## 17 CYD_3_MALGLU               FA5     0.356    0.9  0.544   153.   increa…   100
## 18 TOTAL_ANTHOCY_             FA5    33.9     65.9 32.0      94.3  increa…   100
## ------------------------------------------------------------------------------
## Selected genotypes
## -------------------------------------------------------------------------------
## Rondo
## -------------------------------------------------------------------------------

plot(mgidi_aaby)
```
