# Supplementary material for: MGIDI: a powerful tool to analyze plant multivariate data
Source: Plant Methods. 2022 Nov 12;18:121. doi: 10.1186/s13007-022-00952-5 (PMC9652799; doi:10.1186/s13007-022-00952-5)
Supplement: Supplementary file 1 — Additional file 1. A website with the data, script, and results is available at https://tiagoolivoto.github.io/paper_mgidi_pm/. The source code used to produce the static website and the results in this manuscript have been archived at 10.5281/zenodo.7155173 as manuscript v2. [file 13007_2022_952_MOESM1_ESM.zip › TiagoOlivoto-paper_mgidi_pm-11ef6c1/docs/sup_tables.html]

Supplementary tables


MGIDI Plant Methods

- About
- Sup. Codes
- Sup. Figures
- Sup. Tables
- Code and data
- Doi

- metan

# Supplementary tables

## Supplementary tables

- 1 Hidroponic solution
- 2 Substrate
- 3 Univariate selection

# 1 Hidroponic solution

Table 1.1: Doses of mineral fertilizers in 1000 liters of water for strawberry fertigation in a substrate of pH 7 or above.

| Fertilizers | Vegetative Phase | Reproductive Phase |
| --- | --- | --- |
| Calcium nitrate (15.5-00-00) | 480 g | 480 g |
| Potassium nitrate (12-00-45) | 300 g | 180 g |
| Magnesium Sulphate (00-00-00-09) | 360 g | 360 g |
| Ammonium sulfate (20-00-00) | 50 g | 70 g |
| Potassium Sulphate (00-00-50) | 70 g | 260 g |
| Phosphoric acid (85%) | 110 ml | 110 ml |
| Boric acid(17%B) | 1.8 g | 1.8 g |
| Copper sulphate (25%Cu) | 0.18 g | 0.18 g |
| Manganese Sulfate (25%Mn) | 1.2 g | 1.2 g |
| Zinc sulfate (20% Zn) | 0.6 g | 0.6 g |
| Sodium Molybdate (39%Mo) | 0.18 g | 0.18 g |
| Chelated iron (6% Fe) | 36 g | 36 g |

# 2 Substrate

Table 2.1: Concentrations of ingredients used to formulate each one of the four substrates used in the experiment.

| Substracts | Sugarcane bagasse (%) | Rice husk (%) | Organic (%) | Carolina (%) |
| --- | --- | --- | --- | --- |
| S1 | 70 | 0 | 30 | 0 |
| S2 | 70 | 0 | 0 | 30 |
| S3 | 0 | 70 | 30 | 0 |
| S4 | 0 | 70 | 0 | 30 |

Table 2.2: Density (DS), total porosity (TP), aeration space (AS), readily available water (RAW), buffer water (BW), available water (AW) and remaining water (AR) for the substrates used: S1: Sugarcane bagasse + organic compost; S2: Sugarcane bagasse + commercial substrate - Carolina; S3: Rice husk + organic compost; and S4: Rice husk + commercial substrate - Carolina.

| Substracts | DS | TP | AS | RAW | BW | AW | RW |
| --- | --- | --- | --- | --- | --- | --- | --- |
| S1 | 0.14 | 0.74 | 0.48 | 0.05 | 0.01 | 0.06 | 0.20 |
| S2 | 0.09 | 0.75 | 0.46 | 0.09 | 0.02 | 0.11 | 0.18 |
| S3 | 0.23 | 0.63 | 0.27 | 0.15 | 0.03 | 0.18 | 0.18 |
| S4 | 0.13 | 0.67 | 0.29 | 0.24 | 0.08 | 0.32 | 0.05 |

# 3 Univariate selection

Table 3.1: Selection differential for mean performance based on the direct and univariate selection on fruit yield (Camarosa cultivar

| TRAIT | Xo | Xs | SD | SD (%) | Goal | Success |
| --- | --- | --- | --- | --- | --- | --- |
| NNCF | 7.20 | 9.36 | 2.15 | 29.87 | Low | No |
| WNCF | 65.36 | 77.61 | 12.26 | 18.75 | Low | No |
| AWNCF | 8.74 | 8.17 | -0.57 | -6.48 | Low | Yes |
| WUE | 115.84 | 88.10 | -27.73 | -23.94 | Low | Yes |
| NDBF | 57.70 | 66.19 | 8.48 | 14.70 | Low | No |
| NDFF | 76.27 | 83.56 | 7.30 | 9.57 | Low | No |
| NDBH | 77.80 | 86.94 | 9.14 | 11.75 | Low | No |
| PHYL | 164.45 | 153.01 | -11.44 | -6.96 | Low | Yes |
| TA | 1.42 | 1.15 | -0.27 | -19.26 | Low | Yes |
| NCF | 23.47 | 28.76 | 5.29 | 22.54 | High | Yes |
| TNF | 30.66 | 38.12 | 7.45 | 24.30 | High | Yes |
| WCF | 358.57 | 458.50 | 99.93 | 27.87 | High | Yes |
| TWF | 412.73 | 491.95 | 79.22 | 19.19 | High | Yes |
| AWCF | 14.85 | 14.48 | -0.36 | -2.45 | High | No |
| OAWF | 13.54 | 12.86 | -0.68 | -5.00 | High | No |
| FY | 30957.06 | 36896.05 | 5938.99 | 19.18 | High | Yes |
| TSS | 7.43 | 6.73 | -0.70 | -9.38 | High | No |
| TSS\_TA | 5.39 | 6.03 | 0.64 | 11.82 | High | Yes |
| FIRM | 1.67 | 1.55 | -0.12 | -6.97 | High | No |
| L | 54.81 | 56.22 | 1.41 | 2.57 | High | Yes |
| CHROMA | 44.60 | 43.04 | -1.56 | -3.50 | High | No |
| H | 43.07 | 42.52 | -0.55 | -1.28 | High | No |
