# Supplementary figures and images for: MGIDI: a powerful tool to analyze plant multivariate data
Source: Plant Methods. 2022 Nov 12;18:121. doi: 10.1186/s13007-022-00952-5 (PMC9652799; doi:10.1186/s13007-022-00952-5)

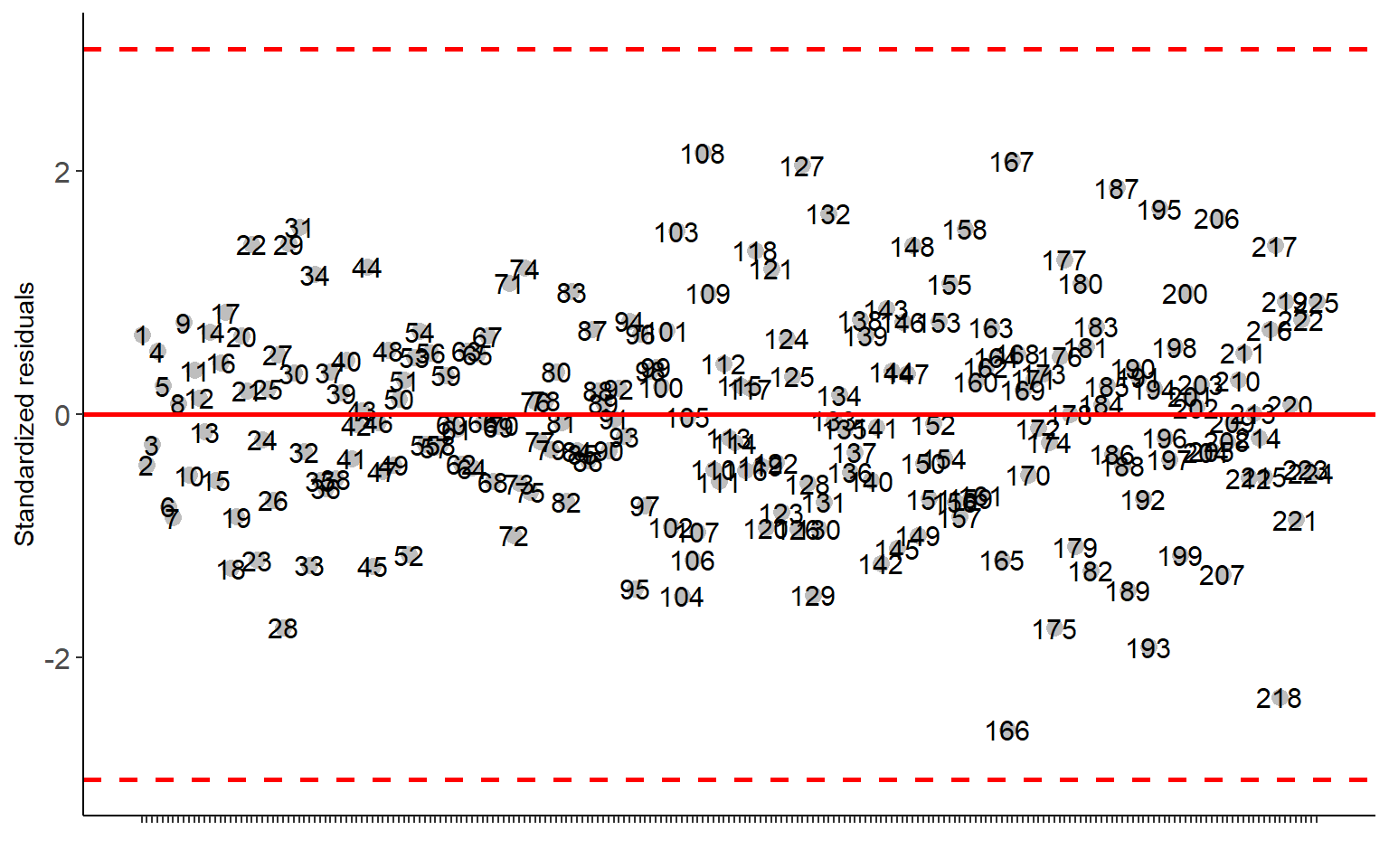

Supplement: Supplementary file 1 — Additional file 1. A website with the data, script, and results is available at https://tiagoolivoto.github.io/paper_mgidi_pm/. The source code used to produce the static website and the results in this manuscript have been archived at 10.5281/zenodo.7155173 as manuscript v2. [file 13007_2022_952_MOESM1_ESM.zip › TiagoOlivoto-paper_mgidi_pm-11ef6c1/docs/code_files/figure-html/unnamed-chunk-10-1.png]

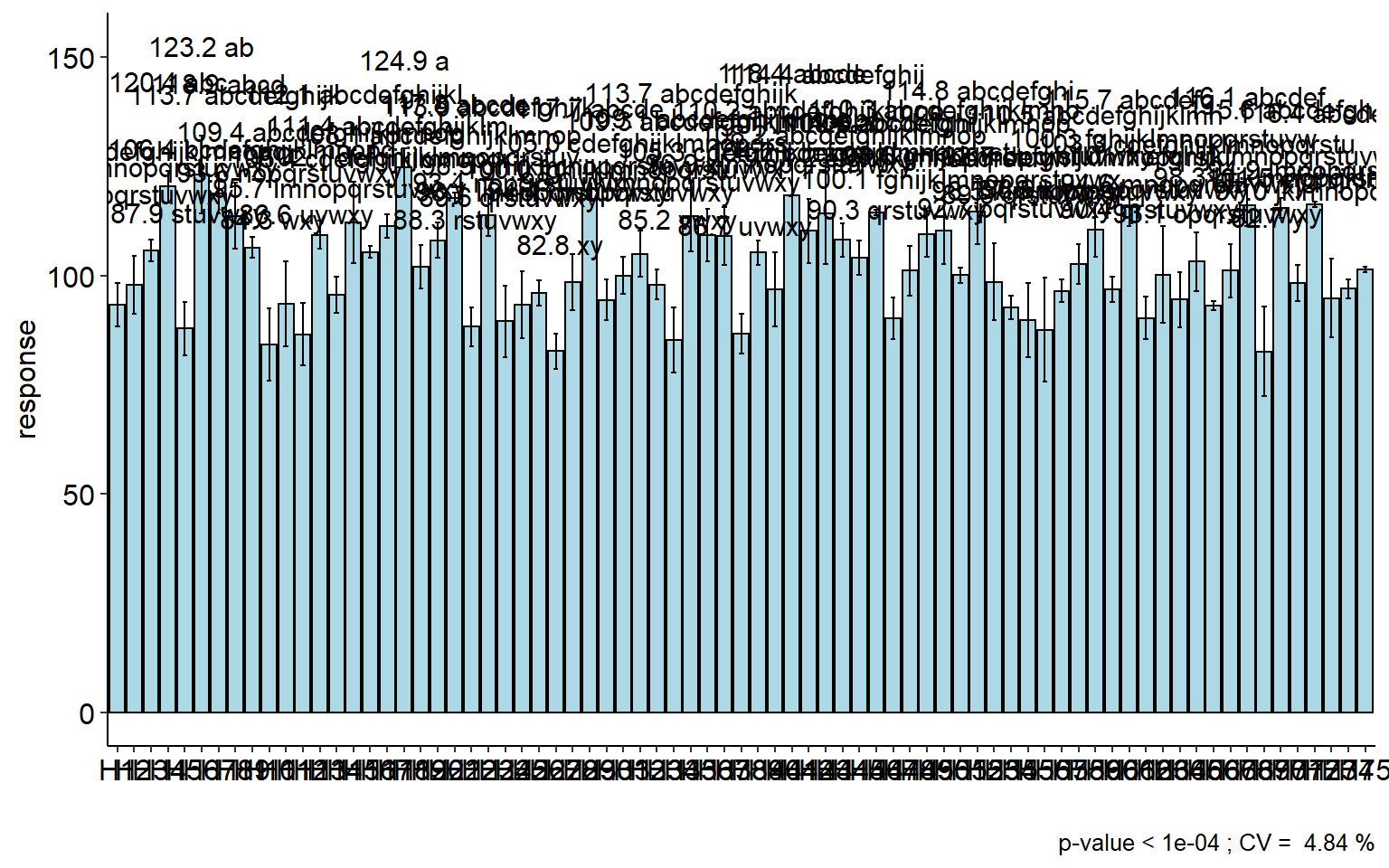

Supplement: Supplementary file 1 — Additional file 1. A website with the data, script, and results is available at https://tiagoolivoto.github.io/paper_mgidi_pm/. The source code used to produce the static website and the results in this manuscript have been archived at 10.5281/zenodo.7155173 as manuscript v2. [file 13007_2022_952_MOESM1_ESM.zip › TiagoOlivoto-paper_mgidi_pm-11ef6c1/docs/code_files/figure-html/unnamed-chunk-10-2.png]

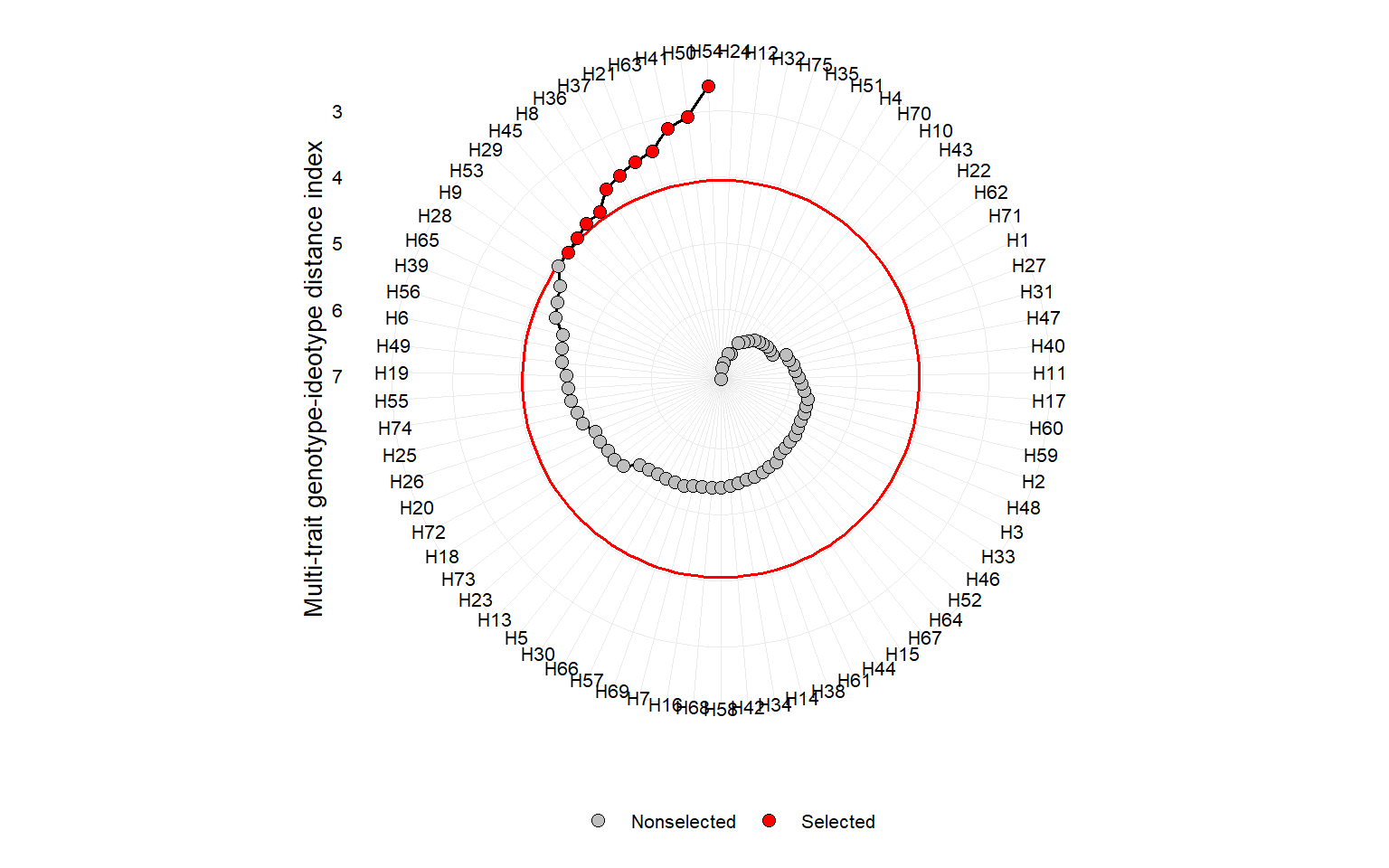

Supplement: Supplementary file 1 — Additional file 1. A website with the data, script, and results is available at https://tiagoolivoto.github.io/paper_mgidi_pm/. The source code used to produce the static website and the results in this manuscript have been archived at 10.5281/zenodo.7155173 as manuscript v2. [file 13007_2022_952_MOESM1_ESM.zip › TiagoOlivoto-paper_mgidi_pm-11ef6c1/docs/code_files/figure-html/unnamed-chunk-11-1.png]

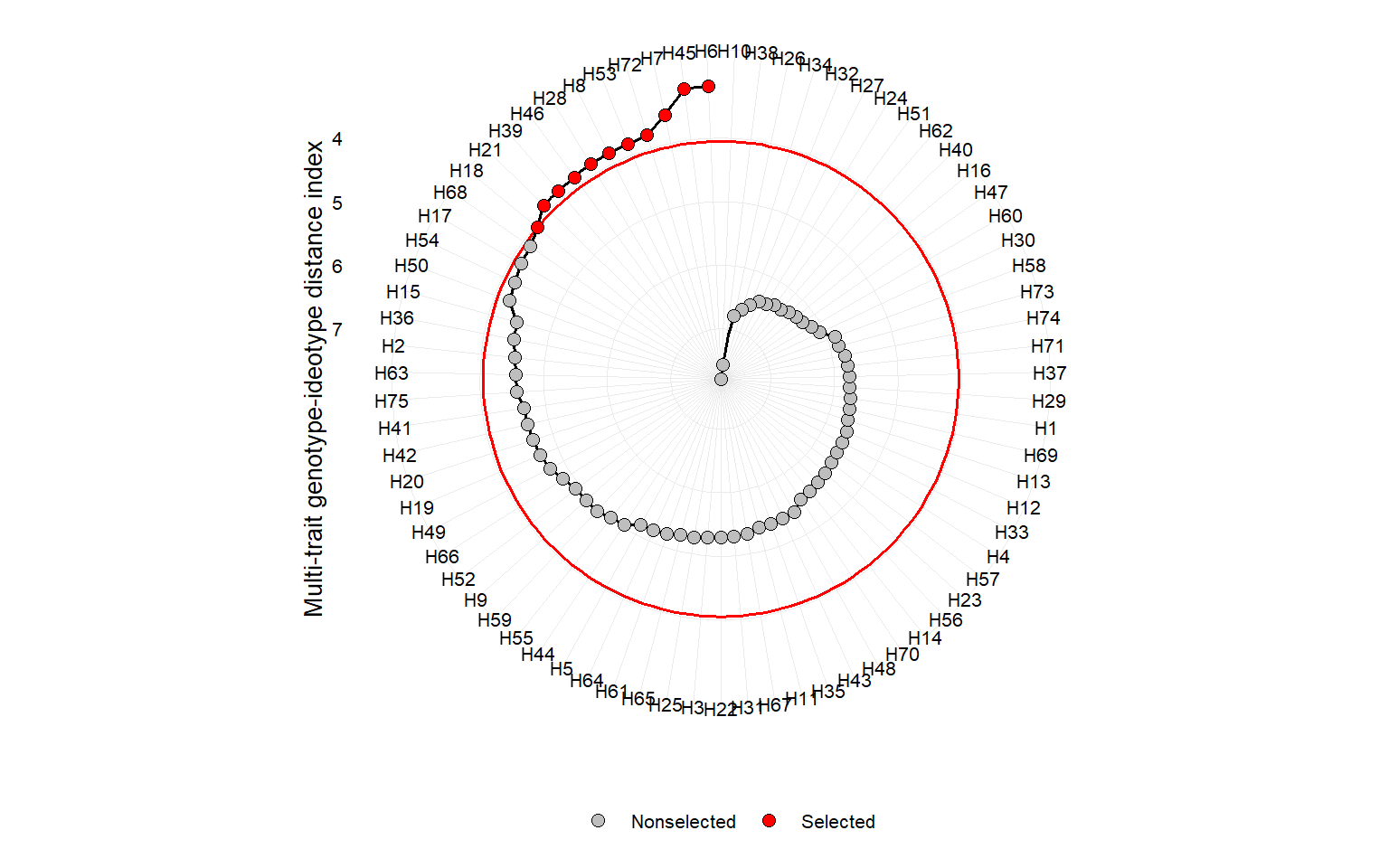

Supplement: Supplementary file 1 — Additional file 1. A website with the data, script, and results is available at https://tiagoolivoto.github.io/paper_mgidi_pm/. The source code used to produce the static website and the results in this manuscript have been archived at 10.5281/zenodo.7155173 as manuscript v2. [file 13007_2022_952_MOESM1_ESM.zip › TiagoOlivoto-paper_mgidi_pm-11ef6c1/docs/code_files/figure-html/unnamed-chunk-11-2.png]

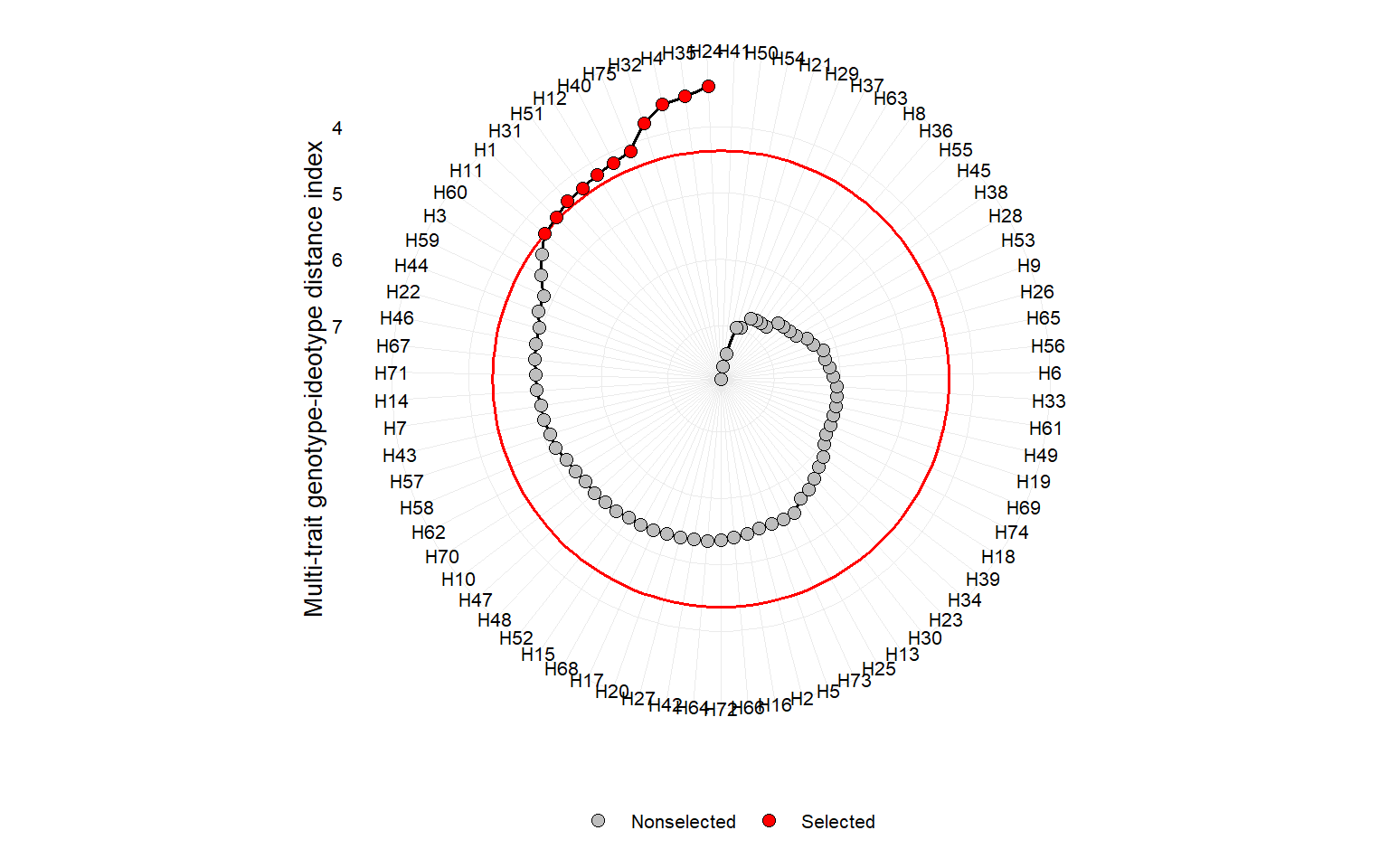

Supplement: Supplementary file 1 — Additional file 1. A website with the data, script, and results is available at https://tiagoolivoto.github.io/paper_mgidi_pm/. The source code used to produce the static website and the results in this manuscript have been archived at 10.5281/zenodo.7155173 as manuscript v2. [file 13007_2022_952_MOESM1_ESM.zip › TiagoOlivoto-paper_mgidi_pm-11ef6c1/docs/code_files/figure-html/unnamed-chunk-11-3.png]

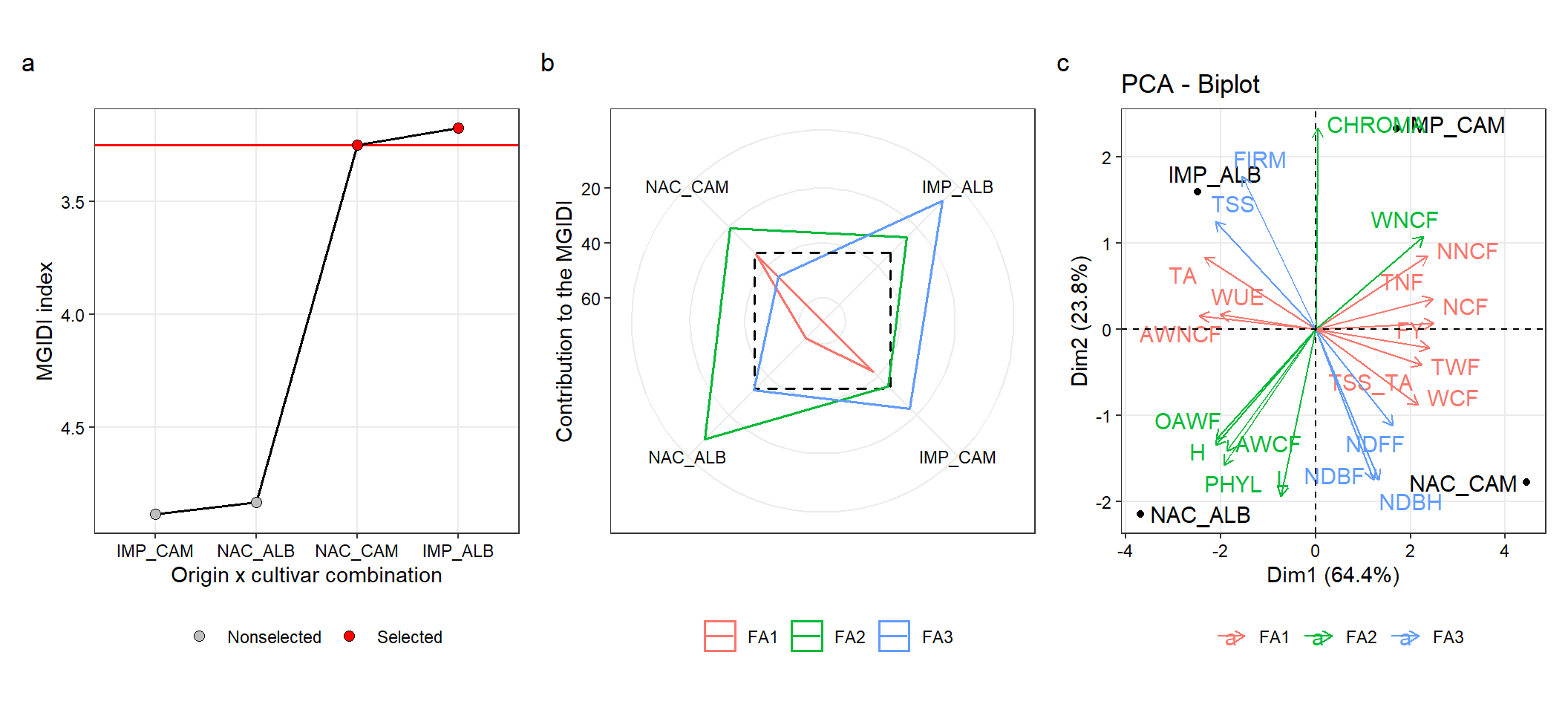

Supplement: Supplementary file 1 — Additional file 1. A website with the data, script, and results is available at https://tiagoolivoto.github.io/paper_mgidi_pm/. The source code used to produce the static website and the results in this manuscript have been archived at 10.5281/zenodo.7155173 as manuscript v2. [file 13007_2022_952_MOESM1_ESM.zip › TiagoOlivoto-paper_mgidi_pm-11ef6c1/docs/code_files/figure-html/unnamed-chunk-19-1.png]

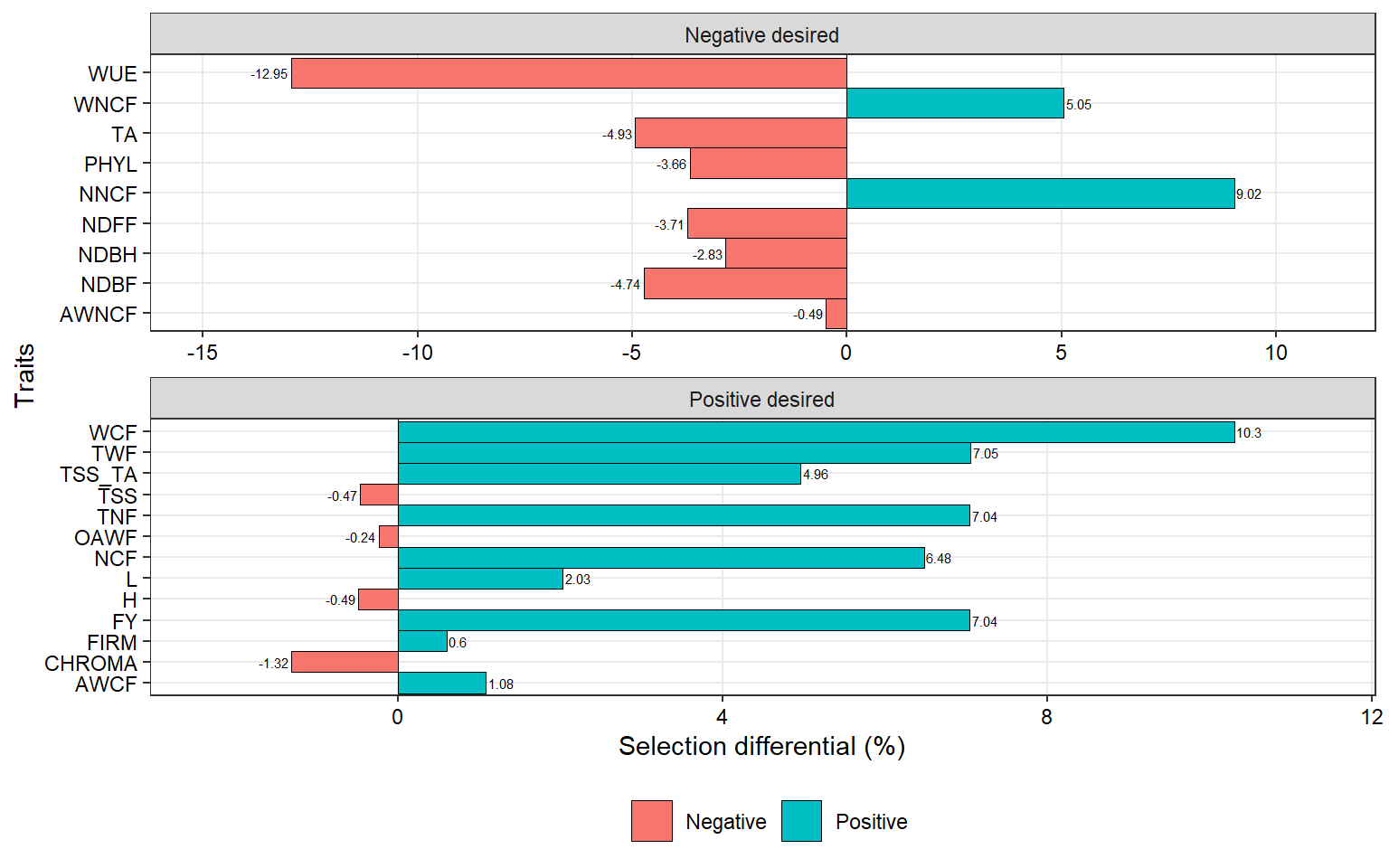

Supplement: Supplementary file 1 — Additional file 1. A website with the data, script, and results is available at https://tiagoolivoto.github.io/paper_mgidi_pm/. The source code used to produce the static website and the results in this manuscript have been archived at 10.5281/zenodo.7155173 as manuscript v2. [file 13007_2022_952_MOESM1_ESM.zip › TiagoOlivoto-paper_mgidi_pm-11ef6c1/docs/code_files/figure-html/unnamed-chunk-20-1.png]

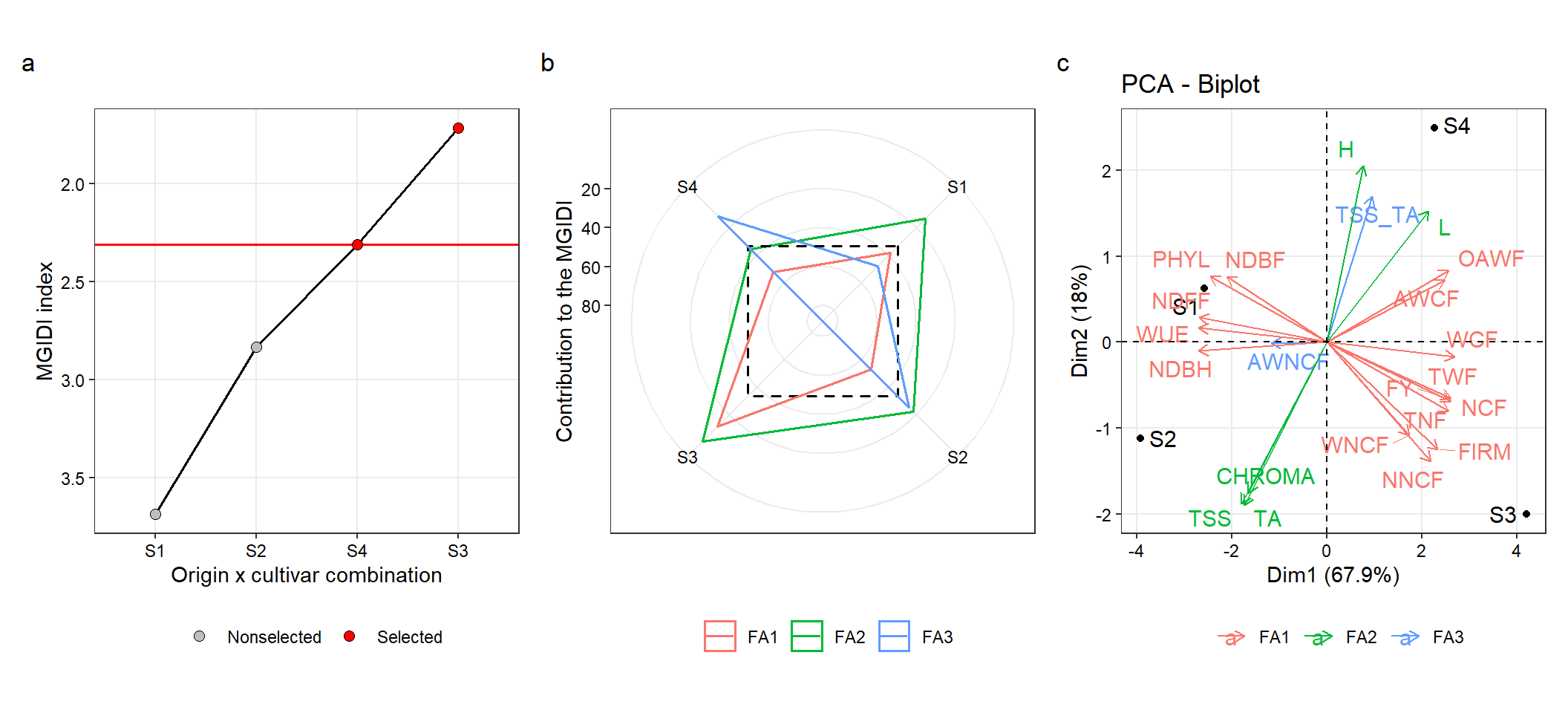

Supplement: Supplementary file 1 — Additional file 1. A website with the data, script, and results is available at https://tiagoolivoto.github.io/paper_mgidi_pm/. The source code used to produce the static website and the results in this manuscript have been archived at 10.5281/zenodo.7155173 as manuscript v2. [file 13007_2022_952_MOESM1_ESM.zip › TiagoOlivoto-paper_mgidi_pm-11ef6c1/docs/code_files/figure-html/unnamed-chunk-24-1.png]

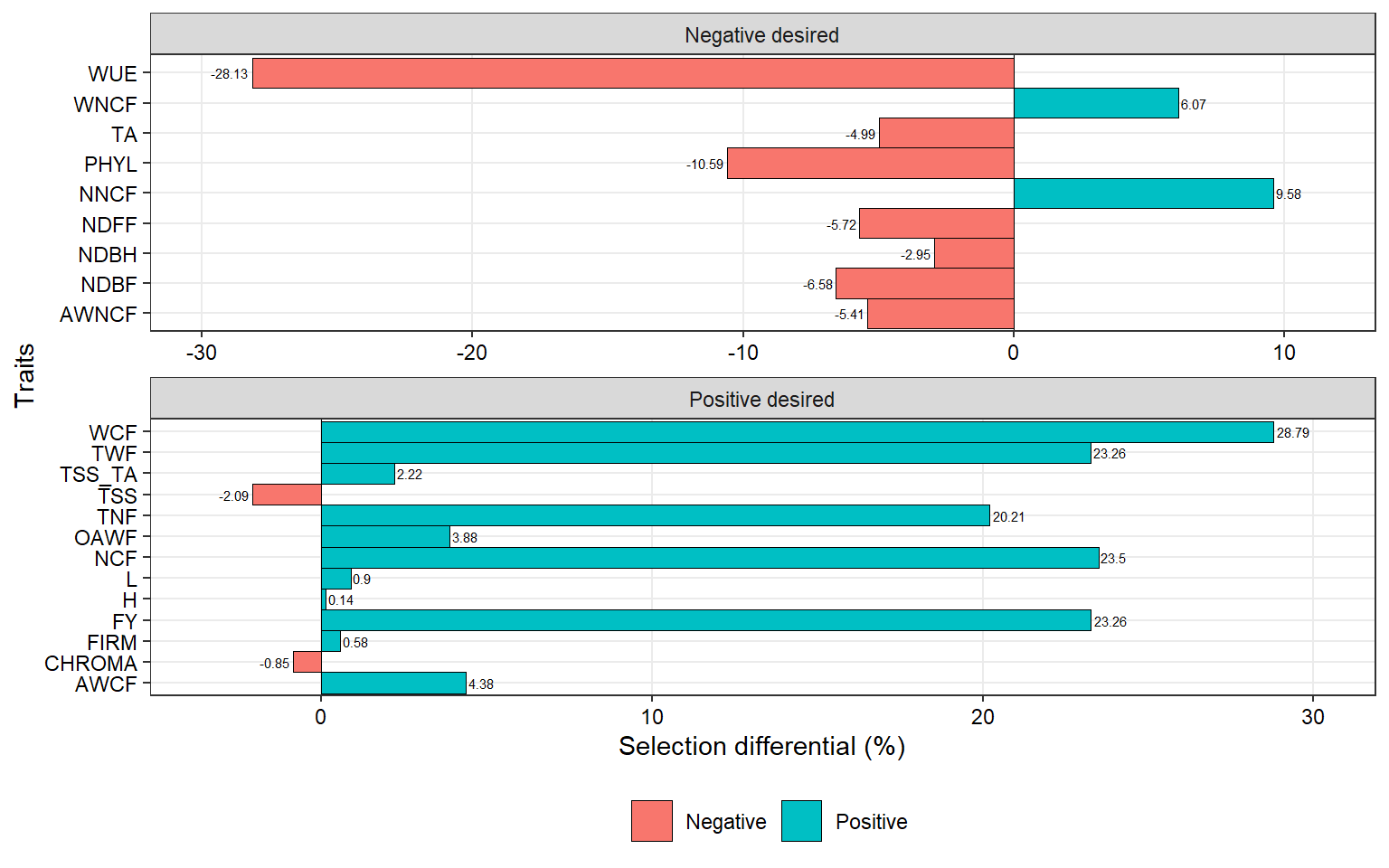

Supplement: Supplementary file 1 — Additional file 1. A website with the data, script, and results is available at https://tiagoolivoto.github.io/paper_mgidi_pm/. The source code used to produce the static website and the results in this manuscript have been archived at 10.5281/zenodo.7155173 as manuscript v2. [file 13007_2022_952_MOESM1_ESM.zip › TiagoOlivoto-paper_mgidi_pm-11ef6c1/docs/code_files/figure-html/unnamed-chunk-25-1.png]

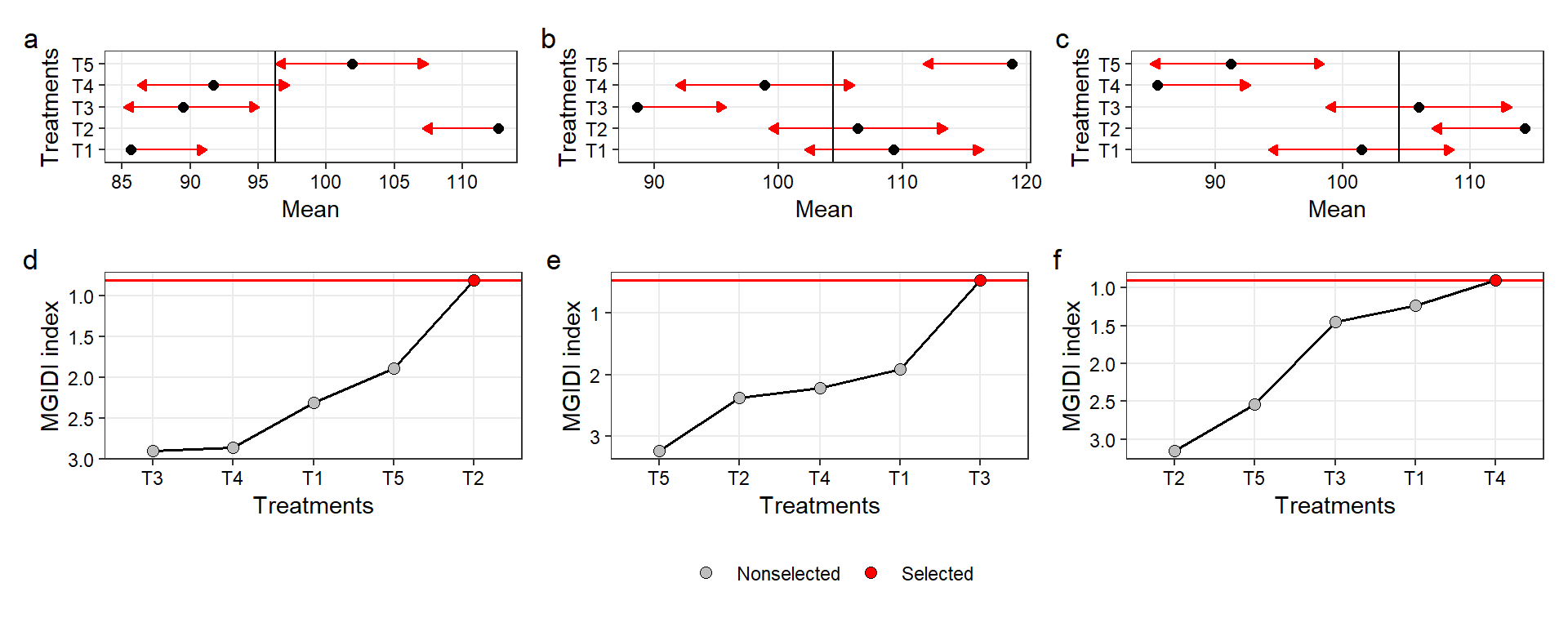

Supplement: Supplementary file 1 — Additional file 1. A website with the data, script, and results is available at https://tiagoolivoto.github.io/paper_mgidi_pm/. The source code used to produce the static website and the results in this manuscript have been archived at 10.5281/zenodo.7155173 as manuscript v2. [file 13007_2022_952_MOESM1_ESM.zip › TiagoOlivoto-paper_mgidi_pm-11ef6c1/docs/code_files/figure-html/unnamed-chunk-5-1.png]

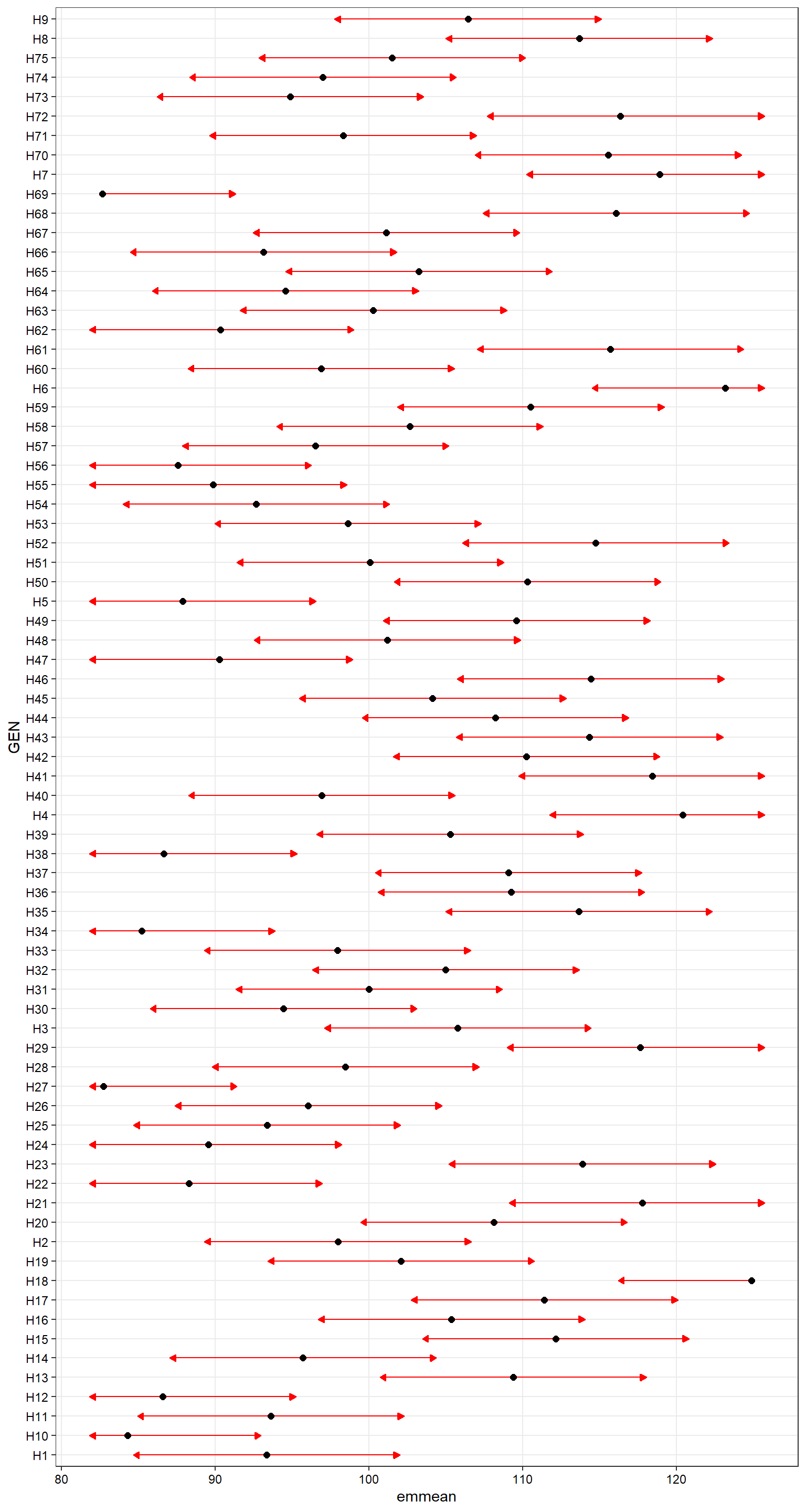

Supplement: Supplementary file 1 — Additional file 1. A website with the data, script, and results is available at https://tiagoolivoto.github.io/paper_mgidi_pm/. The source code used to produce the static website and the results in this manuscript have been archived at 10.5281/zenodo.7155173 as manuscript v2. [file 13007_2022_952_MOESM1_ESM.zip › TiagoOlivoto-paper_mgidi_pm-11ef6c1/docs/code_files/figure-html/unnamed-chunk-8-1.png]

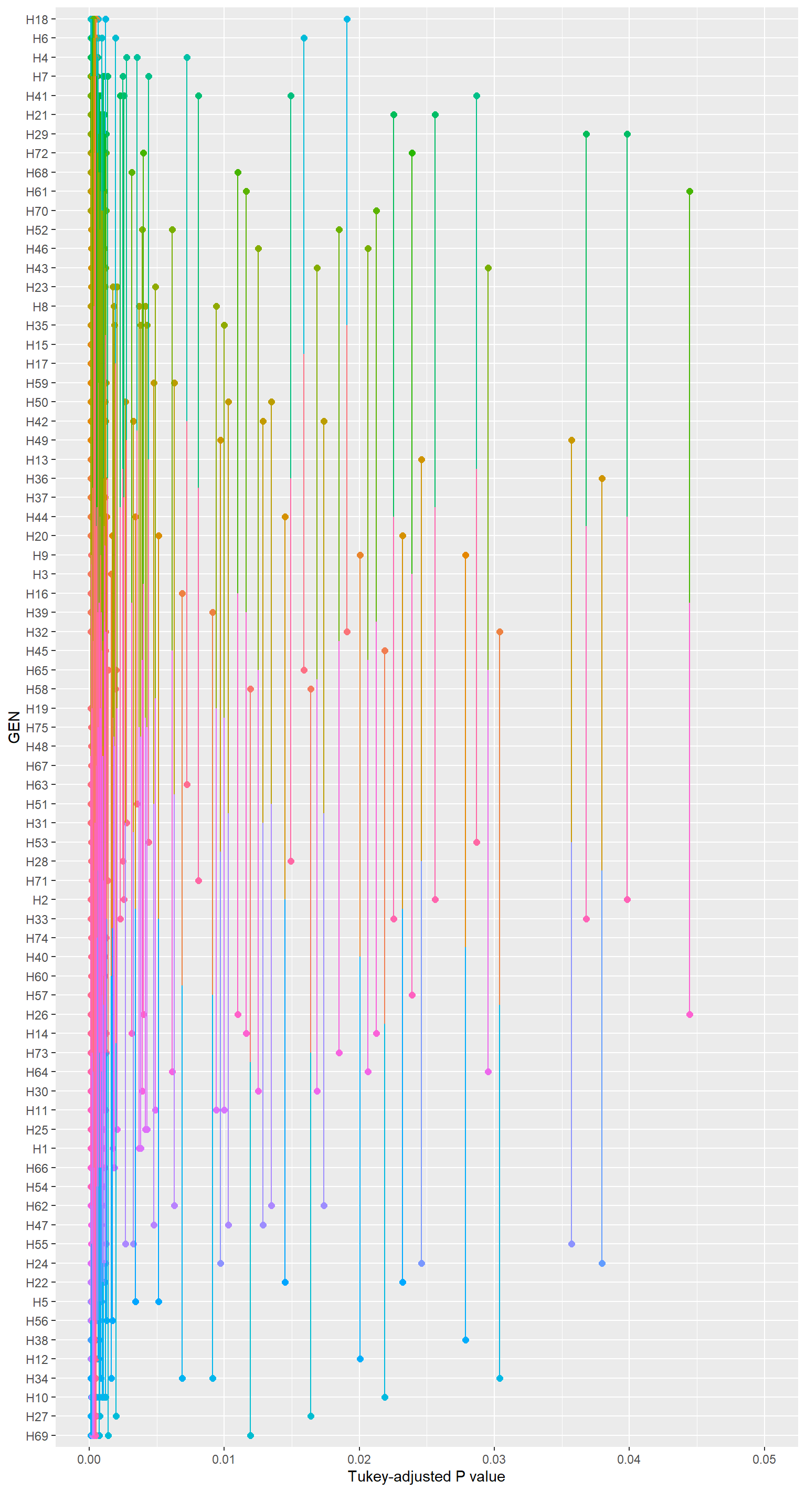

Supplement: Supplementary file 1 — Additional file 1. A website with the data, script, and results is available at https://tiagoolivoto.github.io/paper_mgidi_pm/. The source code used to produce the static website and the results in this manuscript have been archived at 10.5281/zenodo.7155173 as manuscript v2. [file 13007_2022_952_MOESM1_ESM.zip › TiagoOlivoto-paper_mgidi_pm-11ef6c1/docs/code_files/figure-html/unnamed-chunk-9-1.png]

a

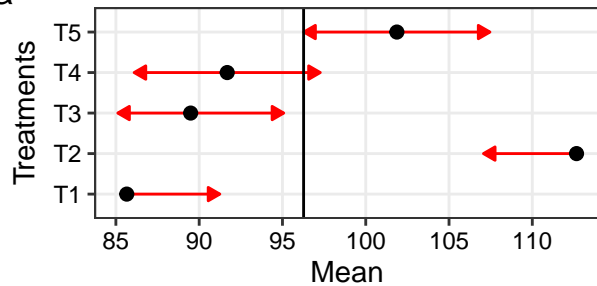

b

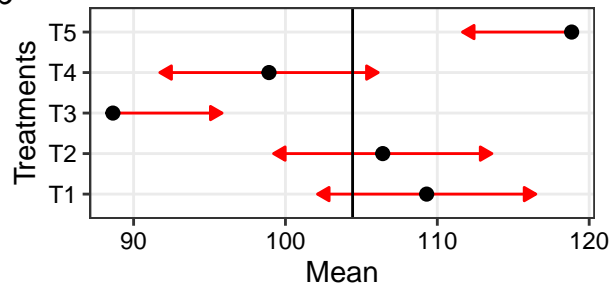

c

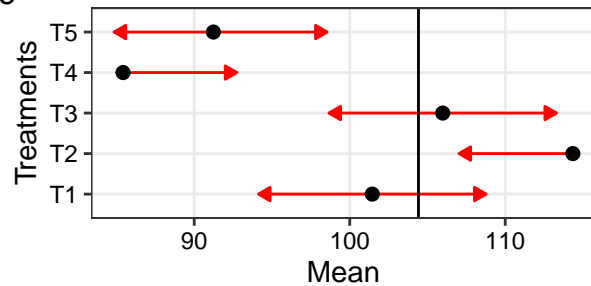

d

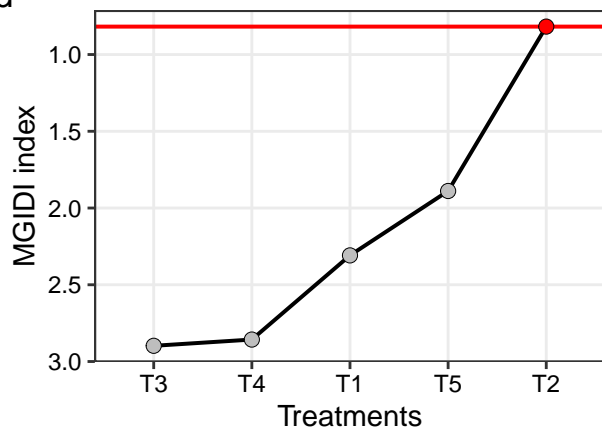

e

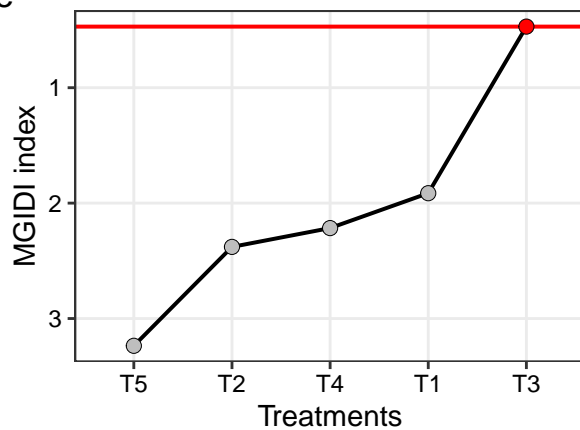

f

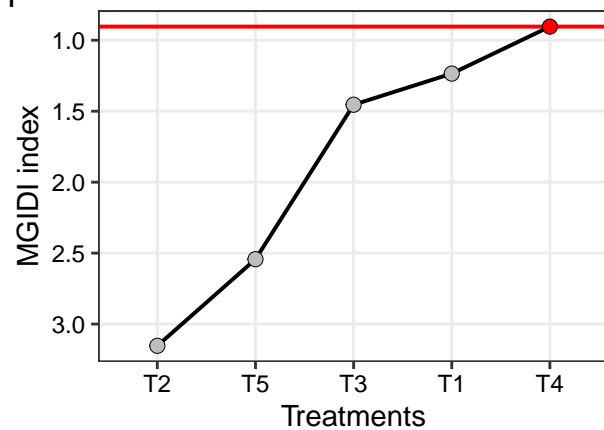

● Nonselected ● Selected

Supplement: Supplementary file 1 — Additional file 1. A website with the data, script, and results is available at https://tiagoolivoto.github.io/paper_mgidi_pm/. The source code used to produce the static website and the results in this manuscript have been archived at 10.5281/zenodo.7155173 as manuscript v2. [file 13007_2022_952_MOESM1_ESM.zip › TiagoOlivoto-paper_mgidi_pm-11ef6c1/docs/figs/fig1.pdf]

a

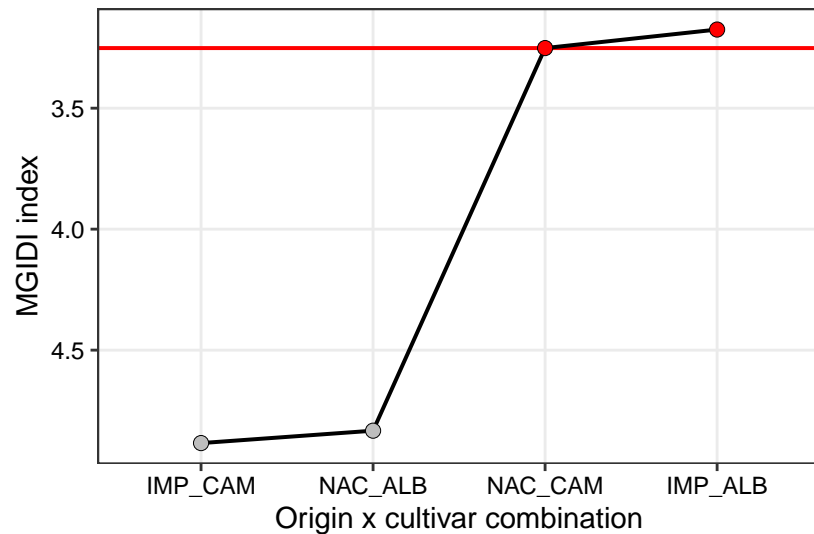

● Nonselected ● Selected

b

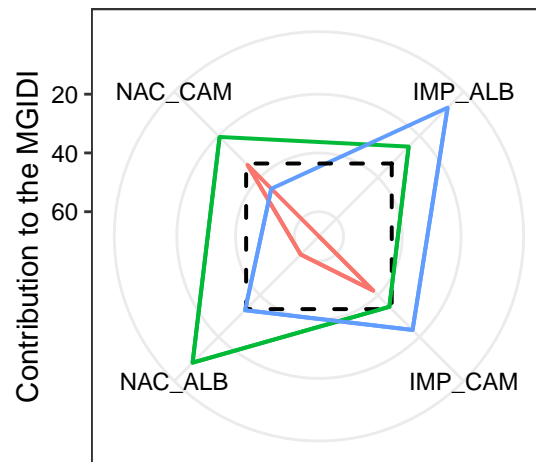

FA1 FA2 FA3

c

PCA – Biplot

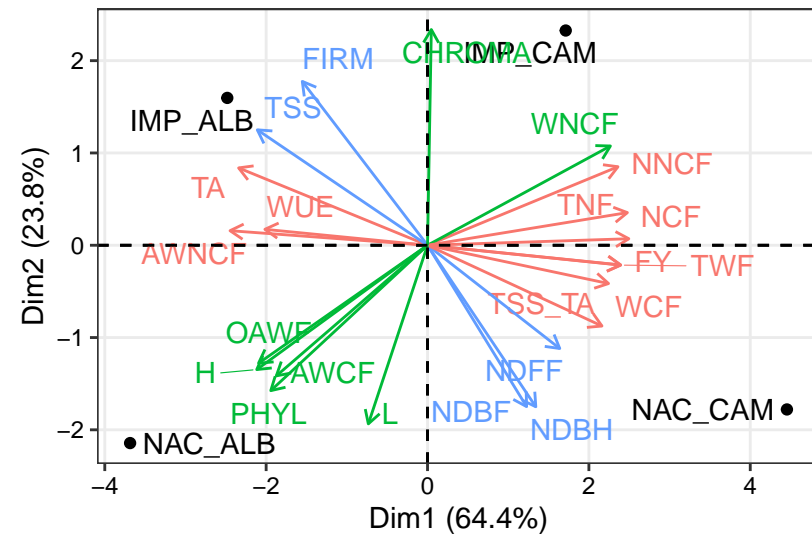

Supplement: Supplementary file 1 — Additional file 1. A website with the data, script, and results is available at https://tiagoolivoto.github.io/paper_mgidi_pm/. The source code used to produce the static website and the results in this manuscript have been archived at 10.5281/zenodo.7155173 as manuscript v2. [file 13007_2022_952_MOESM1_ESM.zip › TiagoOlivoto-paper_mgidi_pm-11ef6c1/docs/figs/fig2.pdf]

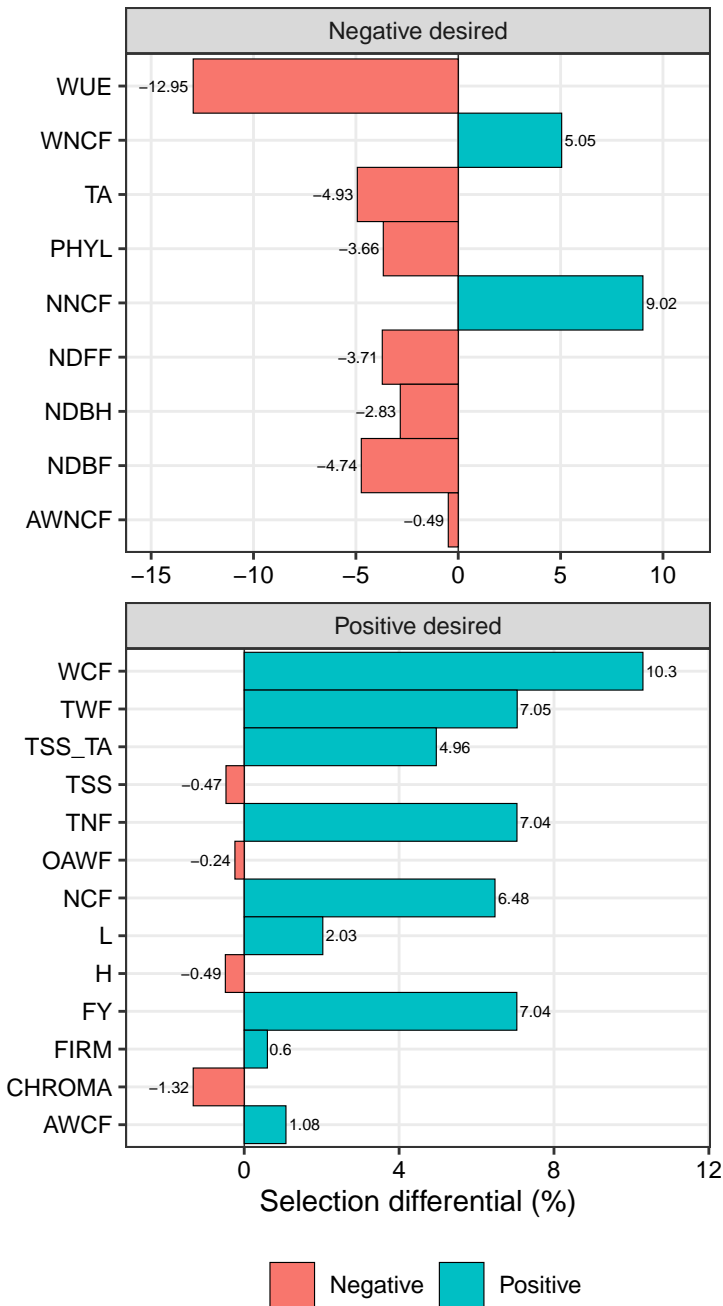

Supplement: Supplementary file 1 — Additional file 1. A website with the data, script, and results is available at https://tiagoolivoto.github.io/paper_mgidi_pm/. The source code used to produce the static website and the results in this manuscript have been archived at 10.5281/zenodo.7155173 as manuscript v2. [file 13007_2022_952_MOESM1_ESM.zip › TiagoOlivoto-paper_mgidi_pm-11ef6c1/docs/figs/fig3.pdf]

a

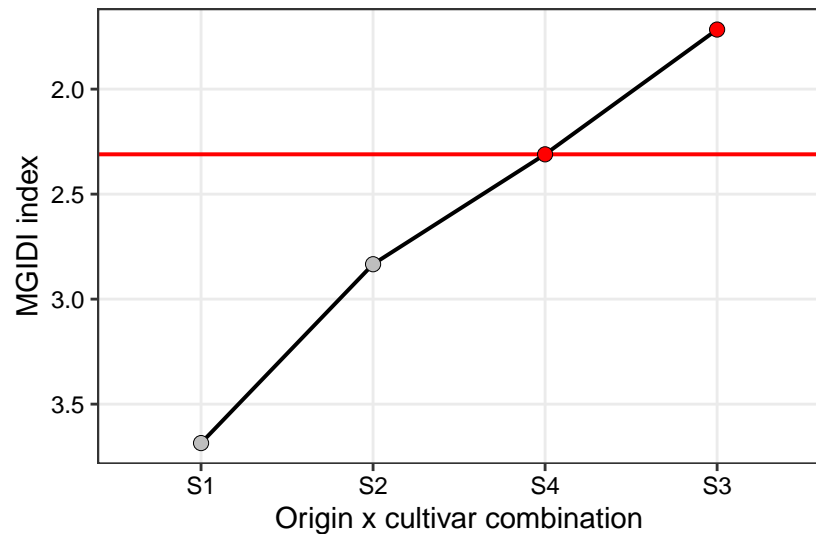

● Nonselected ● Selected

b

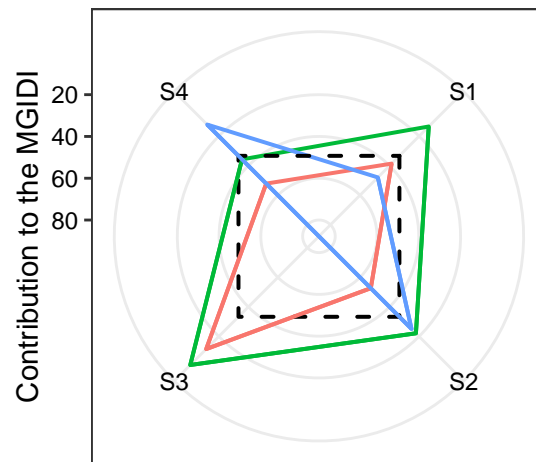

FA1 FA2 FA3

c

PCA – Biplot

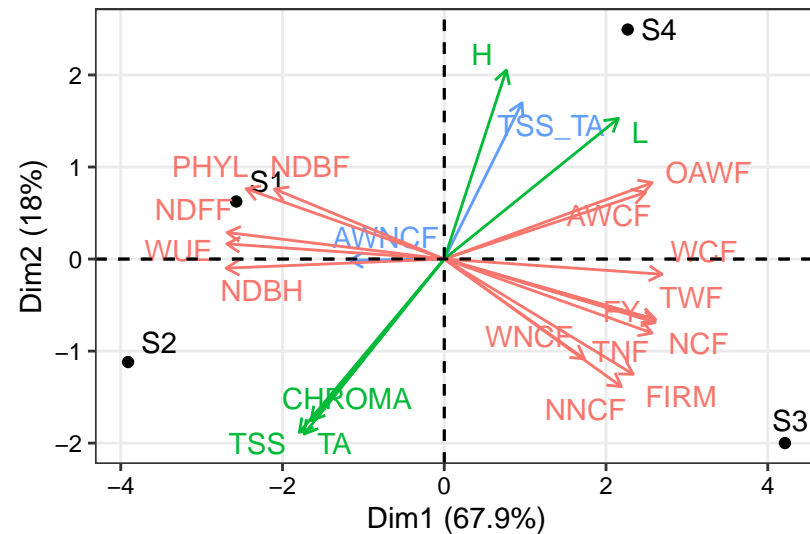

FA1 FA2 FA3

Supplement: Supplementary file 1 — Additional file 1. A website with the data, script, and results is available at https://tiagoolivoto.github.io/paper_mgidi_pm/. The source code used to produce the static website and the results in this manuscript have been archived at 10.5281/zenodo.7155173 as manuscript v2. [file 13007_2022_952_MOESM1_ESM.zip › TiagoOlivoto-paper_mgidi_pm-11ef6c1/docs/figs/fig4.pdf]

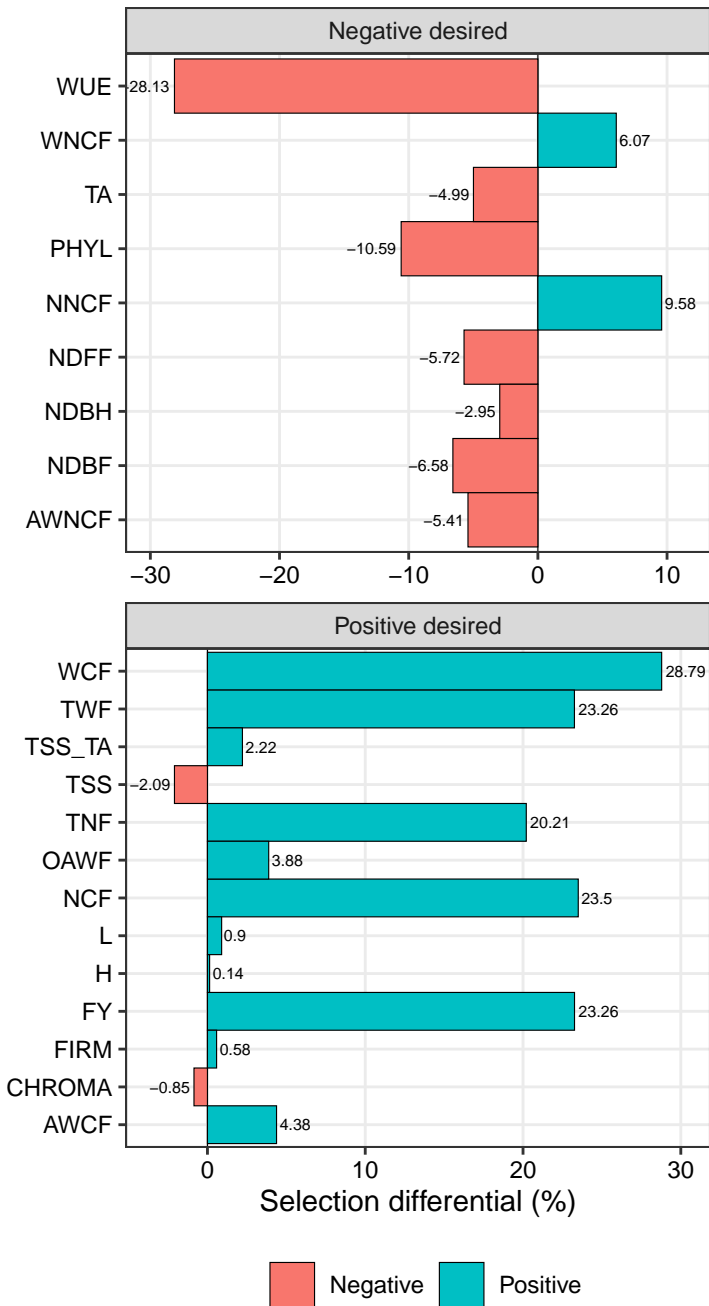

Supplement: Supplementary file 1 — Additional file 1. A website with the data, script, and results is available at https://tiagoolivoto.github.io/paper_mgidi_pm/. The source code used to produce the static website and the results in this manuscript have been archived at 10.5281/zenodo.7155173 as manuscript v2. [file 13007_2022_952_MOESM1_ESM.zip › TiagoOlivoto-paper_mgidi_pm-11ef6c1/docs/figs/fig5.pdf]

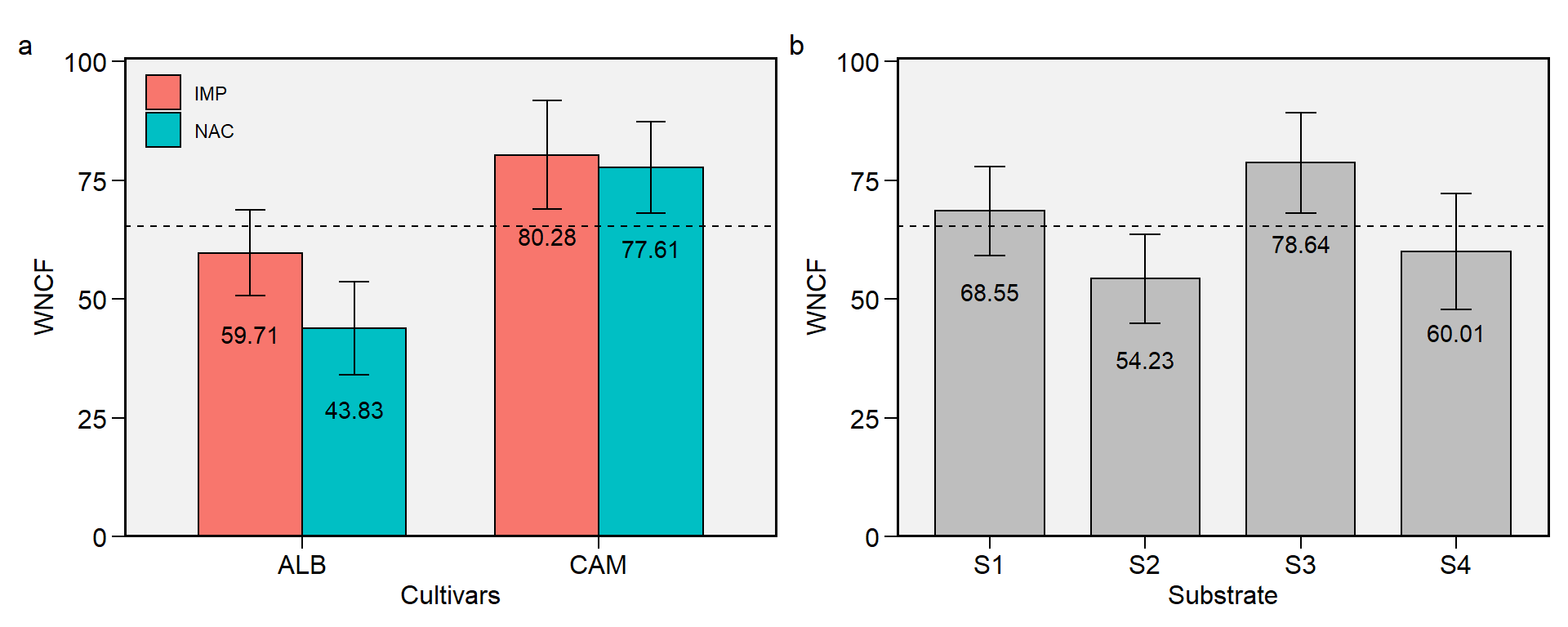

Supplement: Supplementary file 1 — Additional file 1. A website with the data, script, and results is available at https://tiagoolivoto.github.io/paper_mgidi_pm/. The source code used to produce the static website and the results in this manuscript have been archived at 10.5281/zenodo.7155173 as manuscript v2. [file 13007_2022_952_MOESM1_ESM.zip › TiagoOlivoto-paper_mgidi_pm-11ef6c1/docs/sup_figures_files/figure-html/unnamed-chunk-10-1.png]

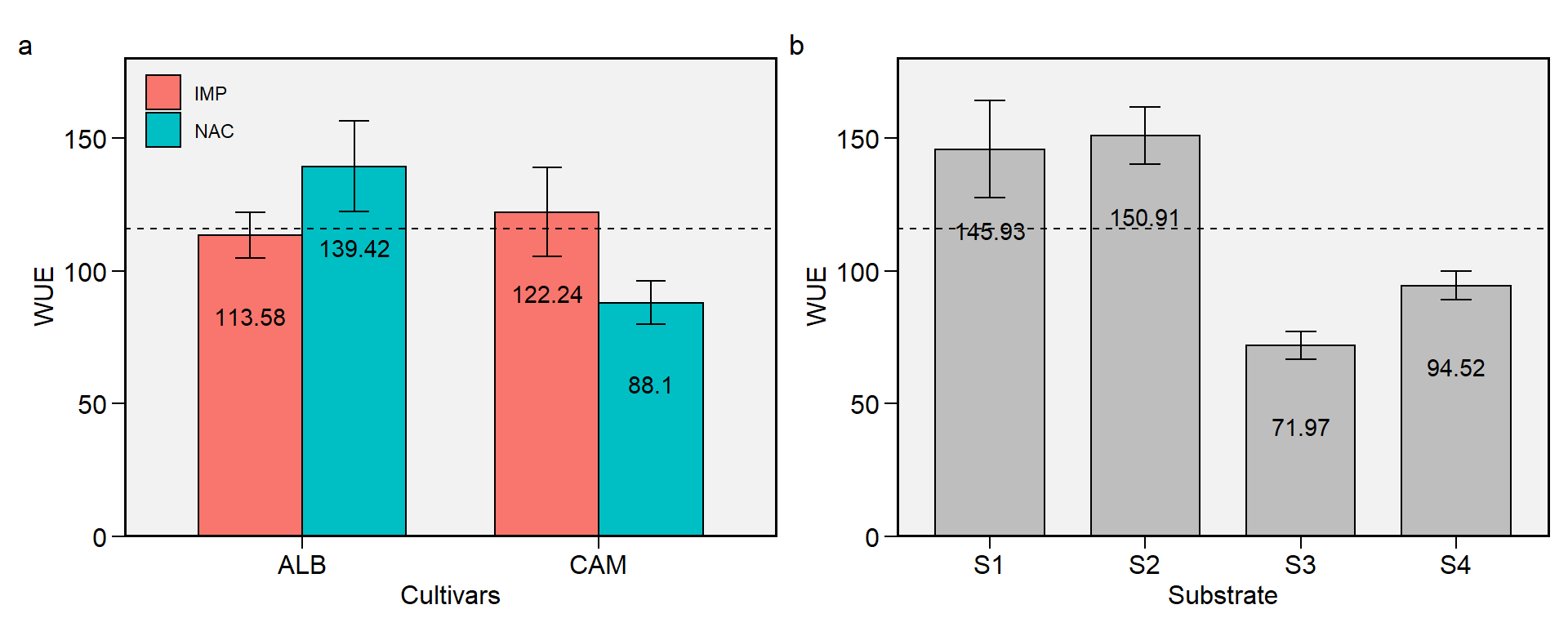

Supplement: Supplementary file 1 — Additional file 1. A website with the data, script, and results is available at https://tiagoolivoto.github.io/paper_mgidi_pm/. The source code used to produce the static website and the results in this manuscript have been archived at 10.5281/zenodo.7155173 as manuscript v2. [file 13007_2022_952_MOESM1_ESM.zip › TiagoOlivoto-paper_mgidi_pm-11ef6c1/docs/sup_figures_files/figure-html/unnamed-chunk-11-1.png]

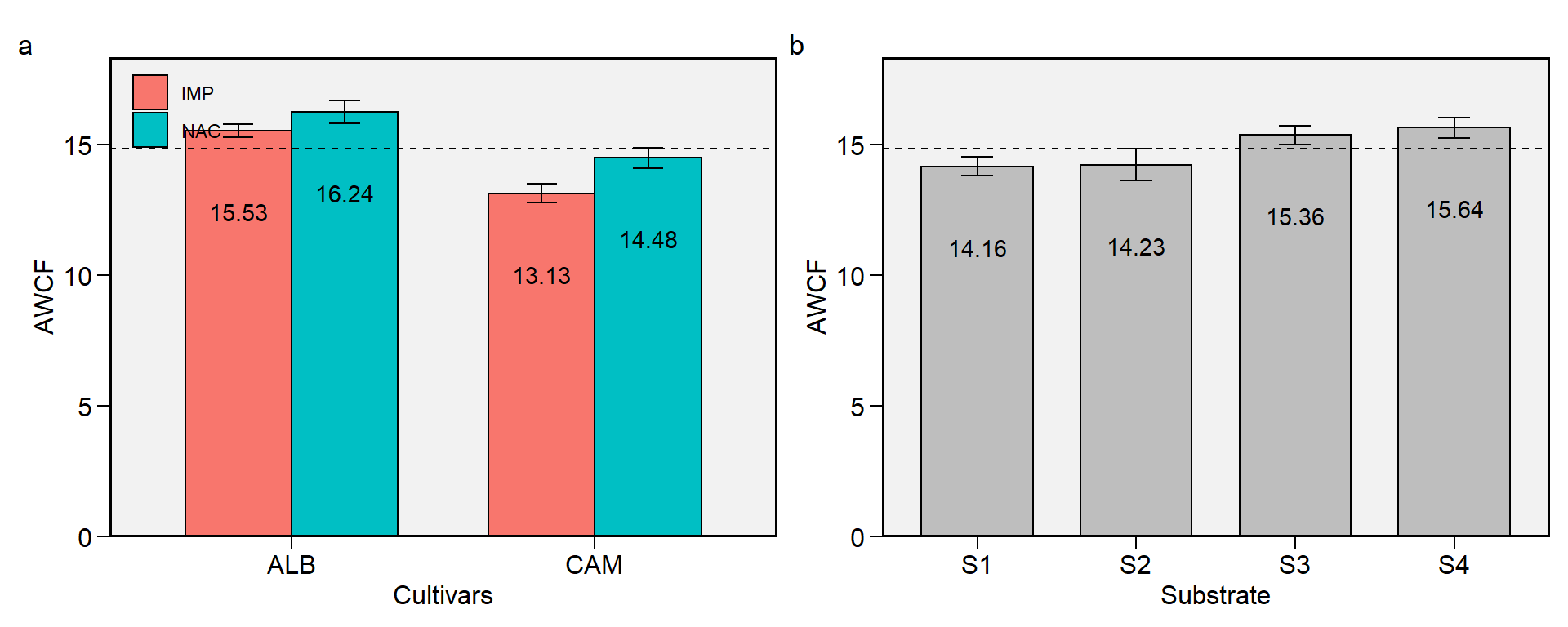

Supplement: Supplementary file 1 — Additional file 1. A website with the data, script, and results is available at https://tiagoolivoto.github.io/paper_mgidi_pm/. The source code used to produce the static website and the results in this manuscript have been archived at 10.5281/zenodo.7155173 as manuscript v2. [file 13007_2022_952_MOESM1_ESM.zip › TiagoOlivoto-paper_mgidi_pm-11ef6c1/docs/sup_figures_files/figure-html/unnamed-chunk-12-1.png]

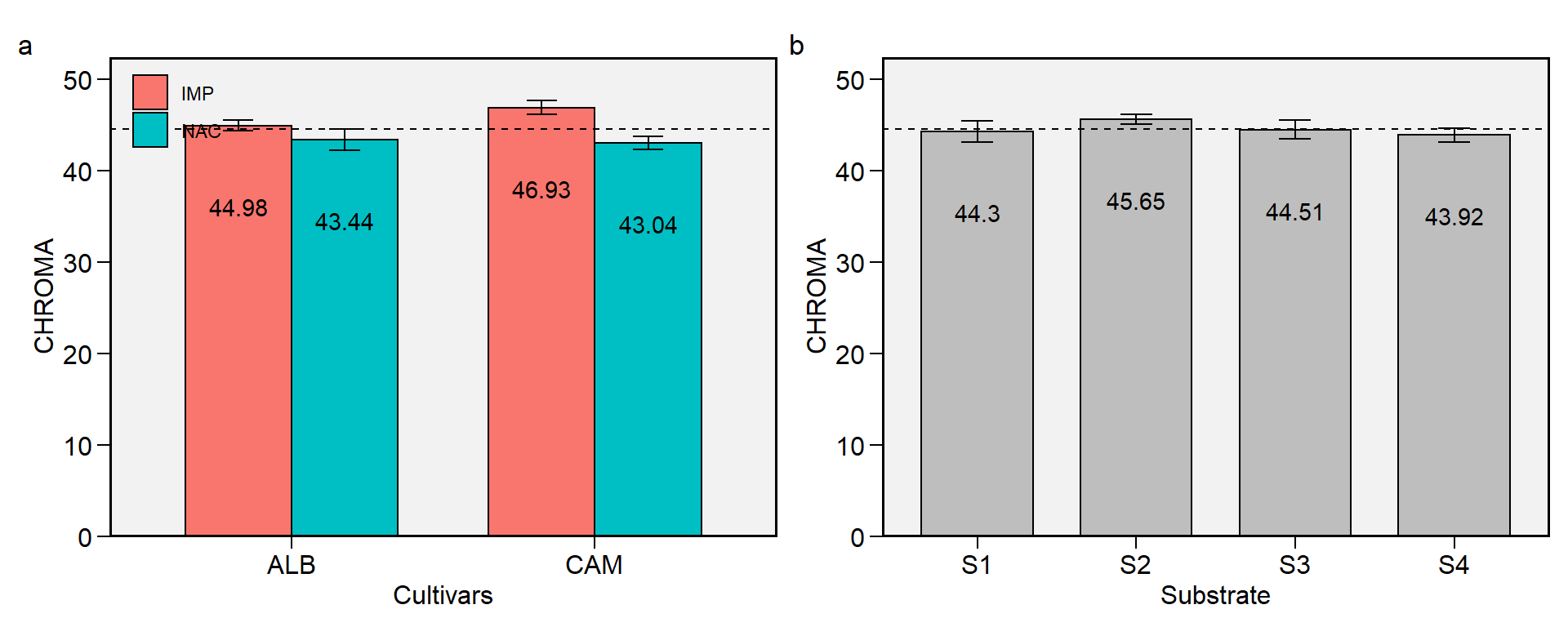

Supplement: Supplementary file 1 — Additional file 1. A website with the data, script, and results is available at https://tiagoolivoto.github.io/paper_mgidi_pm/. The source code used to produce the static website and the results in this manuscript have been archived at 10.5281/zenodo.7155173 as manuscript v2. [file 13007_2022_952_MOESM1_ESM.zip › TiagoOlivoto-paper_mgidi_pm-11ef6c1/docs/sup_figures_files/figure-html/unnamed-chunk-13-1.png]

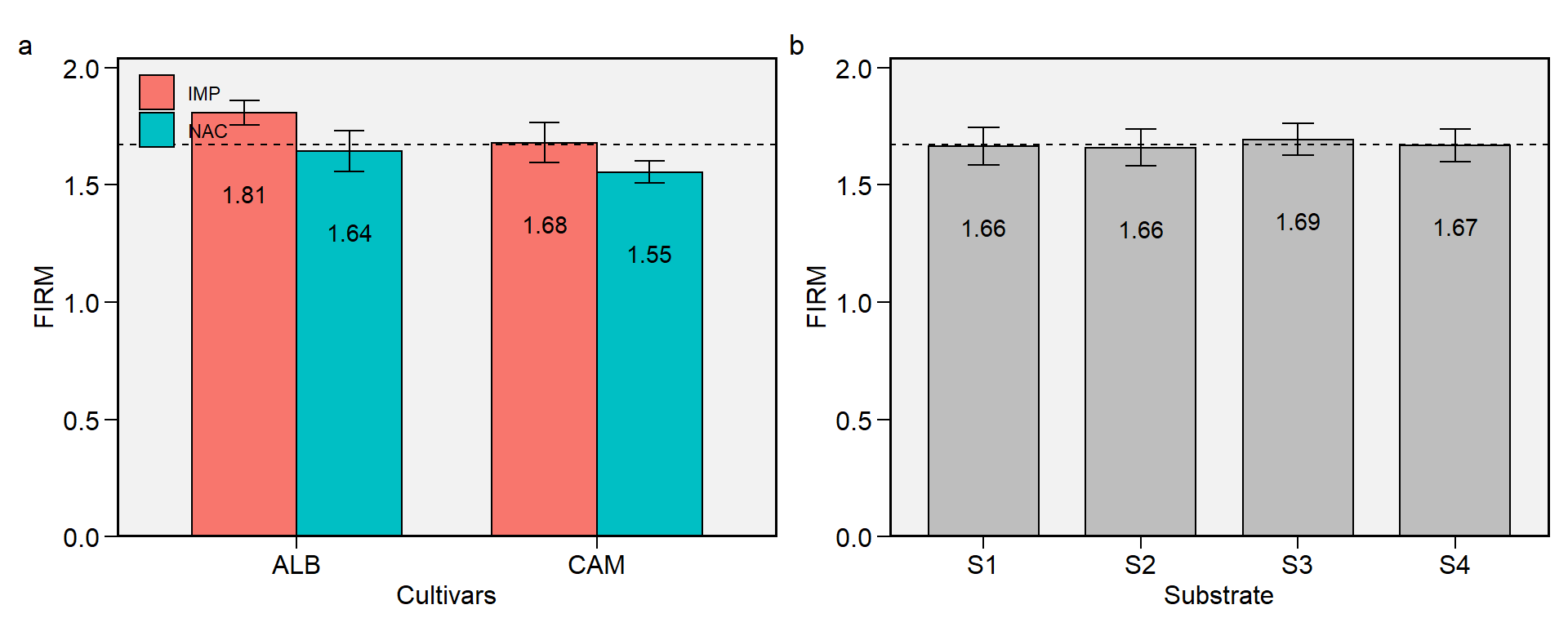

Supplement: Supplementary file 1 — Additional file 1. A website with the data, script, and results is available at https://tiagoolivoto.github.io/paper_mgidi_pm/. The source code used to produce the static website and the results in this manuscript have been archived at 10.5281/zenodo.7155173 as manuscript v2. [file 13007_2022_952_MOESM1_ESM.zip › TiagoOlivoto-paper_mgidi_pm-11ef6c1/docs/sup_figures_files/figure-html/unnamed-chunk-14-1.png]

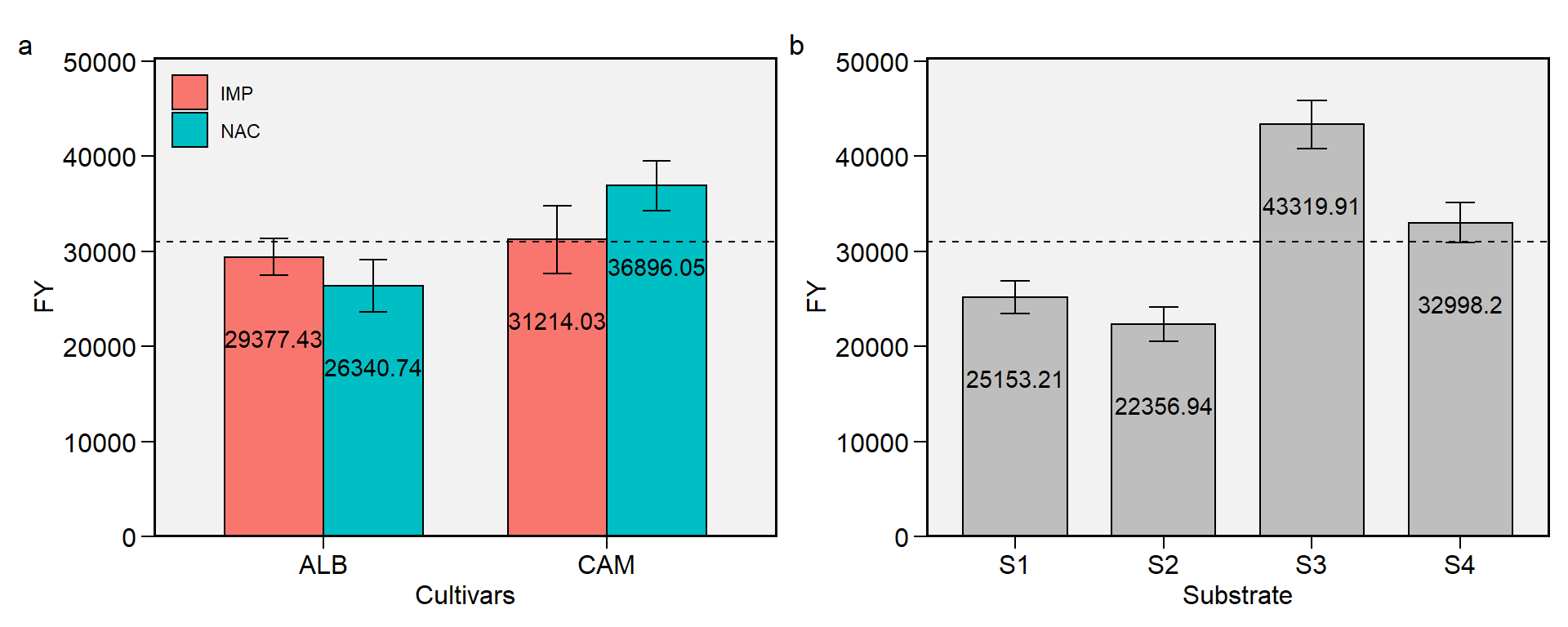

Supplement: Supplementary file 1 — Additional file 1. A website with the data, script, and results is available at https://tiagoolivoto.github.io/paper_mgidi_pm/. The source code used to produce the static website and the results in this manuscript have been archived at 10.5281/zenodo.7155173 as manuscript v2. [file 13007_2022_952_MOESM1_ESM.zip › TiagoOlivoto-paper_mgidi_pm-11ef6c1/docs/sup_figures_files/figure-html/unnamed-chunk-15-1.png]

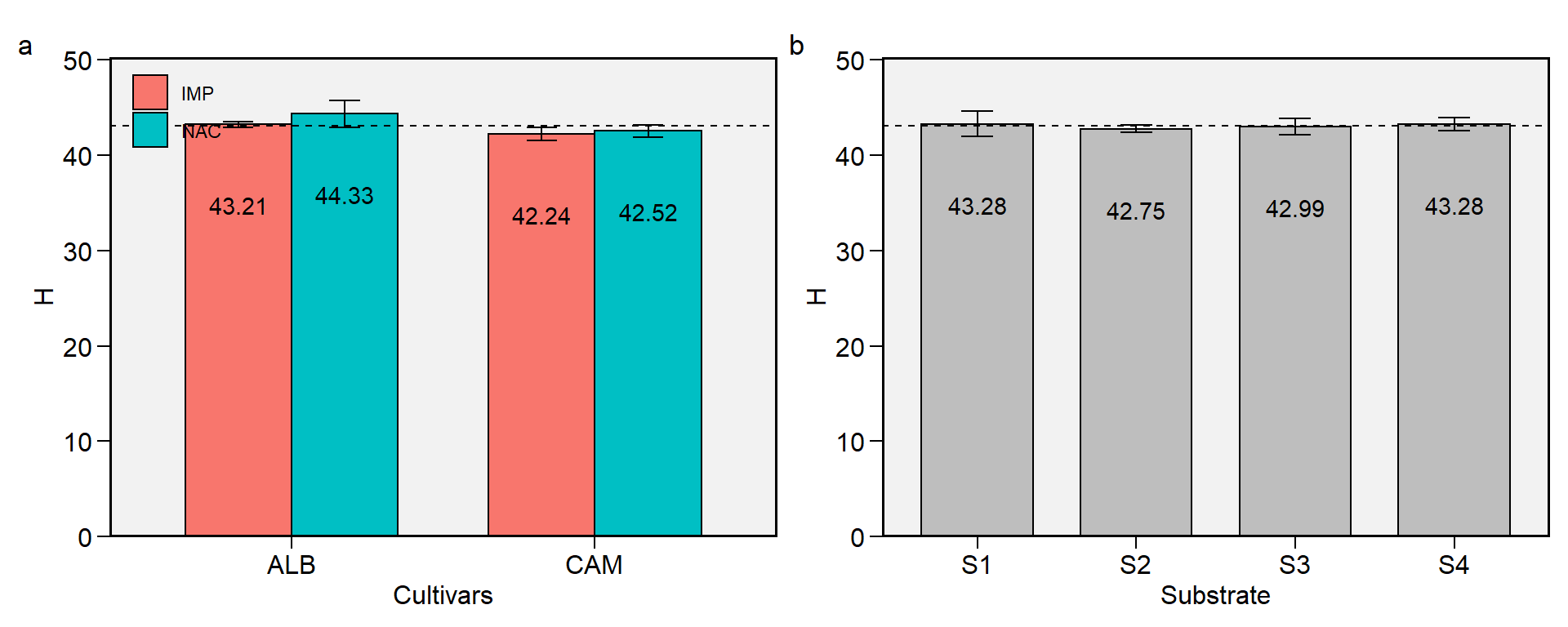

Supplement: Supplementary file 1 — Additional file 1. A website with the data, script, and results is available at https://tiagoolivoto.github.io/paper_mgidi_pm/. The source code used to produce the static website and the results in this manuscript have been archived at 10.5281/zenodo.7155173 as manuscript v2. [file 13007_2022_952_MOESM1_ESM.zip › TiagoOlivoto-paper_mgidi_pm-11ef6c1/docs/sup_figures_files/figure-html/unnamed-chunk-16-1.png]

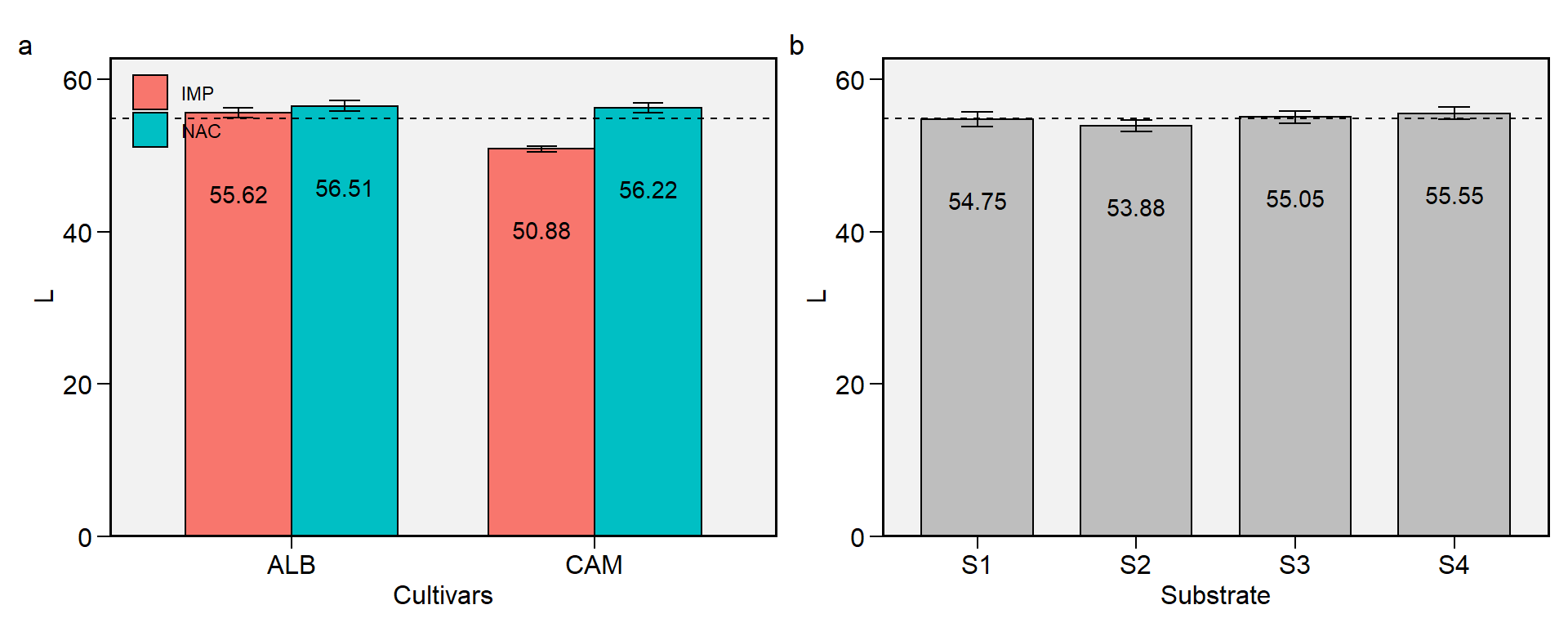

Supplement: Supplementary file 1 — Additional file 1. A website with the data, script, and results is available at https://tiagoolivoto.github.io/paper_mgidi_pm/. The source code used to produce the static website and the results in this manuscript have been archived at 10.5281/zenodo.7155173 as manuscript v2. [file 13007_2022_952_MOESM1_ESM.zip › TiagoOlivoto-paper_mgidi_pm-11ef6c1/docs/sup_figures_files/figure-html/unnamed-chunk-17-1.png]

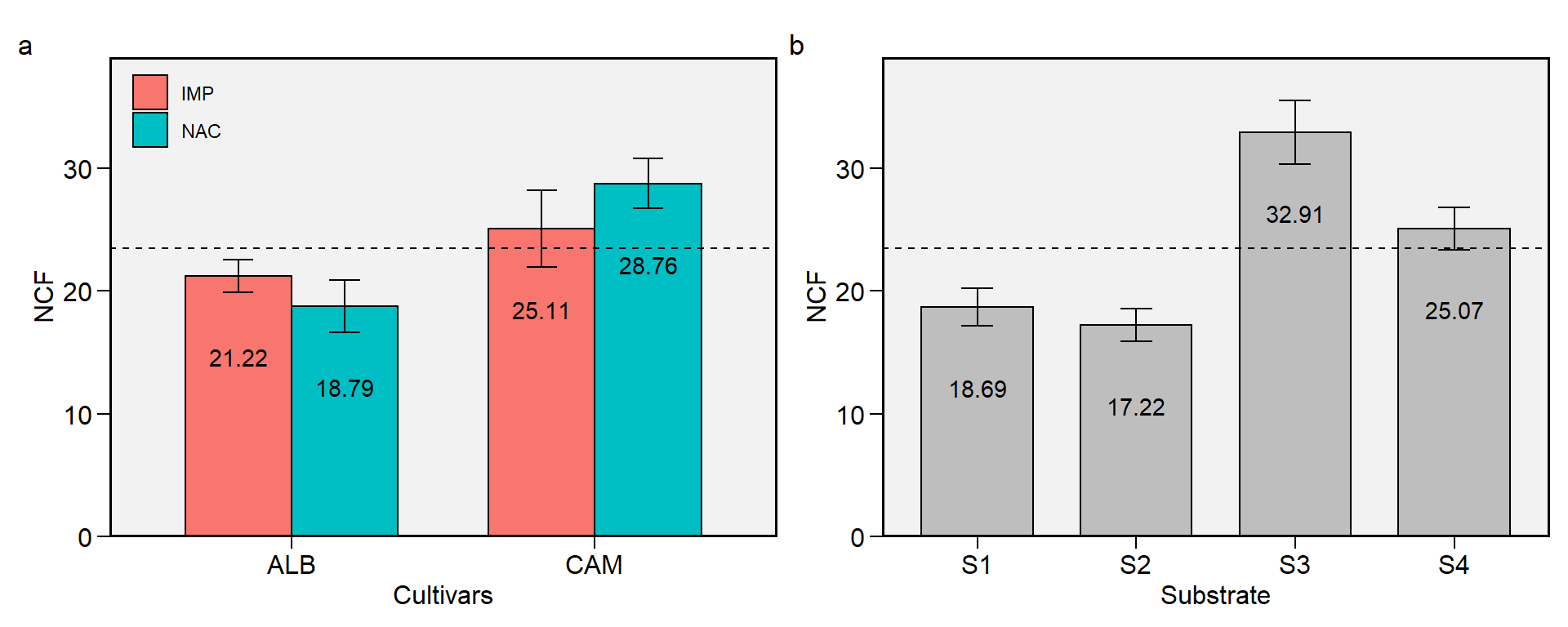

Supplement: Supplementary file 1 — Additional file 1. A website with the data, script, and results is available at https://tiagoolivoto.github.io/paper_mgidi_pm/. The source code used to produce the static website and the results in this manuscript have been archived at 10.5281/zenodo.7155173 as manuscript v2. [file 13007_2022_952_MOESM1_ESM.zip › TiagoOlivoto-paper_mgidi_pm-11ef6c1/docs/sup_figures_files/figure-html/unnamed-chunk-18-1.png]

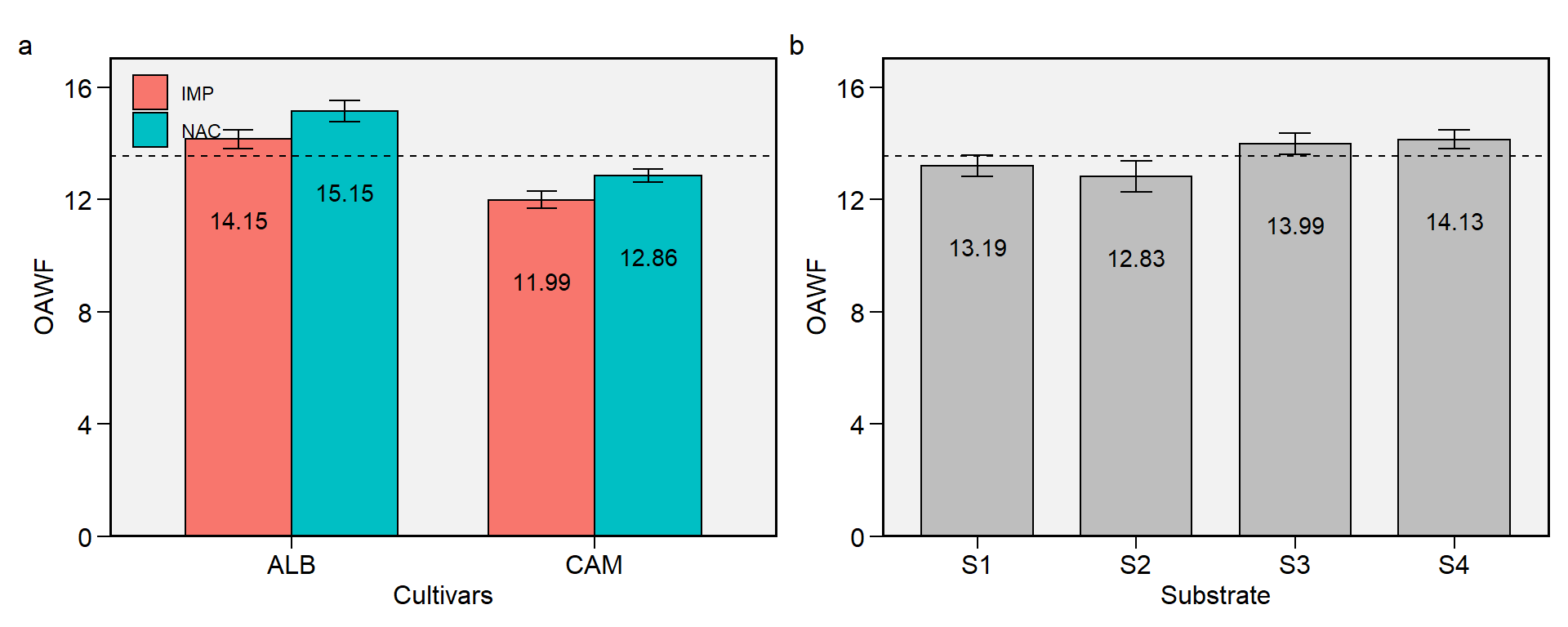

Supplement: Supplementary file 1 — Additional file 1. A website with the data, script, and results is available at https://tiagoolivoto.github.io/paper_mgidi_pm/. The source code used to produce the static website and the results in this manuscript have been archived at 10.5281/zenodo.7155173 as manuscript v2. [file 13007_2022_952_MOESM1_ESM.zip › TiagoOlivoto-paper_mgidi_pm-11ef6c1/docs/sup_figures_files/figure-html/unnamed-chunk-19-1.png]

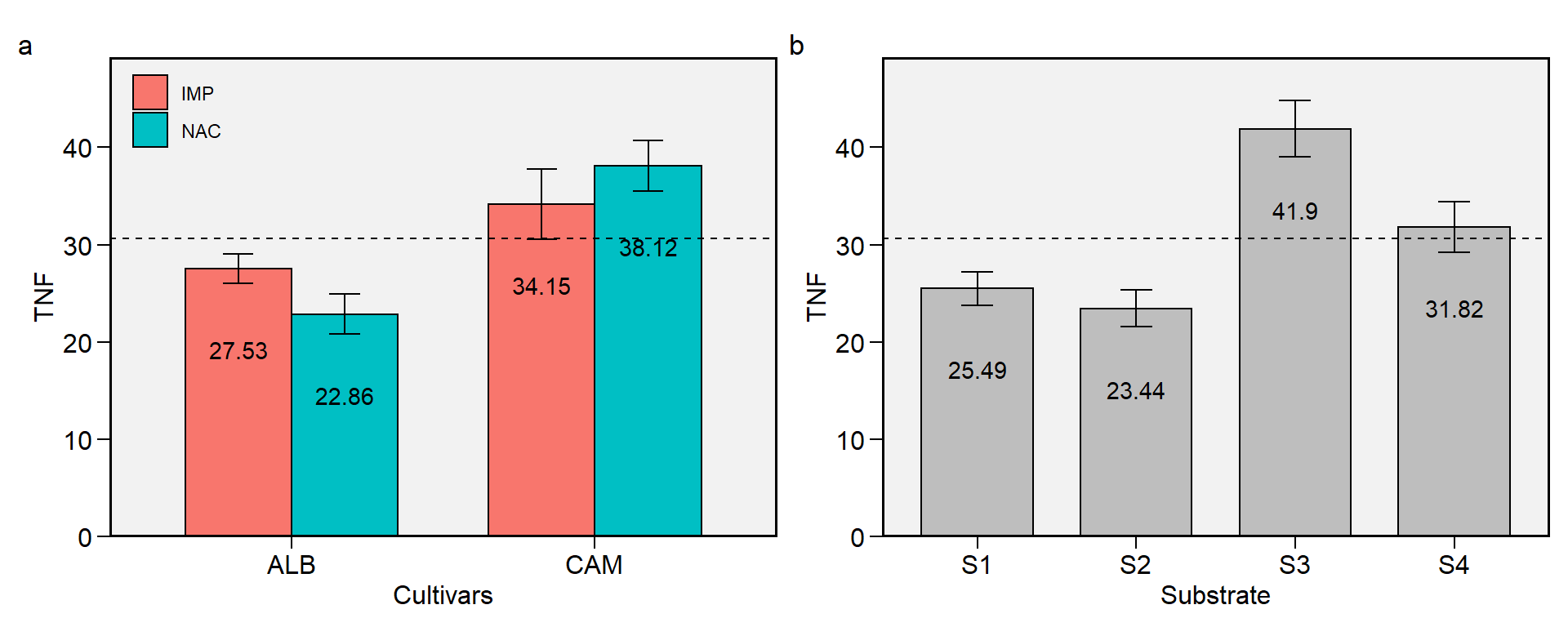

Supplement: Supplementary file 1 — Additional file 1. A website with the data, script, and results is available at https://tiagoolivoto.github.io/paper_mgidi_pm/. The source code used to produce the static website and the results in this manuscript have been archived at 10.5281/zenodo.7155173 as manuscript v2. [file 13007_2022_952_MOESM1_ESM.zip › TiagoOlivoto-paper_mgidi_pm-11ef6c1/docs/sup_figures_files/figure-html/unnamed-chunk-20-1.png]

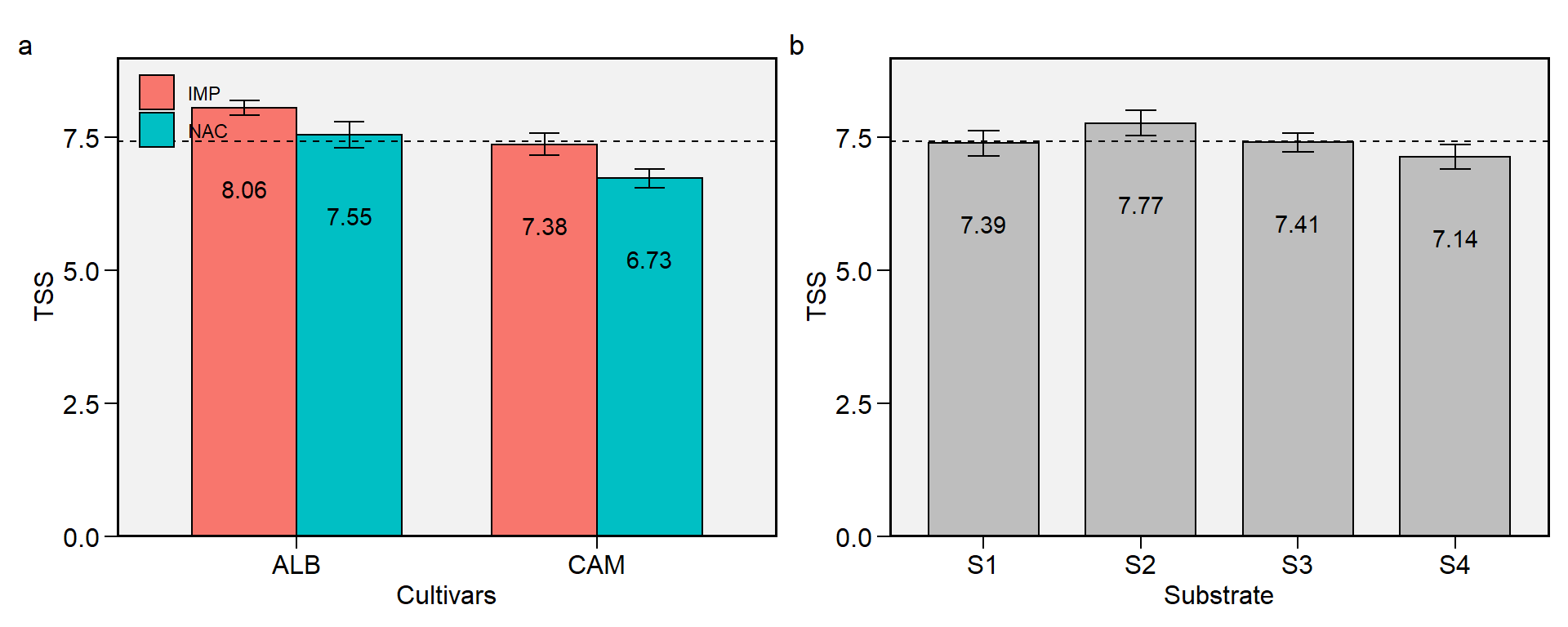

Supplement: Supplementary file 1 — Additional file 1. A website with the data, script, and results is available at https://tiagoolivoto.github.io/paper_mgidi_pm/. The source code used to produce the static website and the results in this manuscript have been archived at 10.5281/zenodo.7155173 as manuscript v2. [file 13007_2022_952_MOESM1_ESM.zip › TiagoOlivoto-paper_mgidi_pm-11ef6c1/docs/sup_figures_files/figure-html/unnamed-chunk-21-1.png]

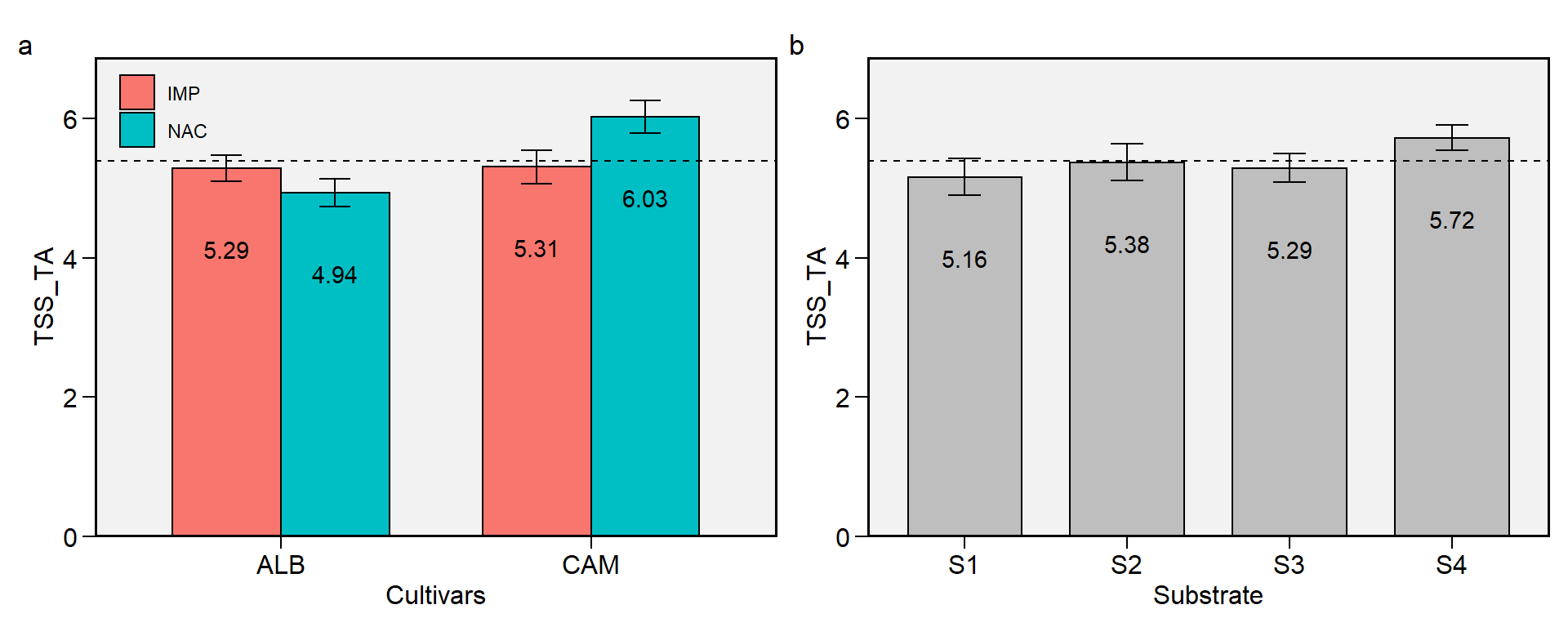

Supplement: Supplementary file 1 — Additional file 1. A website with the data, script, and results is available at https://tiagoolivoto.github.io/paper_mgidi_pm/. The source code used to produce the static website and the results in this manuscript have been archived at 10.5281/zenodo.7155173 as manuscript v2. [file 13007_2022_952_MOESM1_ESM.zip › TiagoOlivoto-paper_mgidi_pm-11ef6c1/docs/sup_figures_files/figure-html/unnamed-chunk-22-1.png]

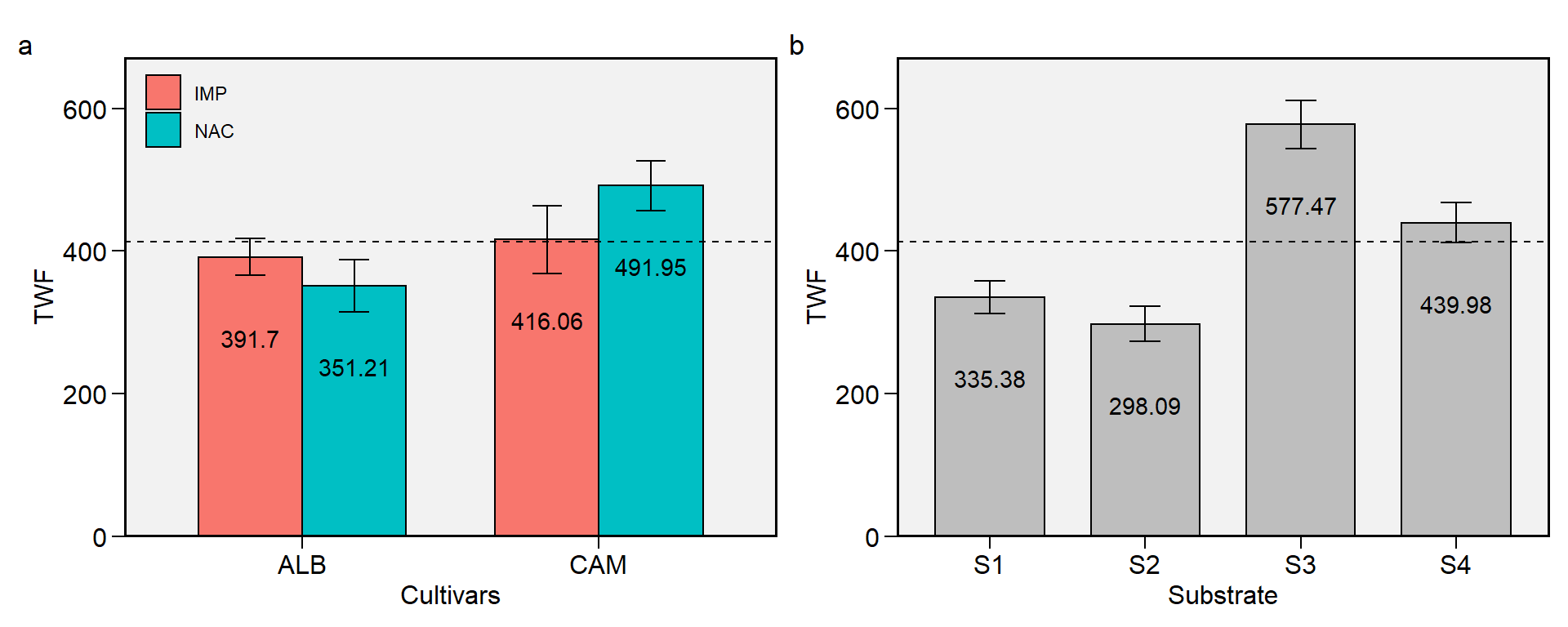

Supplement: Supplementary file 1 — Additional file 1. A website with the data, script, and results is available at https://tiagoolivoto.github.io/paper_mgidi_pm/. The source code used to produce the static website and the results in this manuscript have been archived at 10.5281/zenodo.7155173 as manuscript v2. [file 13007_2022_952_MOESM1_ESM.zip › TiagoOlivoto-paper_mgidi_pm-11ef6c1/docs/sup_figures_files/figure-html/unnamed-chunk-23-1.png]

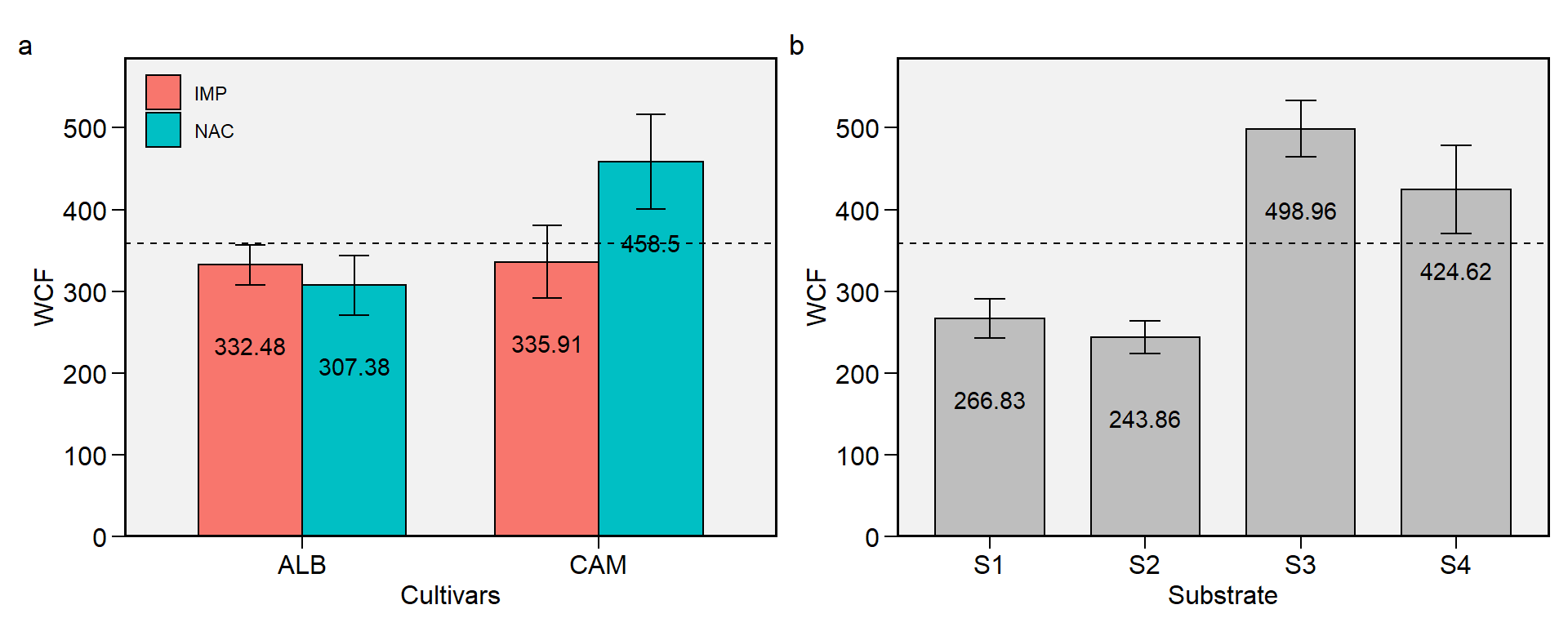

Supplement: Supplementary file 1 — Additional file 1. A website with the data, script, and results is available at https://tiagoolivoto.github.io/paper_mgidi_pm/. The source code used to produce the static website and the results in this manuscript have been archived at 10.5281/zenodo.7155173 as manuscript v2. [file 13007_2022_952_MOESM1_ESM.zip › TiagoOlivoto-paper_mgidi_pm-11ef6c1/docs/sup_figures_files/figure-html/unnamed-chunk-24-1.png]

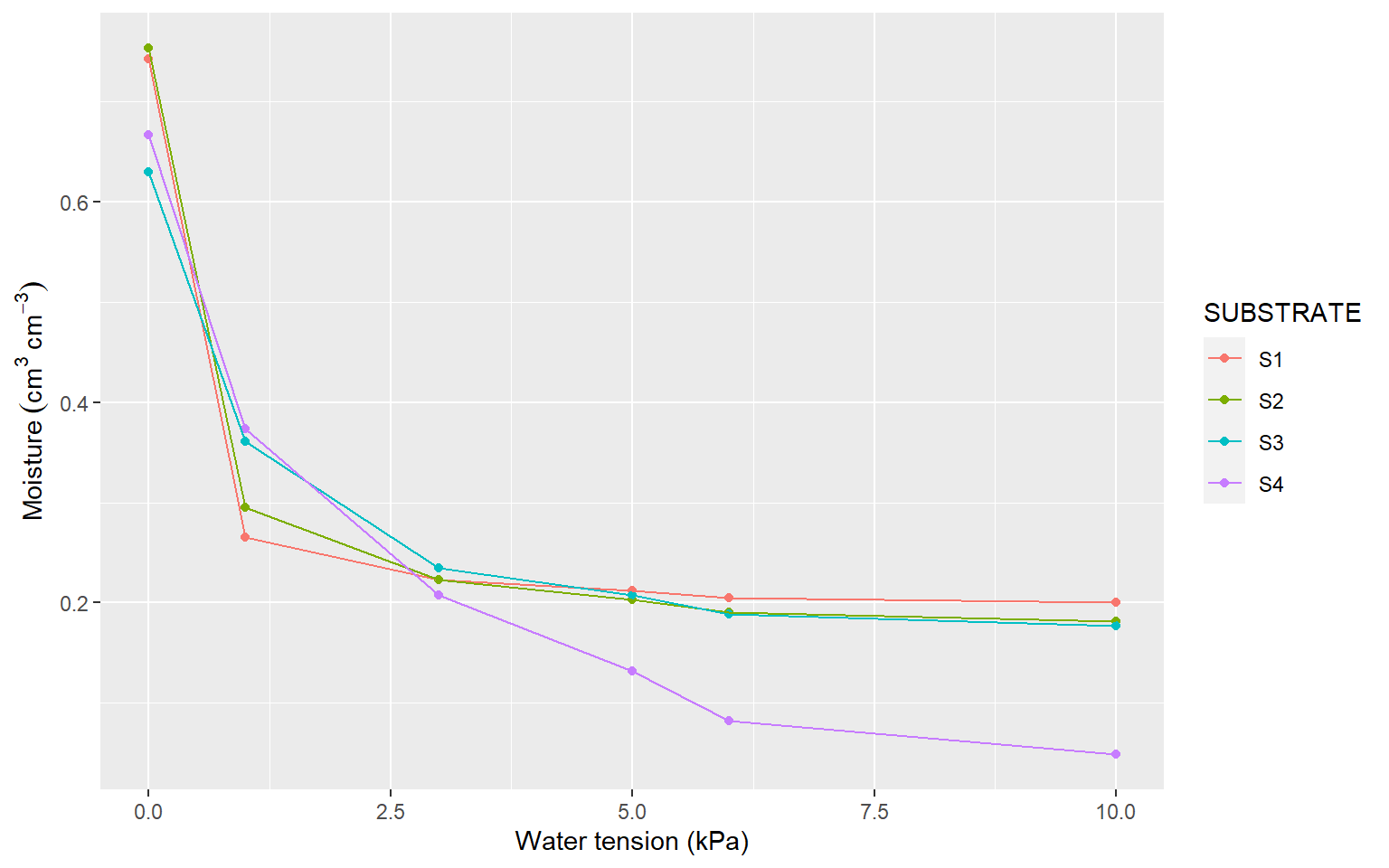

Supplement: Supplementary file 1 — Additional file 1. A website with the data, script, and results is available at https://tiagoolivoto.github.io/paper_mgidi_pm/. The source code used to produce the static website and the results in this manuscript have been archived at 10.5281/zenodo.7155173 as manuscript v2. [file 13007_2022_952_MOESM1_ESM.zip › TiagoOlivoto-paper_mgidi_pm-11ef6c1/docs/sup_figures_files/figure-html/unnamed-chunk-25-1.png]

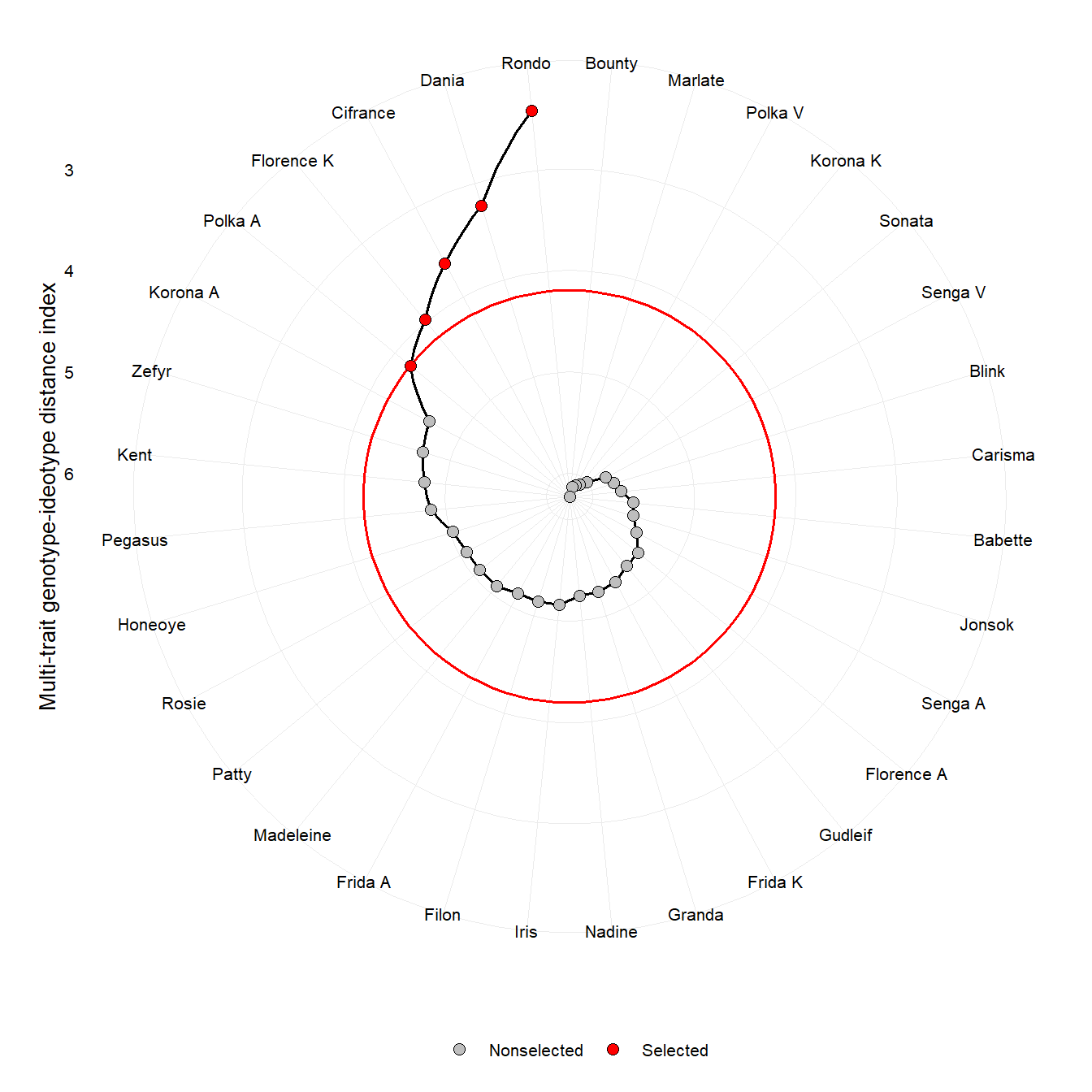

Supplement: Supplementary file 1 — Additional file 1. A website with the data, script, and results is available at https://tiagoolivoto.github.io/paper_mgidi_pm/. The source code used to produce the static website and the results in this manuscript have been archived at 10.5281/zenodo.7155173 as manuscript v2. [file 13007_2022_952_MOESM1_ESM.zip › TiagoOlivoto-paper_mgidi_pm-11ef6c1/docs/sup_figures_files/figure-html/unnamed-chunk-26-1.png]

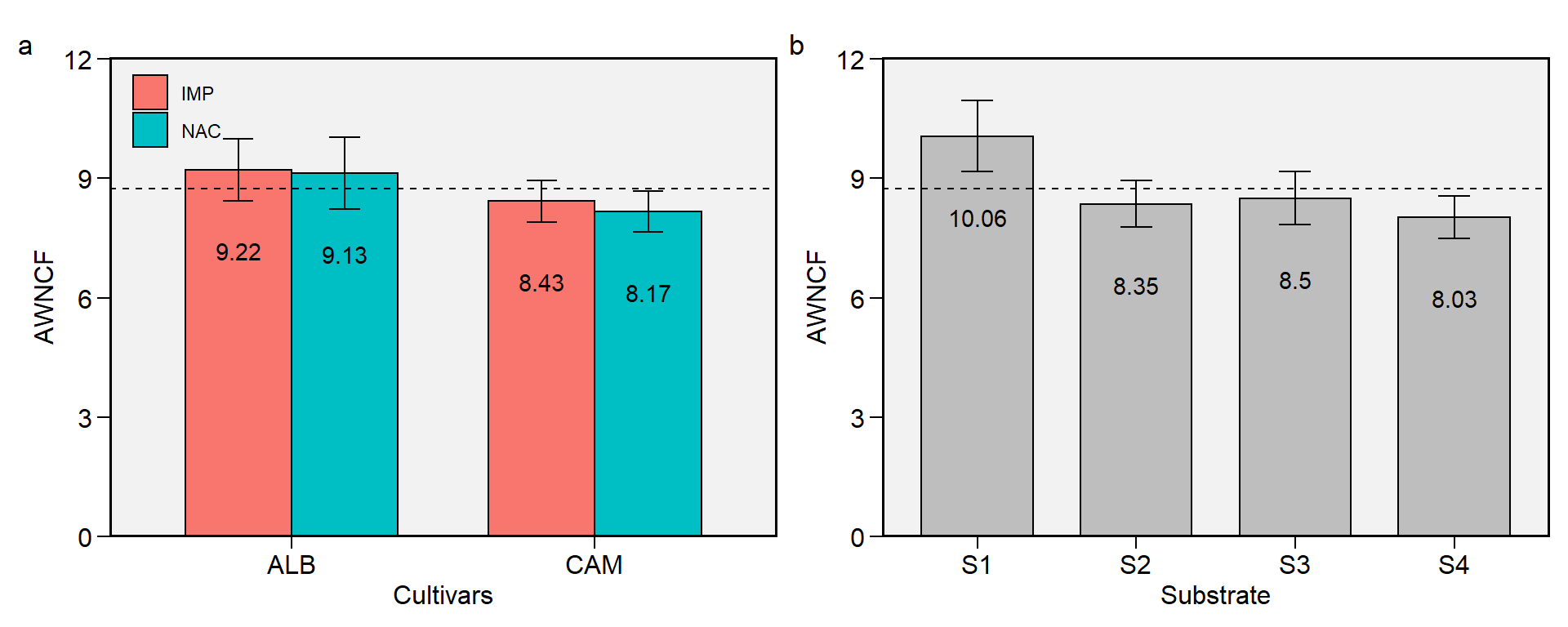

Supplement: Supplementary file 1 — Additional file 1. A website with the data, script, and results is available at https://tiagoolivoto.github.io/paper_mgidi_pm/. The source code used to produce the static website and the results in this manuscript have been archived at 10.5281/zenodo.7155173 as manuscript v2. [file 13007_2022_952_MOESM1_ESM.zip › TiagoOlivoto-paper_mgidi_pm-11ef6c1/docs/sup_figures_files/figure-html/unnamed-chunk-3-1.png]

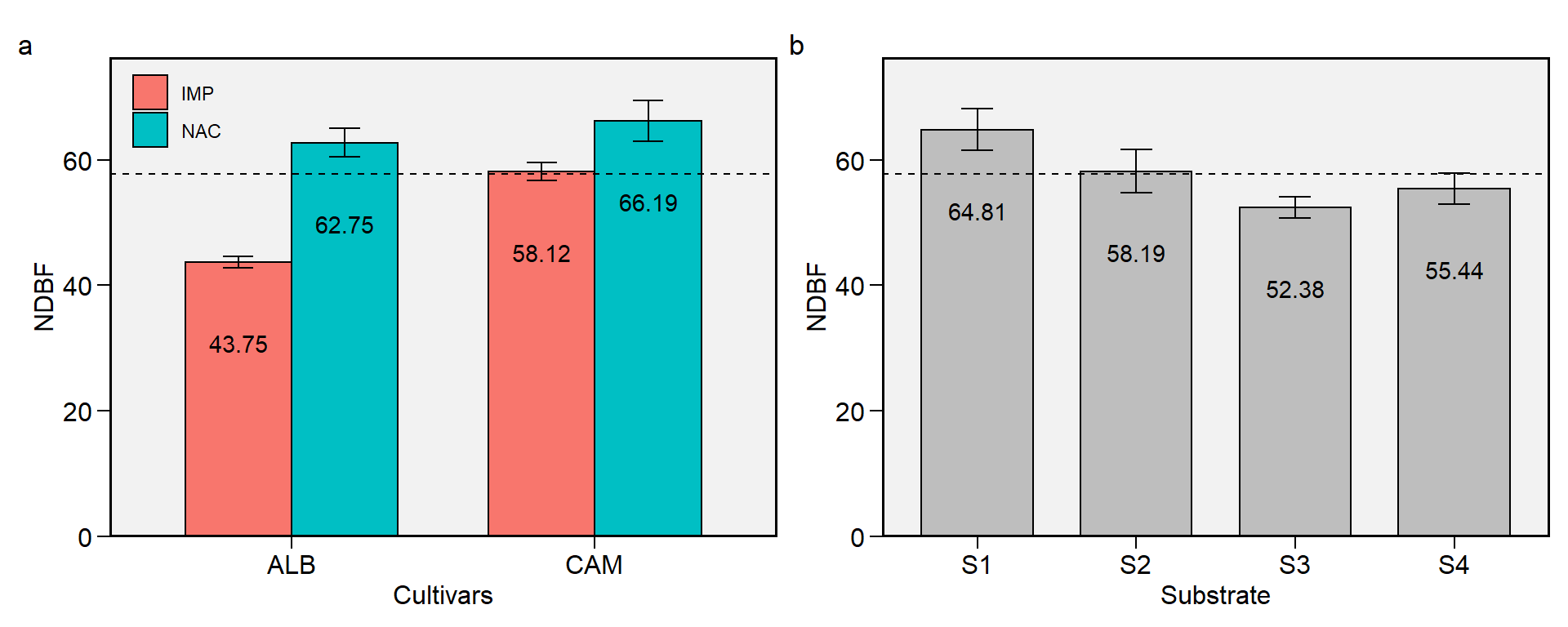

Supplement: Supplementary file 1 — Additional file 1. A website with the data, script, and results is available at https://tiagoolivoto.github.io/paper_mgidi_pm/. The source code used to produce the static website and the results in this manuscript have been archived at 10.5281/zenodo.7155173 as manuscript v2. [file 13007_2022_952_MOESM1_ESM.zip › TiagoOlivoto-paper_mgidi_pm-11ef6c1/docs/sup_figures_files/figure-html/unnamed-chunk-4-1.png]

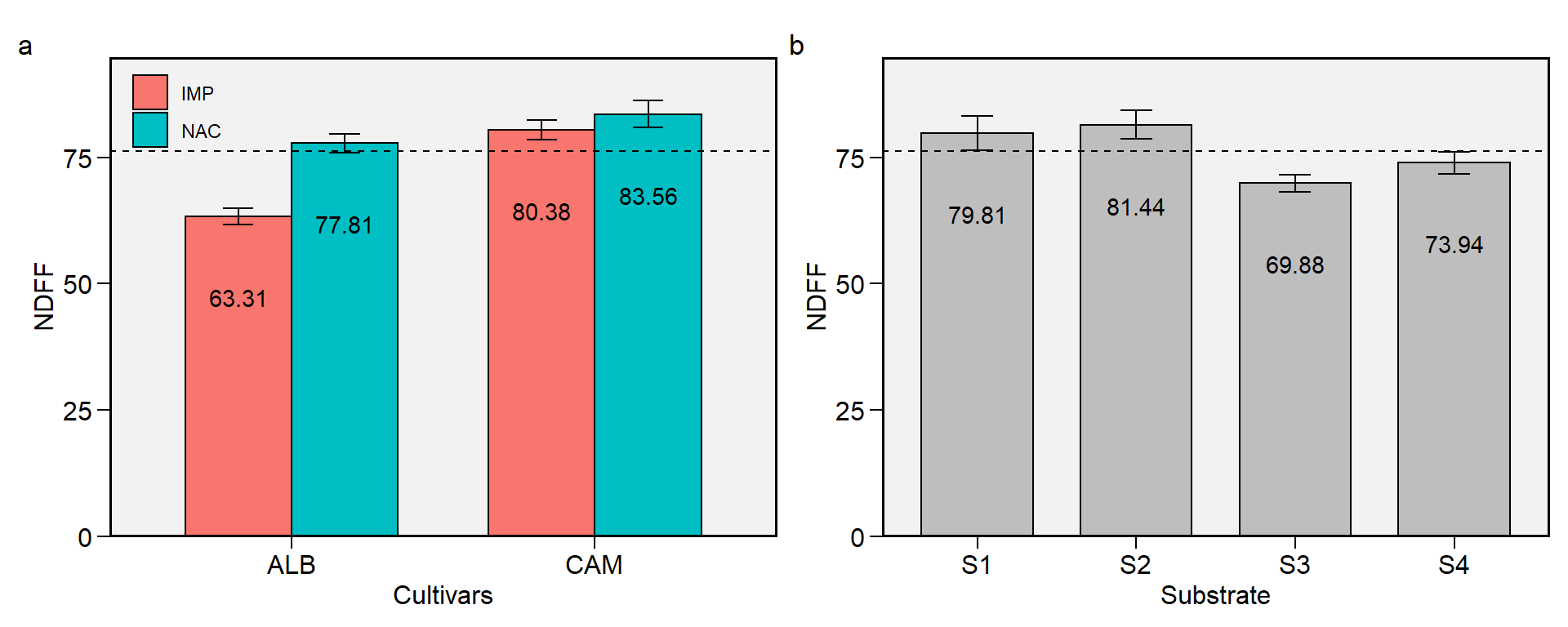

Supplement: Supplementary file 1 — Additional file 1. A website with the data, script, and results is available at https://tiagoolivoto.github.io/paper_mgidi_pm/. The source code used to produce the static website and the results in this manuscript have been archived at 10.5281/zenodo.7155173 as manuscript v2. [file 13007_2022_952_MOESM1_ESM.zip › TiagoOlivoto-paper_mgidi_pm-11ef6c1/docs/sup_figures_files/figure-html/unnamed-chunk-5-1.png]

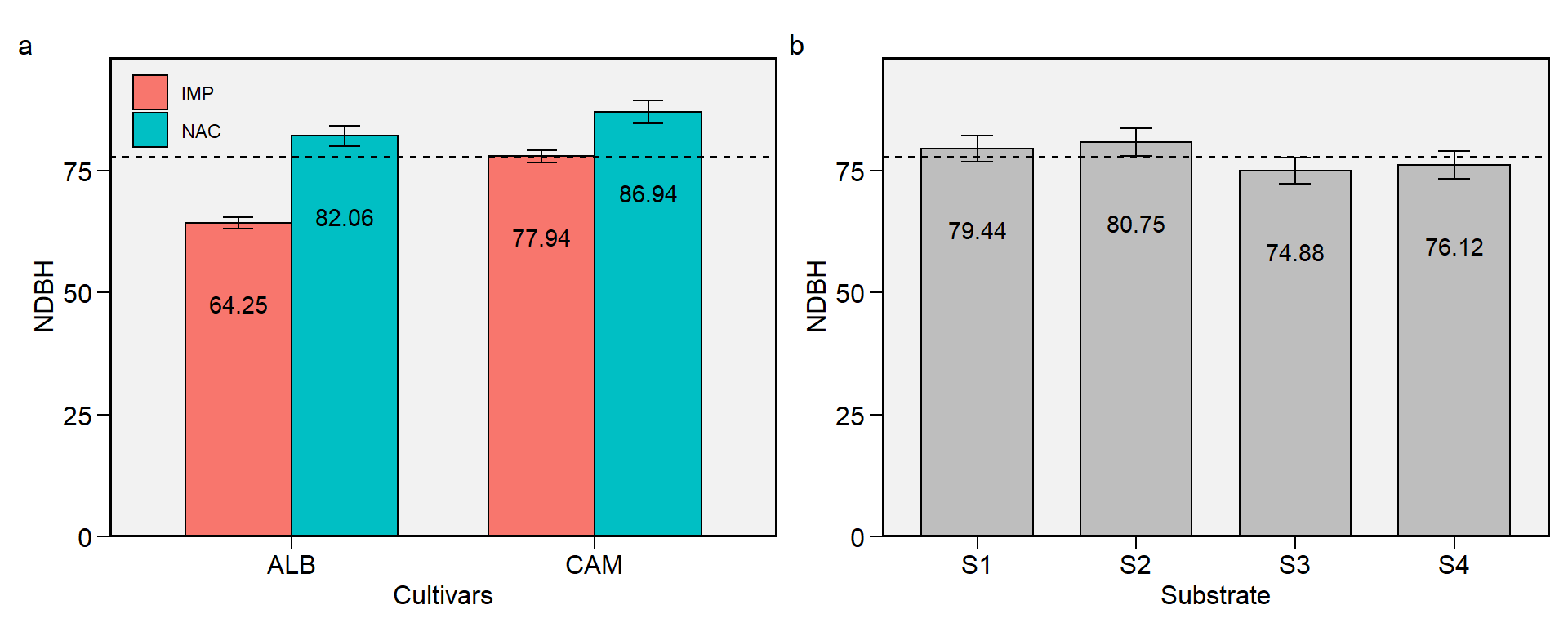

Supplement: Supplementary file 1 — Additional file 1. A website with the data, script, and results is available at https://tiagoolivoto.github.io/paper_mgidi_pm/. The source code used to produce the static website and the results in this manuscript have been archived at 10.5281/zenodo.7155173 as manuscript v2. [file 13007_2022_952_MOESM1_ESM.zip › TiagoOlivoto-paper_mgidi_pm-11ef6c1/docs/sup_figures_files/figure-html/unnamed-chunk-6-1.png]

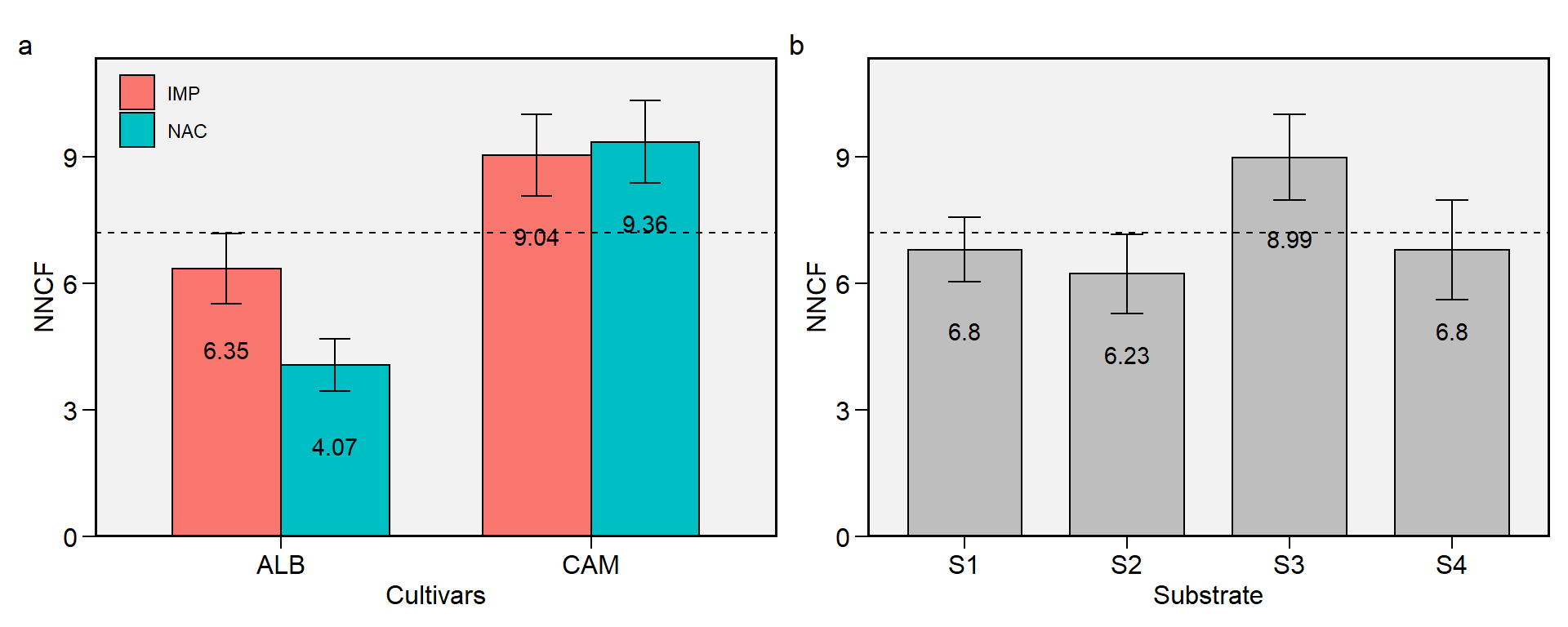

Supplement: Supplementary file 1 — Additional file 1. A website with the data, script, and results is available at https://tiagoolivoto.github.io/paper_mgidi_pm/. The source code used to produce the static website and the results in this manuscript have been archived at 10.5281/zenodo.7155173 as manuscript v2. [file 13007_2022_952_MOESM1_ESM.zip › TiagoOlivoto-paper_mgidi_pm-11ef6c1/docs/sup_figures_files/figure-html/unnamed-chunk-7-1.png]

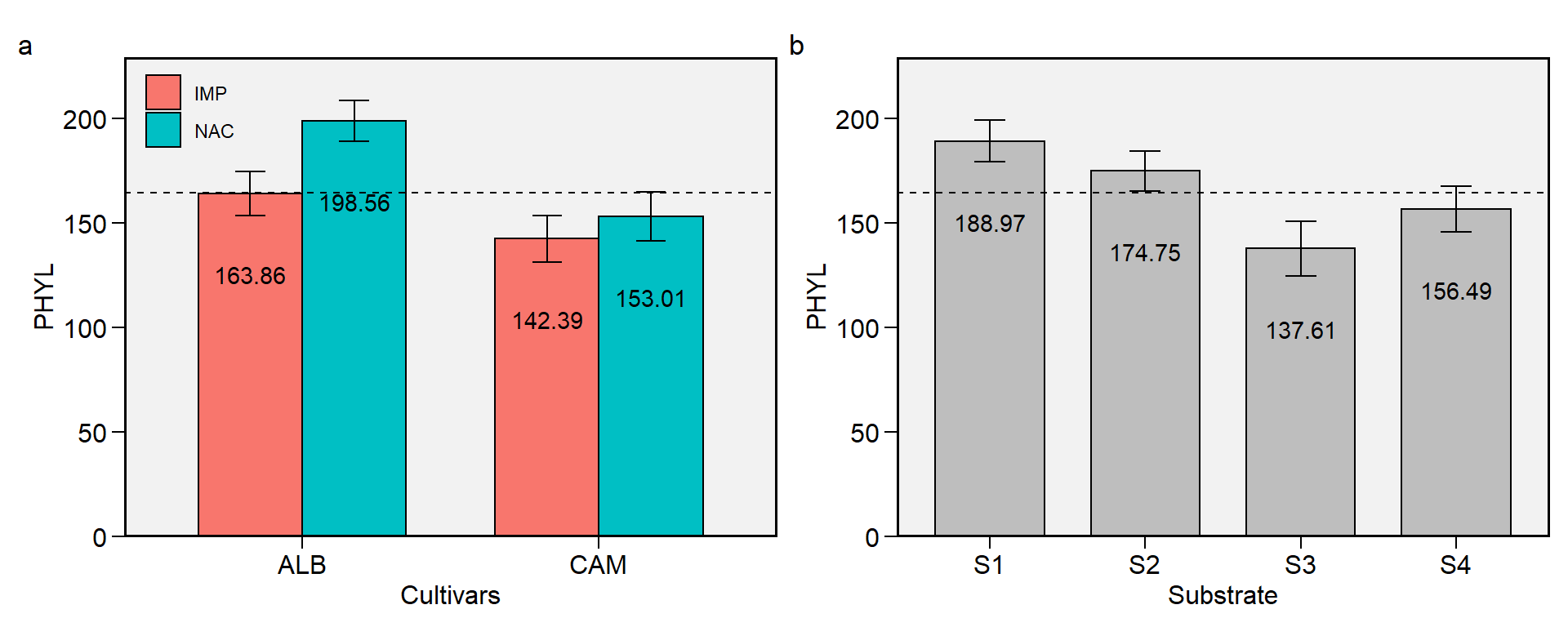

Supplement: Supplementary file 1 — Additional file 1. A website with the data, script, and results is available at https://tiagoolivoto.github.io/paper_mgidi_pm/. The source code used to produce the static website and the results in this manuscript have been archived at 10.5281/zenodo.7155173 as manuscript v2. [file 13007_2022_952_MOESM1_ESM.zip › TiagoOlivoto-paper_mgidi_pm-11ef6c1/docs/sup_figures_files/figure-html/unnamed-chunk-8-1.png]

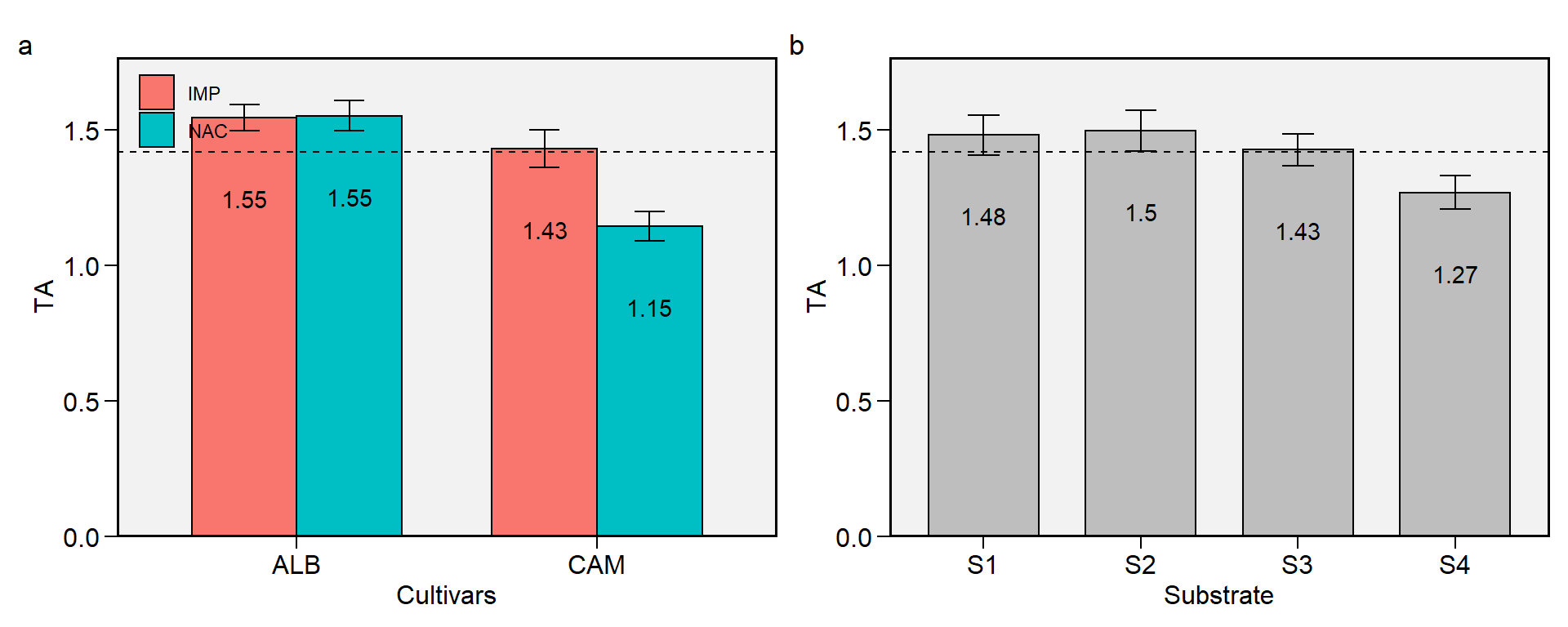

Supplement: Supplementary file 1 — Additional file 1. A website with the data, script, and results is available at https://tiagoolivoto.github.io/paper_mgidi_pm/. The source code used to produce the static website and the results in this manuscript have been archived at 10.5281/zenodo.7155173 as manuscript v2. [file 13007_2022_952_MOESM1_ESM.zip › TiagoOlivoto-paper_mgidi_pm-11ef6c1/docs/sup_figures_files/figure-html/unnamed-chunk-9-1.png]
